# Scoping Review of Factors Affecting Antimicrobial Use and the Spread of Antimicrobial Resistance in the Poultry Production Chain

**DOI:** 10.3390/vetsci12090881

**Published:** 2025-09-12

**Authors:** Zsuzsa Farkas, Orsolya Strang, Andrea Zentai, Szilveszter Csorba, Máté Farkas, András Bittsánszky, András Tóth, Miklós Süth, Ákos Jóźwiak

**Affiliations:** 1 Institute of Food Chain Science, Department of Digital Food Science, University of Veterinary Medicine Budapest, 1078 Budapest, Hungary; farkas.zsuzsa@univet.hu (Z.F.); zentai.andrea@univet.hu (A.Z.); csorba.szilveszter@univet.hu (S.C.); farkas.mate@univet.hu (M.F.); bittsanszky.andras@univet.hu (A.B.); toth.andras.jozsef@univet.hu (A.T.); suth.miklos@univet.hu (M.S.); jozwiak.akos@univet.hu (Á.J.); 2 National Laboratory of Infectious Animal Diseases, Antimicrobial Resistance, Veterinary Public Health and Food Chain Safety, University of Veterinary Medicine Budapest, 1078 Budapest, Hungary; 3 Institute of Food Chain Science, University of Veterinary Medicine Budapest, 1078 Budapest, Hungary

**Keywords:** antimicrobial resistance, poultry production chain, farming practices, antibiotic use, knowledge attitudes and practices, environmental factors, poultry farming

## Abstract

Antimicrobial resistance (AMR) is a critical global health concern, with the food production sector, particularly poultry production, playing a significant role in the spread of resistant bacteria. This study aims to identify and analyze the key factors influencing antimicrobial use (AMU) and thereby/or the spread of AMR within the poultry production chain, spanning from farm to consumer. A scoping literature review was conducted to investigate factors influencing AMU and/or the emergence and dissemination of AMR in poultry production. Knowledge synthesis was conducted from 69 relevant publications covering data from 41 countries. A total of 327 factors were identified from the various studies. Most factors (221) were related to knowledge, attitudes, and practices (KAPs) concerning farms. The other significant group, with 57 factors, involved intentional misuse or poor practices related to antimicrobials. The findings highlight the complexity of factors influencing AMR in poultry production, including financial pressures on farmers and varied training effectiveness. Integrated and tailored interventions are essential. Drug/feed sellers’ practices require stronger regulations, while economic motivations and policy gaps must be addressed. Systematic management of these interactions and further research on environmental and genetic influences are crucial for effective strategies to combat this pressing public health challenge.

## Simple Summary

Antimicrobial resistance happens when bacteria stop responding to medicines that once killed them, which is becoming a global health problem. Poultry farming is one of the major contributors to antimicrobial resistance. The review of 69 studies from 41 countries explored what drives the use and misuse of antibiotics in poultry and how that helps resistant bacteria spread from farm to consumer. This quest uncovered 327 factors. Most were linked to what farmers know, believe, and do when it comes to using antibiotics. Others involved poor or intentional misuse, like giving antibiotics when they are not necessary or for growth promotion. The results show how complex the issue is. Farmers often face financial pressure and inconsistent training. Drug and feed sellers may push antibiotics without enough regulation or knowledge. These problems combine to fuel the spread of resistance. To fight antimicrobial resistance, smart, targeted actions, like better rules for sellers, more support for farmers, stronger policies, and more research, are needed. In short, tackling antibiotic resistance in poultry farming is not about one solution; it is about understanding the web of factors and acting on multiple fronts to keep people and animals safe.

## 1. Introduction

Antimicrobial resistance (AMR) is a global challenge that the whole world is facing nowadays. The resistance of pathogens to important drugs may impair the success of treatment both in humans and animals. Recent studies estimated that 127 million deaths were directly attributable to AMR in 2019, with about 33,000 annual deaths in the EU alone. AMR was declared as one of the top 10 global public health threats by the World Health Organization (WHO) [1,2]. According to the O’Neill report [3], 10 million people might die annually because of antibiotic resistance by 2050, exceeding the mortality rate caused by cancer, if the development rate of resistant bacteria continues to increase. Treatment failure may lead to huge economic losses in the food chain as well.

Global demand for meat and meat production is steadily increasing, particularly as low-to-middle-income countries (LMICs) transition to higher incomes [4]. The intensification of animal production is accompanied by the use and misuse of antimicrobial drugs.

Poultry is among the most consumed meats worldwide and the second most produced and consumed in the EU [5]. The Food and Agricultural Organization (FAO) reported a global increase in poultry meat production worldwide, amounting to 143.5 million metric tons (39% of global meat production, 365.5 million metric tons) in 2022 [6]. Chicken is the most commonly farmed species, with over 90 billion metric tons produced annually [7]. Chicken is also the most exported type of meat, and the poultry industry is expected to lead global meat production in the coming decade [8].

A range of antimicrobials are used in poultry, mostly orally, mainly to treat and prevent diseases, but in certain, mainly developing countries, they are also used to enhance productivity and growth [7]. The success of poultry farming is influenced by various factors, including the efficacy of antimicrobials; however, from a health standpoint, AMR represents a more critical challenge.

Quinolones and fluoroquinolones are classified as critically important antimicrobials for human medicine. Research conducted by Perrin-Guyomard et al. from 2011 to 2018 revealed a 71.5% decline in fluoroquinolone exposure among French poultry; however, resistance patterns exhibited contrasting trends across the bacterial populations studied. [9]. In the work of Chalmers et al., extended-spectrum cephalosporin (ESC)-resistant *Escherichia coli* were isolated from broiler chickens raised conventionally and without therapeutic antimicrobials in Canada. Flocks raised with or without antimicrobials contained similar ESC-resistant *E. coli*, the proportion of positive samples was high, and ESC resistance remained widespread despite the fact that the use of ceftiofur ceased in the Canadian poultry industry in 2014 [10]. Colistin resistance remains a significant concern in broiler populations despite regulatory interventions. The study of Islam et al. found a high prevalence of phenotypic colistin resistance (64.6%) and plasmid-mediated mobilized colistin resistance genes (mcr) carriage (36.4%) in poultry-chicken isolates, strongly linked to prior colistin use in farms [11]. Similarly, the study of Kawano et al. revealed persistent mcr-1.1-positive *E. coli* (13.6% of isolates) in broilers 6 years after a national ban on colistin as a feed additive [12]. Both studies highlight the challenge of eliminating colistin resistance once it has emerged, even after usage restrictions. Although AMU is a well-known driver of AMR, in the case of both laying hen and broiler chickens’ commercial farms, it does not seem to be the only factor responsible for the persistence of extended-spectrum beta-lactamases and AmpC beta-lactamases (ESBL/AmpC) producing *E. coli* at the farm level. Therefore, Aldea et al. suggest that other farm management strategies should be implemented to control the spread of AMR in addition to proper AMU [13].

Several studies focused on the prevalence of AMR in commensals (*E. coli*, *Enterococcus*, *Staphylococcus*) and zoonotic bacteria (NTS, *Campylobacter*) in poultry [7]. Commensals may represent a reservoir for resistance genes, and, although not directly causing illness to the host, may transfer AMR to pathogens. Studies have shown that production animals usually have antibiotic-resistant bacteria in their gut, on mucous membranes, and on their skin [14].

Antimicrobial resistance is a natural, ancient, and widespread occurrence among bacteria, since several organisms are capable of producing antibiotics, e.g., fungi and soil bacteria. AMR was not created by humans but rather promoted by applying evolutionary pressure on the ecosystem, such as AMU. AMR can be inherent, mutation-allied, or attained through horizontal gene transfer. Bacteria can overcome the effect of antibiotics through intrinsic or acquired (with the help of transformation, transduction, or conjugation of mobile genetic elements like transposons and plasmids carrying the antimicrobial resistance genes (ARGs)) mechanisms. AMR usually results from changes that affect the drug–target interaction, driven by four general mechanisms: efflux pump, reduction in permeability to antimicrobials, inactivation of antimicrobials, and modification of drug binding targets [15,16].

Humans can acquire antibiotic resistance via different pathways, including human-to-human transmission, contact with animals, the food chain, and the environment [17].

Evidence has emerged highlighting the contribution of antimicrobial use (AMU) in animal production to the transfer of resistant bacteria from animal to human [18]. Overall, 80% of antimicrobial agents produced in the US are applied in animal production, and this rate is over 70% globally [4,19,20]. The amount of antimicrobials used in livestock was estimated at 99,502 metric tons in 2020 and expected to increase by 8% to 107,472 metric tons by 2030 [21].

In our study, we focused on all the potential factors that may contribute to the emergence and spread of AMR in the poultry production chain, which is the end-to-end system linking genetic selection, input sourcing, health management, farming, processing, and distribution to deliver safe, quality poultry products (meat/eggs) while applying veterinary oversight, disease prevention, and biosecurity measures. In this study, ‘poultry’ encompasses all domesticated birds raised for meat or eggs, including broilers (chickens for meat), layers (egg-producing hens), turkeys, ducks, and other species. While AMU is a primary driver of AMR, this review examines factors influencing both phenomena due to their interconnectedness in the poultry production chain. We maintain this distinction throughout, using AMU to refer specifically to antimicrobial application practices and AMR to denote the resultant resistance traits in microorganisms. Therefore, a scoping review was conducted to collect and evaluate these influential factors and their effects on AMR. In our conceptual framework, a *driver* is identified as having an increasing effect on the target (in this case, specifically AMU and AMR), while a *factor* has the potential to exert both negative and positive effects.

## 2. Materials and Methods

### 2.1. Review Approach

The review strategy was designed based on the guidance of review methodologies applicable in the related field [22,23,24]. The basic scoping review methodology was adjusted to be fit for purpose, e.g., the search was systematic, detailed analyses were prioritized, and two reviewers conducted the relevance confirmation and data extraction steps. Meta-analysis was not performed.

The primary data source was peer-reviewed scientific articles of primary research. The intention was to provide results from the findings of the publications (can be seen in Supplementary Material File S1). This review was performed in accordance with the PRISMA (Preferred Reporting Items for Systematic Reviews and Meta-Analyses) guidelines.

### 2.2. Review Team

The core review team consisted of four individuals with expertise in related topics (agriculture, food safety, microbiology, and veterinary public health) and methodology(knowledge synthesis). The method-related activities were implemented and executed by the core team members, who met regularly throughout the review procedure. Prior to implementing the review, the review protocol, the proposed approach, and the inclusion and exclusion criteria for the screening and selection of relevant articles were shared with members of the group for feedback.

### 2.3. Review Question, Scope, and Eligibility Criteria

The key review question was “Which factors are contributing to the use of antimicrobials and development of AMR in the poultry production chain?”. It was framed using the PICO (Population, Intervention, Comparison, Outcome) process, which is also widely used in human health systematic reviews.

The population of interest included poultry, with the main focus on chickens. Intervention was the factor affecting AMR and/or AMU. The comparison factor was no change versus an increase or decrease in AMR, and the outcome was AMU and/or spread of AMR.

### 2.4. Search Strategy

Studies were identified by searching the Scopus [25] online database. The keyword selections comprised a combination of terms related to the population of interest and to the influence/effect, so the following general form was used: [keywords to factors contributing to AMR in poultry (e.g., antimicrobial resistance OR AMR); all separated by OR] AND [types of the population of interest (e.g., poultry OR chicken OR *Gallus* OR broiler); all separated by OR] AND [types of the influence/effect (e.g., factor OR driver); all separated by OR].

Searches were run for publication title, abstract, and keywords and were restricted to those published in English, from 2013 to 2023 February (the last 10 years from the date of search).

More details on the search strategy are reported in Appendix A, Table A1.

### 2.5. Title and Abstract Relevance Screening (AS)

Title and abstract screening were conducted based on relevance to the research question in ASReview Lab software (Version v1.0.3) [26]. Abstract selection has been performed by one independent reviewer. In cases of ambiguity regarding the relevance of a publication based on the content of the abstract, the decision was made by an independent supervisor.

The inclusion and exclusion criteria for the title and abstract relevance screening are summarized in Appendix B, Table A2 (Title and Abstract Relevance Screening Form).

### 2.6. Relevance Confirmation During Full Text Screening

Papers selected during the title and abstract relevance screening were accessed as full-text articles, and a relevance confirmation was performed with the help of a pre-defined Full Text Relevance Confirmation Form (Appendix C, Table A3) by two reviewers in the Zotero web application [27]. During this phase, papers that did not contain data referring to factors contributed to AMU or AMR and also reviews and book chapters were excluded. Papers passing this stage were assessed in detail, and results were extracted from them in a subsequent step.

### 2.7. Content/Information Extraction

As the main objective of the study was to summarize the factors contributing to AMR in the poultry production chain, information on factors leading to AMR directly or indirectly was extracted from the selected papers. The extraction was performed by two reviewers with the help of a data/information extraction form (Appendix D, Table A4). The form included data fields on paper identification (authors, title, publication details). For the knowledge synthesis, there was mandatory and optional information extracted from the papers. Drivers resulted in increased AMR; factors affecting AMR were a must-have. Additional data concerning the affected bacterium, antibiotic or antibiotic group, disease type, AMR extent, sample type, point in the food chain, geographical location, comments regarding the study, and the target/outcome of the experiment were extracted if mentioned in the studies.

## 3. Results

### 3.1. Results of the Review Process

**Figure 1:**
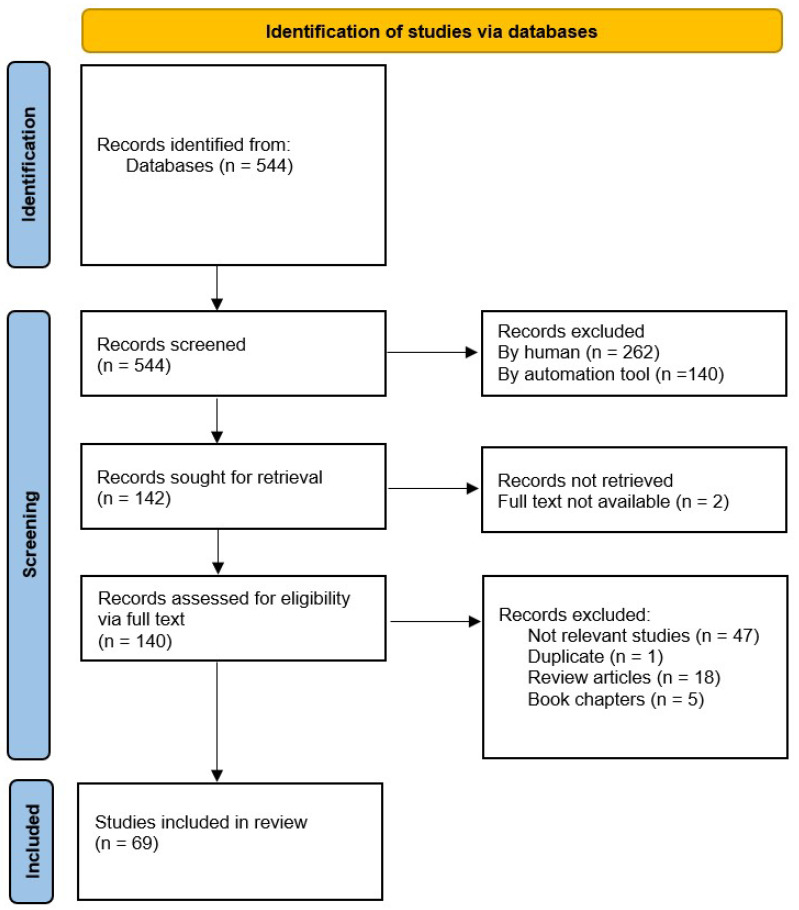
PRISMA Scoping review knowledge synthesis process flow diagram.

A process flow diagram of the knowledge synthesis process for the review is shown in Figure 1. The search strategy resulted in 544 articles. After the screening process (Section 2.5 and Section 2.6), 69 articles were selected for knowledge synthesis.

### 3.2. Classification of Factors Affecting AMU and the Spread of AMR

**Figure 2:**
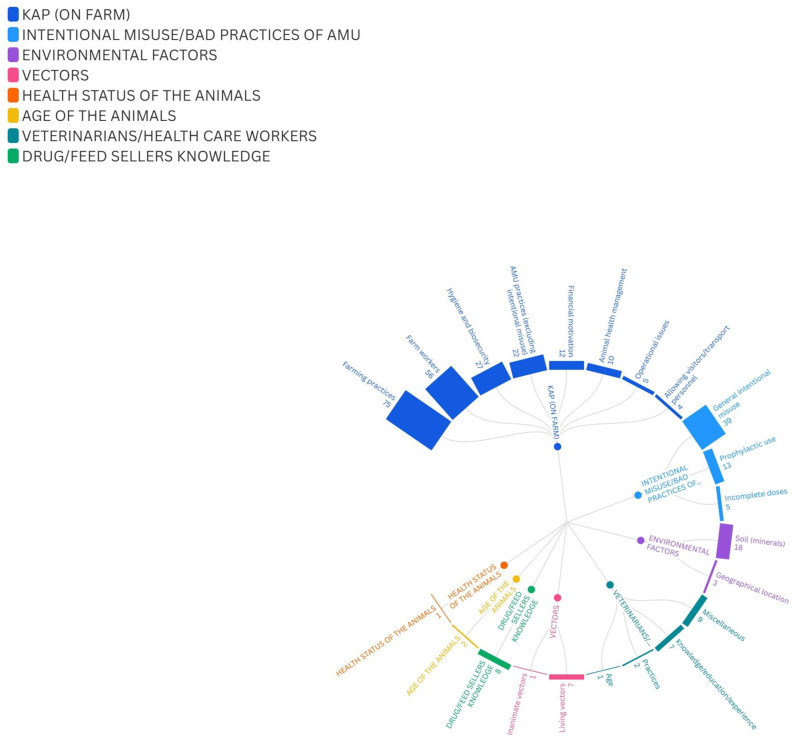
Distribution of the number of factors. The figure shows the two-level classification of the identified factors. In the inner part, with different colors of circles, the main categories are represented. On the outer section, matching colored columns represent the sub-classes belonging to the same main categories, with the number of factors.

**Figure 3:**
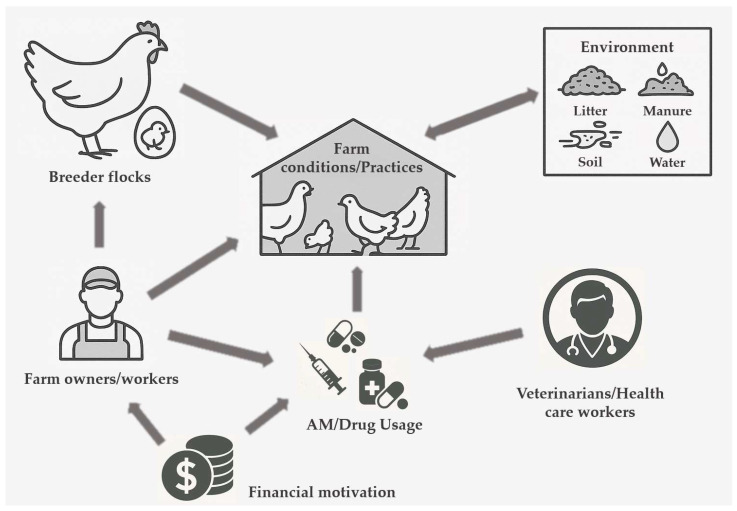
Network of AMR intervention points based on the identified factors.

In our study, we aimed to systematically categorize the factors influencing AMU and the spread of AMR. By structuring the factors in this way, we provide a clearer framework for understanding the results of the review. To achieve this, we first compiled a comprehensive list of all identified factors from the relevant literature. This list contained a total of 327 identified factors from the different publications. Subsequently, based on the expert judgement of the authors, we organized each of these factors into a hierarchical classification system. Thus, at the highest hierarchy level, the factors were grouped into the following 8 main categories: knowledge, attitude, and practice (KAP) (on farm) (211 factors), intentional misuse/bad practices of AMU (57 factors), environmental factors (21 factors), vectors (8 factors), health status and age of the animals (3 factors), veterinary/health care workers (19 factors), and drug/feed sellers’ knowledge (8 factors). The hierarchical classification and the number of studied factors identified in the publications are illustrated in Figure 2. Figure 3 shows a schematic AMR transmission pathway based on the identified factors.

Most of the publications contained information about factors of KAP related to farms or farm workers (52 different publications). The other significant group of factors, with 24 publications, was intentional misuse or bad practices of AMU. Other factors (addressed in 26 publications) were environmental factors, vectors, health status, and age of the animals or were related to the knowledge, attitude, and practice of veterinarians/health care workers or drug/feed sellers. In some cases, the same publication dealt with differently classified factors.

### 3.3. Discussion on Factors Affecting AMU and the Spread of AMR

In this section, the results of the content/information extraction are discussed. Unexpected or unclear results found in the studies are discussed in more detail. The results of the selected relevant studies regarding factors affecting AMU and AMR, in terms of their increasing or decreasing effects, are synthesized. A more detailed description of the relevant studies can be found in Supplementary Material File S1, which extracts essential information about the relevant parts of all publications included in this study.

#### 3.3.1. Knowledge, Attitude, and Practice

The most extensive and thoroughly studied area regarding factors affecting AMU and/or AMR is the knowledge, attitude, and practice of farms and farm workers. Many studies were conducted in developing countries. The methodology primarily involved interviews (e.g., individual or focus group discussions).

##### Farm Workers’ Knowledge, Attitude, and Practice

1. Knowledge and attitude

Regarding the knowledge and attitude on farms, factors increasing the risk of AMU and/or AMR included the lack of general understanding and awareness of the farm workers [28], lack of familiarity with the term of antibiotic [29], carelessness among livestock farmers [30], inadequate knowledge about infection prevention and control of animal diseases [31], and personal beliefs about AMR regarding the veterinary contribution to AMR [32]. Weak knowledge about antibiotic use was considered a risk-decreasing factor because it resulted in lower usage of antibiotics [33]. Knowledge about the animal health authority and about antiparasitic drugs (if the participants could name one) was also considered as a decreasing factor [34].

2. Education and training

Regarding the level of education of farm workers, a higher proportion of the results in the studies yielded the expected outcomes—meaning that the higher the education level the workers have, the lower the level of AMU and AMR, and vice versa [35].

Education level may affect AMU and AMR in a more direct way, e.g.:

- The more proper use of antibiotics (complying with withdrawal times before slaughtering, using diagnostic tests before using antimicrobials, using more than one antimicrobial in a treatment therapy [36], reducing AMU [31]);
- The proper use of veterinary services (using a qualified veterinarian [37] or seeking advice before using antimicrobial therapy [36]).

The other way education level affects AMU and AMR is indirect—it can be correlated with general knowledge about antibiotics [34], with KAP in general [17,38,39], or with specific factors of KAP (better attitude as a separate factor [40] or knowledge, attitude, and risk perception considered together [41]).

The training of farm workers is also associated with higher levels of general knowledge [39,42] and more prudent attitudes [42]. The lack of training in farming was associated with the presence of multidrug-resistant *E. coli* [39].

3. Unexpected results regarding the effect of education and training on AMU and AMR

In a study conducted in Burkina Faso, a high level of education negatively influenced knowledge and was associated with using antibiotics for prevention and growth promotion [37]. According to the survey, respondents with higher education levels tended to use antibiotics more frequently for both prevention and growth promotion, despite the misuse of these drugs. This trend may stem from the increased financial means of middle-class farmers. After receiving prescriptions, respondents often exceeded the recommended usage, driven by the desire to safeguard farm income against potential epidemics.

In a study conducted in the Dakar region of Senegal [43], farms with staff with reported veterinary skills had a greater occurrence of resistant *E. coli* isolates. According to the authors, this implies that breeders with such knowledge use more antimicrobials. In Senegal, where most farmers have basic breeding training, they may overstep their roles by prescribing and using antimicrobials, often in suboptimal doses.

The level of education was associated with the frequency of using antimicrobials and with performing group treatment [31]. The focus group discussions, from which the results originated, were conducted in Tanzania. The contradictory results can be explained by multiple correlating factors. Farmers were educated to some extent, and they diagnosed sick animals without engaging professional help, as it would have been expensive. Also, the drugs were easily accessible, and sellers encouraged buying them for the sake of profit.

Providing information solely on AMU and AMR to agrovets is unlikely to promote more prudent dispensing practices [42]. In fact, this type of training was linked to a 3% decrease in prudent practices. However, it was positively associated with knowledge and attitude in the same study performed in Zambia.

The transferability of these findings to other regions with different epidemiologic, legal, and socio-economic environments requires caution.

4. Farm workers’ experience

Most of the associations regarding poultry farming experience were as expected—it was generally positively associated with the decrease in AMR [44,45]; with the prudent use and proper sourcing of antimicrobials [31]; with KAP [42]; and with KAP, AMU, and AMR [17].

On the contrary, in a study conducted in Nigeria, respondents with longer periods of experience in poultry farming (25–35 years of experience) were less likely to have satisfactory attitudes to the practice of AMU and AMR [41]. Experience in poultry farming in Cameroon was also negatively associated with practices that eventually resulted in the increase in the spread of AMR [39].

5. Farm workers’ personal characteristics (age, gender, marital status, income, location, and health status)

There was only one study in the examined period dealing with the marital status of farmers: the single and separated marital status of farmers is associated with knowledge, attitude, and risk perception regarding AMU and AMR [41].

One study examined the effect of the health status of farm workers; diarrhea in poultry workers in the last 3 months increased the risk of AMR [46].

Two publications contained information related to the gender of farm workers. There was a negative association between female farmers and certain agricultural practices [39]. Additionally, farms managed by male farmers were linked to higher levels of AMU, which increased the risk of AMR [47].

Regarding the farm workers’ age, the results were divisive. Four studies found that greater age can be associated with a decrease in AMR, e.g., correct KAP [17,42,44] or the use of antibiotics [33]. However, three publications reported contradictory results. One study found that younger farmers can be associated with a satisfactory attitude [41], while another indicated that age is negatively associated with knowledge, practices, and risk perception [39]. Additionally, an association was identified between the length of occupational exposure and the presence of multidrug-resistant (MDR) *E. coli* [46]. Furthermore, the effect of farmers’ locality was positively associated with knowledge about AMU and AMR [39].

There were congruent results regarding the effect of farm workers’ income on AMU and AMR. Weak financial status [30,35] and livelihood precarity [48] have been associated with increased AMR. Conversely, one study found that the monthly income of farmers positively correlated with their knowledge of antibiotics [34], while another indicated that higher income was positively associated with correct knowledge, attitudes, and practices (KAP), which may indirectly contribute to a decrease in AMR [17].

##### Farming Practices

1. Management of sewage, litter, manure, bedding, carcasses, and water

Sewage samples were significantly associated with AMR (specifically with resistant *E. coli*) in the participating states of East Coast Peninsular Malaysia [49].

Storing manure on the farm [50] and composting it [51] were linked to a decrease in AMR. However, a contradictory finding associated the transfer of feces and carcasses with higher biosecurity scores, which might decrease the risk of AMR on the farm [52]. In contrast, disposing of dead chickens into the environment has been identified as a factor that increased AMR [43]. Additionally, the use of animal manure in agricultural activities has been shown to contribute to increased AMR [31].

The effect of chicken litter on increasing the risk of AMR has been confirmed, with findings indicating that chicken litter harbors a high number of resistant bacterial strains [45,53]. Additionally, the risk of AMR has been associated with litter previously used by chickens for turkey rearing [54]. Residual concentrations of antimicrobial growth promoters in poultry litter have been shown to favor plasmid conjugation among *E. coli* [55]. Moreover, wood shavings used as litter have been linked to an increased risk of AMR, while certain bedding materials, such as shredded straw, have been found to decrease this risk [56].

Regarding water management, different increasing factors were identified, such as the use of artesian wells [57], pump water/surface water [49], river water for irrigation [31], and the same water used for washing hands and utensils by meat vendors [58].

2. Flock/herd related effects (arrangement, size, density)

It was not disputed by the researchers that conventional raising of chickens, animals kept in a cramped space [48], and animal confinement [59] increase the risk of AMR compared to free-ranged poultry production [35,60]. Although battery cage systems are viewed negatively from an animal welfare point of view, deep litter systems have a higher risk regarding AMR [61].

Other identified factors associated with ARG abundance were the weight of the broilers at setup and the average rounds per year [52]. Farms with two or more chicken houses were identified as a risk for AMR [43]. On the other hand, keeping livestock penned [38], shorter growth duration/cycle (<40 days), and higher broiler stocking batch [41] were linked to better KAP parameters and thereby to the decrease in AMR. All-in-all-out farming systems were also found to have a positive effect on the decrease of AMR [47,62].

Large flock size has been shown to be a risk factor for AMR [63,64] as it tends to increase the use of antimicrobials. However, an association was found not only with large flock sizes (5000 to 10,000 hens per herd) but also with flocks of under 5000 hens, attributed to over- and underdosage of antibiotics, respectively [65]. Additionally, a flock size of 700 to 1200 was identified as being at higher risk for AMR, while the lowest risk was associated with flocks of fewer than 700 and the highest risk with flocks larger than 1701 [61].

High density of animals was considered a risk factor for AMR [66] and was associated with bad practice [39] and overdosage of antibiotics [65].

3. Farm-related effects (size, location, number of farm workers, equipment used, feeding practices)

The effect of the number of farm workers was studied in two publications. An association was found between the number of farm workers and the relative abundance of ARGs [52]. It was concluded that a livestock ratio of fewer than 2000 hens per employee led to an overdosage of antibiotics [65]. Additionally, the relative abundance of four ARGs in broiler feces was studied using a mixed model to evaluate the relationship between AMR, AMU, and other farm characteristics [52]. With regard to the univariable analysis and the multivariable model without the AMU of broilers, significant (*p* < 0.05) positive associations were found between relative ARG abundances and the number of farm workers. The authors speculated that farm staff could act as a source of ARGs for farm animals, as was previously described for methicillin-resistant *Staphylococcus aureus* (MRSA CC398) in pig farms in Norway. They noted, however, that there had only previously been occasional reports on the introduction of specific resistant bacteria into animal farms, and the transmission was mostly documented from animals to workers.

Regarding farm location, closely located farms [57] and neighboring poultry and/or swine backyard production systems (BPS) [67] were considered increasing factors.

When examining the effect of farm size on AMR, small farm size and small-scale farming were increasing factors for AMR [35,49,62], pointing out the dependence of small-scale farms on antibiotics [48,67]. These results contradicted that in Vietnam, AMU was significantly higher in industrial farm sizes than in smaller farms [36] and that smaller-scale farms were associated with better attitudes [38]. Larger farm sizes were considered as decreasing factors in many studies, linked to correct KAP, such as consultation with veterinary services, knowledge, etc. [17,34,36,37].

Low-quality feed [59], the use of commercial feed [41,62], antibiotics in feed [48], and self-compounding feed milled at a mill [41] were identified as factors that increase AMR. Contrary to previous assumptions, zinc (Zn) and copper (Cu) reduce the conjugative transfer of resistance plasmids in ESBL-producing *E. coli*. ESBL-producing *E. coli* strains isolated from retail chicken meat were used as plasmid donors in growth media containing elevated levels of ZnCl_2_ (0.05, 0.125, 0.25 mg/mL) and CuSO_4_ (0.01, 0.255, 0.5 mg/mL), reflecting concentrations found in chicken intestines. The investigation focused on the conjugation of two plasmids (IncK and IncI1) carrying the antibiotic resistance gene *bla_CMY-_*_2_. ZnCl_2_ and CuSO_4_ reduced the conjugation frequency between *E. coli* strains in a concentration-dependent manner by reducing the expression of the conjugation genes traB and nikB. Conjugation of the IncK plasmid was reduced by more than 98% at all concentrations of ZnCl_2_ and the two highest concentrations of CuSO_4_, while the two highest concentrations of ZnCl_2_ and CuSO_4_ reduced the conjugation of the IncI1 plasmid by more than 90% [68].

Vehicles and instruments as vectors of resistance were identified [57].

4. Breed selection

Raising chickens for meat or mixed purposes (and not solely for egg-laying purposes) [47,65], broiler birds compared to dual-purpose birds [61], and fast-growing breeds were indicated as risk factors for AMR [69]. Cobb lineage and a mixture of Cobb and Ross lineage versus Ross alone increased the odds of fluoroquinolone-resistant *FQr Campylobacter jejuni* [64].

5. Species per farm

Regarding species per farm, different types of factors were identified. Specialized farms with only one species have been identified as risk factors for AMR [33]. Close contact between different animal species in Chilean BPS has been noted as an increasing factor for AMR [67], while the presence of pets in these systems has been associated with a decreased risk of antibiotic use and, consequently, AMR.

6. Administration/record keeping

Keeping records was associated with knowledge on AMR [40,42] and a better attitude [38].

##### Animal Health Management

Adequate disease management practices were linked to the decrease in AMR [52]. Risk-increasing factors found in the literature included a high frequency of digestive tract diseases [45] and the recognition of diseases in animals, increasing the probability of antibiotic use by over 9.38 times. This could be explained in two ways: either BPS owners are aware of the disease and also know how to take care of infected animals or these treatments are due to a lack of knowledge on how to treat diseases, leading to a potential misuse of antimicrobials in these settings [67]. Inadequate consultation with animal care personnel was associated with lower knowledge on AMU [39], which is consistent with the finding that professionals are often perceived as the “last resorts” for animal health, and agricultural/farm supply store (agrovet) employees are often the first sources of health advice sought by farmers [42]. Both studies were conducted in African countries (Cameroon and Zambia). Adding minerals and diuretics together with antibiotics has been found to be a risk factor for AMR [39]. Additionally, factors such as group treatment, easy access to antibiotics, home storage of veterinary antimicrobials, and diagnostic methods (including misdiagnosis and over-/under-dosing based on clinical symptoms) have been associated with increased AMR [31].

##### AMU Practices and Their Impact on AMR (Excluding Intentional Misuse)

There were two studies examining the type of administration of antibiotics. In ovo use of ceftiofur has been identified as an AMR increasing factor [50], as well as the administration of antimicrobials through drinking water [35].

Many studies linked the general use of antimicrobials as an AMR-increasing factor [52,70,71,72,73,74,75]. Numerous studies associated AMR with specific antibiotics in poultry production (enrofloxacin [76], ceftiofur [50,54,70], tetracycline, gentamicin, bacitracin, tylosin [54], oxytetracycline, and streptomycin [70]). Fluoroquinolone (FQ) use in the past has been identified as an AMR increasing factor [77], while colistin administration had no effect on AMR [51].

In a more specific study regarding this topic, quinolone and tetracycline usage was a factor for ciprofloxacin resistance in *E. coli*, lincosamide and tetracycline usage was a factor for gentamicin resistance in *E. coli*, the usage of any antimicrobial drug was a factor for third generation cephalosporin resistance in *E. coli*, and tetracycline usage is a factor for quinolone resistance in *E. coli* [62].

There was no difference between antimicrobial-free and conventional farms regarding resistance. The withdrawal of individual compounds, such as cephalosporins and fluoroquinolones, increased the use and resistance levels of other drug classes, such as aminoglycosides [78].

##### Hygiene and Biosecurity

Many studies have concluded that poor biosecurity and hygiene, in general, increase AMR risk [36,39,42,52,56,59,63]. The more specific risk factors identified in this area are the following:

- Absence of a lavatory for workers [46];
- Absence of specific shoes for staff [62,79];
- Use of hydrogen peroxide to disinfect water lines during the growing period [50];
- Transport personnel entering the room where the broilers are raised [80];
- Personal movement, vehicles, and instruments (considered as vectors) [57];
- Disposal of solid wastes from the household [31];
- Uncontrolled disposal of human and veterinary drugs [31];
- Lack of observance of an empty period of the flock house [43];
- Positive status for diseases of the previous flock in the broiler house [80];
- Uncontrolled wild bird movement [50,77]

The AMR decreasing factors identified were the following:

- Masks provided for staff [77];
- Detailed areas dusted before wet cleaning [77];
- Feed hoppers cleaned and disinfected [77];
- Sanitation of the stall environment (significantly related to bacterial contamination) [58];
- Routines for disinfection of the floor between production cycles [80].

There was only one study with contradictory results, as a small to no effect was found when using an ideal method for cleaning and disinfection [78]. Additionally, no effect of decontamination of eggshells on AMR was reported [81].

##### Financial Motivation

The results of many studies proved the financial motivation and economic considerations behind the misuse of antibiotics, primarily for preventive purposes [32,38,42,82]. Farmers’ economic conditions influence antibiotic prescription [83], and antibiotics are often misused to shorten farming periods [31]. Unequal market conditions have been emphasized, with most farmers purchasing day-old pre-vaccinated chicks or fully grown laying chickens from large poultry producers, which also sell fully grown broilers and eggs for consumption at lower prices, undercutting local farmers [48]. Inherited livestock farms could be risk factors for AMR, especially when livestock production is the primary source of income due to antibiotic use [84]. Economic reasons deterred farmers from calling veterinarians [59], and the cost of veterinary advice and antibiotics often led to underdosing or slaughtering sick animals [48]. Additionally, purchasing day-old chickens from sources other than industrial hatcheries, such as local hatcheries, markets, or neighbors, also contributed to AMR [62].

##### Operational Issues

Company-owned production has been found to present a lower risk for AMR compared to contractual production [41]. Additionally, sites operated by independent growers are associated with a decreased risk, while single-handed operations increase the risk for AMR [77]. Both intensive (small-scale production) and extensive (free-range) husbandry management systems have been identified as risks for AMR due to the likely misuse of antibiotics [35]. Furthermore, poor management practices among farmers were also considered risk factors for AMR [32].

##### Allowing Visitors/Transport Personnel

Transport personnel [52,80] and visitors allowed on the site [43,52] or the periphery of the site [77] also increased the AMR risk.

#### 3.3.2. Intentional Misuse or Bad Practices of AMU

Misuse of antibiotics inevitably results in the spread of AMR. The studies included in the review identified the main bad practices and misuse as follows—the order represents the importance of the factor based on the number of studies that identified it:

- Prophylactic (and metaphylactic) use [31,33,35,36,37,38,39,42,65,82,83,85,86];
- Using antimicrobials without a prescription [35,37,42,79,84,85]; they might be bought at supply stores [59] or black markets [49];
- Mistreatment of illness, misuse [29,86] such as no differentiation between antimicrobials and other type of medicines [84], infrequent use of antibiotics to treat parasites or animals not eating [34], use it as painkillers or for treatment of viral diseases [39], administering antibiotics at the first indication of disease [38], constant use of broad-spectrum antibiotic more frequently [87], or use of human antimicrobials in animal treatment [31];
- Improper dosing (underdosing [34,35,40,59] or overdosing [88]) *;
- Poor prescribing practices such as prescription based on telephone conversations, without a history of previous antibiotic use, prescription by unprofessional practitioners [83] or due to the request of the farmers [42] *;
- Availability (unrestricted and easy access) and stocking (irrational sales, hoarding because of former shortage) of antibiotics [30,31,36,59,82,84] *;
- Use for growth promotion [35,36,42,86];
- No observation of withdrawal period [35,37,42,72] *;
- No available guidance/regulation [30,35] and low enforcement of policies and regulations [87] *;
- Poor handling of drugs at purchase and administration practices [87] *.

* note that these might be because of inadequate KAP and not necessarily intentional misuse.

#### 3.3.3. Other Factors

##### Environmental Factors (Soil, Seasonality, Geographical Location)

Concentrations of different minerals such as magnesium, phosphorus, potassium, copper, chromium, manganese, and sodium in soil and feces that contribute to the reduction in multidrug-resistant (MDR) *Salmonella* and *Listeria* spp. were determined [89]. The specific values can be found in Supplementary Material File S1.

For broilers, geographical location, season, and elevation were significantly associated with AMU, with higher AMU observed in Southern Italy, during winter/spring, and in hilly/mountainous areas, all of which significantly affect AMR [66]. The winter season was also associated with an increase in AMR [79]. Conversely, it was found that BPS located closer to sea level had greater chances of AMU [67]. Additionally, the presence of ESBL-/AmpC *E. coli* was linked to a specific region in Uganda, namely Wakiso Town Council, attributed to a higher density of human settlements and differing management practices [61].

##### Vectors

Fly-mediated transmission of the mcr-1 gene, which encodes colistin resistance, from animals and the environment to humans has been identified as a risk factor for AMR [90]. Human and/or wildlife involvement, including pests and wild birds, has been concluded to be the cause of fluoroquinolone resistance, as these antibiotics are excluded from use in Australian livestock [91]. Additionally, there was evidence suggesting potential wildlife involvement in AMR dissemination [71]. The possibility of vertical transmission from parental flocks has also been strongly suggested [92]. Furthermore, broiler hatching eggs have been identified as carriers and potential sources of ESBL/AmpC-producing *Enterobacteriaceae* for broiler chicks [81].

##### Horizontal Gene Transfer

Three studies supported the effect of horizontal gene transfer on the risk of AMR spread. The risk of spreading AMR through conjugation of ARGs carrying plasmids was identified [51,93], while in vitro transduction of ARGs from phages of migratory wild birds into *E. coli* isolates collected from poultry suggested the possible transmission in BPS [94].

##### Health Status/Age of the Animals

It was found that if there was no disease occurring in the past 12 months, the risk of AMR decreased [33].

Young age of birds (4 months and below) [61] and ≥11 days old [95] was identified as a risk factor for AMR.

##### Veterinarians/Health Care Workers’ Knowledge, Attitude, and Practice

Perceptions of professional responsibilities, risk avoidance, lack of compliance with veterinary advice, and shortcomings in the advisory competencies of veterinarians were pointed out as contributing factors to antimicrobial prescribing behavior and thereby risk factors for AMR [32]. Inadequate veterinary extension officers were also considered as risk factors [31]. The level of education of veterinarians, previous training on AMU and AMR [30], and years of experience and age were associated with increased KAP on AMU [83].

There were some miscellaneous risk factors in terms of AMR spread, such as

- Financial dependency on clients and client pressure [32];
- Limited diagnostic facilities [87];
- Lack of access to veterinary services [36,82] or animal health professionals [42].

##### Drug/Feed Sellers’ Knowledge and Motivation

Identified risk factors related to drug/feed sellers’ knowledge:

- Knowledge gap of feed sellers and drug sellers [17];
- Lack of education of supply chain actors, particularly drug retailers [87];
- Lack of awareness of stakeholders about policies that regulate drug use [87];
- Sales agents’ roles as non-professional prescribers of antibiotics [59].

Years of experience, level of education (up to 12th grade), training on the drug, and age of seller (younger) were positively associated with the KAP of the drug and feed sellers [96].

### 3.4. Geographical Representation

Regarding the geographical distribution of the relevant studies included in the publications, the majority were conducted in Europe, Asia, and Africa, with 39.8%, 25.5%, and 17.3%, respectively. North America and South America were represented by 8.2% and 6.1%, while Oceania, Eurasia, and Australia each accounted for 1%.

**Figure 4:**
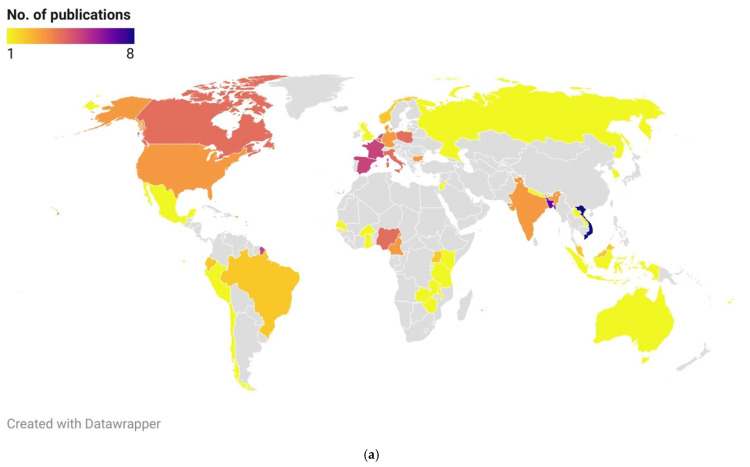

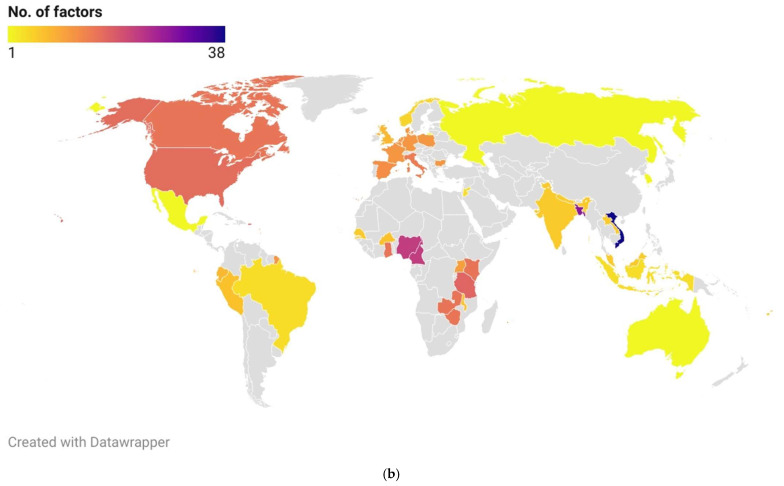
(**a**) Geographical distribution of a number of studies originating from different countries. The most studied countries were Vietnam with 8 and Bangladesh with 7 publications. Canada, most of the European countries, and Nigeria were represented in 4 publications. (**b**) Geographical distribution of a number of factors found in studies from different countries. The most factors—38—were identified in Vietnam, and the second country was Bangladesh with 29 factors. Most of the European countries, the African countries, and Canada and the USA had 11–23 identified factors.

In terms of the distribution of identified factors in the studies, Africa ranked first with 35.3%, followed by Europe, Asia, North America, South America, Oceania, and both Eurasia and Australia, with 27.7%, 22.2%, 7.5%, 5.5%, 1.3%, and 0.2%, respectively. Figure 4a presents a choropleth map illustrating the geographical distribution of studies using color intensity to indicate the number of studies. Figure 4b uses the same graphical representation to show the number of identified factors in the studies. It can be observed that for Asian countries, the distribution of studies and identified factors mainly originates from Bangladesh and Vietnam, whereas for other continents, the distribution is more uniform.

### 3.5. Knowledge/Evidence Synthesis Based on Identified Factors and Conclusions of the Studies

Table 1 shows key drivers of AMU and AMR in poultry production. Key insights include the following:

- Paradox of education: higher farmer education inconsistently reduced AMR (12 studies from the total of 24). In Burkina Faso/Senegal, it increased prophylaxis due to financial capacity;
- Flock management: high density (5 studies) and large size (5 studies) were major AMR drivers, while biosecurity interventions reduced risk (7 studies);
- Economic drivers: financial constraints (11 studies) led to underdosing, non-veterinary AMU, and preventive misuse;
- Environmental transmission: environmental vectors (e.g., flies, wild birds) facilitated AMR gene spread in 8 studies;
- Intentional misuse: prophylaxis (12 studies) and growth promotion (5 studies) dominated, especially in countries where regulations were weak.

In detail, the studied factors, their effects on AMU and/or AMR, the target population, and the location of the studies are listed in Supplementary Material File S2.

## 4. Discussion and Conclusions

### 4.1. General Overview Based on Findings of the Study

The scoping review identifies several interconnected factors significantly influencing AMR within the poultry production chain. KAP among stakeholders is central to AMR dynamics. While higher education levels in farmers often correlate with lower AMR, a critical paradox exists: financial pressures can compel even educated farmers to misuse antibiotics, such as for prophylaxis or improper dosing. Training programs show potential to improve attitudes toward AMR prevention, but their effectiveness varies considerably. Experience in poultry farming yields mixed effects, and personal characteristics like income and farm workers’ location also shape behaviors, with low income consistently linked to increased AMR risk.

The roles of other supply chain actors are pivotal. Veterinarians’ prescribing behaviors, influenced by professional responsibilities and client pressures, directly impact AMR outcomes. Similarly, the knowledge and practices of drug and feed sellers, shaped by their education and experience, contribute to AMR risks, particularly where gaps in policy awareness exist. On-farm practices are major determinants: hygiene, biosecurity, and record-keeping significantly reduce AMR prevalence, with larger farms generally demonstrating better outcomes. Conversely, poor practices like improper carcass disposal and high flock density increase risk. Intentional antibiotic misuse, often stemming from knowledge gaps and weak regulatory enforcement, further exacerbates AMR.

Environmental factors, including geography and seasonality, also influence AMR dynamics, with higher AMU reported in specific seasons and locations. Vectors such as flies and wild birds facilitate the spread of resistance through horizontal gene transfer. Additionally, the health status and age of birds are important factors, with disease-free histories associated with reduced AMR risk.

Key conclusions highlight the complexity of addressing AMR. The paradox between education and practice underscores the need for interventions that address not only knowledge gaps but also the underlying economic pressures driving misuse. The variable effectiveness of training points toward the necessity for more tailored and continuous approaches that account for cultural and regional differences. Strengthening regulations and providing targeted education for drug and feed sellers is critical to mitigate improper antibiotic distribution. Furthermore, certain productive farming practices, like maintaining large flocks, can have unintended negative consequences for AMR if not managed sustainably alongside rigorous biosecurity and waste management. The significant role of financial motivations and weak regulatory frameworks demands policies that align economic incentives with best practices.

### 4.2. Core Insights Based on Knowledge/Evidence Synthesis

Table 2 lists the core insights based on the knowledge/evidence synthesis of the studies. 

**Economic pressures override knowledge:** despite education/training (24 studies), financial imperatives drive misuse in the case of LMICs. For example, 67% of KAP factors (148/221) showed that educated farmers in Senegal and Burkina Faso used more antimicrobials for prevention/growth promotion due to income instability. Small-scale farms in Tanzania and Malawi doubled AMU to offset disease risks.

**Regulatory gaps enable misuse:** intentional misuse (57 factors, 17.4% of total) dominated in regions with weak enforcement (8 LMICs). Prophylactic use (12 studies) and growth promoters (5 studies) persisted despite bans (e.g., 32% of Vietnamese farms). Non-prescription access and poor dosing amplified risks.

**Environmental and structural drivers are understudied**: only 29/327 factors (8.9%) addressed environmental/structural aspects (e.g., vectors, seasonality). Vector-mediated horizontal gene transfer was examined in just three studies. Wildlife (e.g., flies, birds in Australia/Vietnam) and seasonality (winter AMU spikes in Italy) emerged as overlooked amplifiers.

### 4.3. Limitations of the Study

One of the limitations is the geographical representation of the studies. The representation may be disproportionate with regard to certain regions, e.g., Oceania, Eurasia, and Australia were noticeably underrepresented. In the case of Oceania, the cause might be the smaller poultry production industries or less focus on research. While Australia has a significant poultry industry, other factors, like research priorities, might contribute to its limited representation. In Eurasia, despite Russia’s significant poultry production, the overall research focus may still be limited or underreported in the context of AMU and AMR. There are regional disparities in legislative frameworks (e.g., antimicrobial use policies, surveillance requirements) as a potential confounding factor. However, it is critical to note that the study’s aim was not to compare regions systematically but to aggregate global factors influencing AMU/AMR. An additional limitation is the heterogeneity of the included studies, which might vary significantly in design, methodology, and quality, making it challenging to synthesize findings consistently. Because of the general nature of the research question, quantitative analyses or meta-analyses that could provide more robust statistical insights could not be performed. The study acknowledges the multifaceted nature of AMR. Because of this complexity, interactions among factors should be further studied with other methodologies, as it is difficult to determine causality or isolate specific impacts. Finally, the scoping review has only been conducted for one scientific literature database (Scopus).

In general, the study might not cover all potential factors influencing AMR, such as emerging technologies or interventions; however, it reflects the state of the art of recently studied areas related to the scope, due to its inherent systematic procedure (e.g., focus on reproducibility, specified search and filtering steps, timeframe). Publication bias cannot be avoided with the presented procedure.

### 4.4. Final Outlook

AMR in poultry production is a multifaceted challenge, fundamentally intertwining economics, behavior, and ecology. The driving factors—socioeconomic, educational, operational, and environmental—are highly interdependent and interact complexly, offering limited predictive power in isolation and often yielding unexpected outcomes, rendering isolated interventions ineffective. A holistic, systematic analysis integrating epidemiological data with socioeconomic and behavioral insights is therefore essential to unravel contradictions and support risk assessment, forecasting, and strategic planning. Critical areas needing investigation include the underexplored impact of geography and seasonality and horizontal gene transfer via wildlife vectors, highlighting the need for integrated management. Future work must prioritize transdisciplinary frameworks to decode AMR complexity and deliver scalable solutions through integrated policies targeting financial resilience, regulatory accountability, and environmental pathways.

## Figures and Tables

**Table 1:** Key drivers of AMU and AMR in poultry production: evidence synthesis.

Category	Subcategory	Direction	Studies (n)	Example Factor
Farmer KAP	Education/Training	↑↓	24	Higher education → ↑prophylaxis/growth promotion (Burkina Faso); Training → ↓prudent dispensing (Zambia)
	Experience	↑↓	7	Longer experience → ↓AMR (Cameroon); ↑cautious use (Tanzania)
	Age	↑↓	7	Younger farmers → ↑AMU (Lao PDR); Older farmers → ↓AMR (Vietnam)
	Income	↑	6	Low income → ↑AMR (Nigeria, Malawi)
	Gender	↑	2	Male farmers → ↑AMU (Vietnam)
Farming Practices	Litter/Manure Management	↑	6	Chicken litter → ↑resistant bacteria (India, Brazil)
		↓	4	Composting manure → ↓AMR (France)
	Flock Density/Confinement	↑	5	High density → ↑AMR (Vietnam, Cameroon)
	Flock Size	↑	5	Large flocks (5,000–10,000 hens) → ↑AMU/AMR (Cameroon, Canada)
	Water Management	↑	4	River/pump water use → ↑AMR (Tanzania, Malaysia)
	Biosecurity/Hygiene	↑	10	Poor hygiene → ↑AMR (India, Tanzania); Wild bird access → ↑AMR (UK)
		↓	7	Sanitation/disinfection → ↓AMR (Malaysia, EU)
	Feed Practices	↑	4	Low-quality/commercial feed → ↑AMR (Vietnam, Ecuador)
Intentional Misuse	Prophylactic Use	↑	12	Antibiotics for disease prevention (Vietnam, Tanzania, Burkina Faso)
	Growth Promotion	↑	5	Antibiotics to enhance growth (Bangladesh, Indonesia)
	Non-Prescription Use	↑	6	Access without prescription (Bangladesh, Nepal)
	Improper Dosing	↑	5	Underdosing/overdosing (Nigeria, Cameroon)
Economic Factors	Financial Pressure	↑	11	Livelihood precarity → ↑AMU (Malawi); Cost avoidance → ↓veterinary consultations (Ecuador)
Environmental Factors	Vectors/Wildlife	↑	8	Fly-mediated gene transfer (Vietnam); Wild birds → ↑fluoroquinolone resistance (Australia)
	Seasonality/Geography	↑	5	Winter → ↑AMR (Bangladesh); Southern regions → ↑AMU (Italy)
Veterinary Practices	Prescription Behavior	↑	5	Non-diagnostic prescriptions (Ecuador); Client pressure (Netherlands)
Drug/Feed Sellers	Knowledge Gaps	↑	5	Lack of policy awareness (Uganda); Role as non-professional prescribers (Ecuador)
Regulatory Gaps	Drug Access	↑	6	Over-the-counter sales (Nepal); Unrestricted availability (Tanzania)

Notes: ↑ = Increases AMU/AMR risk, ↓ = Decreases risk, ↑↓ = Context-dependent outcomes; Study counts derived from aggregated data Supplementary Material File S2, Tables S1 and S2 (69 studies total); Geographic skew: 75% of evidence from Africa/Asia; limited data from Oceania/Eurasia/America.

**Table 2:** Core insights of knowledge/evidence synthesis.

**Core Insight 1: Economic Pressures Override Knowledge**					
Driver	Category	Subcategory	Direction	Studies (n)	Example Factor
Farmer Decision-Making Drivers	Financial Pressures	Livelihood Protection	↑	11	Trained farmers using antibiotics preventively to avoid income loss (Burkina Faso, Senegal)
		Cost-Driven Practices	↑	9	Economic constraints → Underdosing/slaughter vs. veterinary care (Malawi, Ecuador)
	Production Risks	Flock Security	↑	7	Prophylactic antibiotic use to protect large flocks (Cameroon, Indonesia)
**Core Insight 2: Regulatory Gaps Enable Misuse**					
Driver	Category	Subcategory	Direction	Studies (n)	Example Factor
Systemic Vulnerabilities	Drug Access	Non-Prescription Sales	↑	12	57/327 factors involved intentional misuse (e.g., black market antibiotics in Nepal, Tanzania)
	Policy Enforcement	Weak Implementation	↑	8	Low enforcement → Growth promoter use despite bans (Nigeria, Bangladesh)
	Prescription Practices	Non-Veterinary Advice	↑	6	Drug sellers as primary prescribers (Zambia, Ecuador)
**Core Insight 3: Environmental/Structural Understudied**					
Driver	Category	Subcategory	Direction	Studies (n)	Example Factor
Emerging Risk Pathways	Environmental Exposure	Vectors and Seasonality	↑	5	Fly-mediated gene transfer (Vietnam); winter → ↑AMR (Bangladesh, Italy)
	Farm Infrastructure	Waste Management	↑↓	7	Litter composting ↓AMR (France); improper carcass disposal ↑AMR (Senegal)
	Wildlife Interface	Cross-Species Transmission	↑	4	Wild birds spreading fluoroquinolone resistance (Australia)

Notes: ↑ = Increases AMU/AMR risk, ↓ = Decreases risk, ↑↓ = Context-dependent outcomes; Study counts derived from aggregated data Supplementary Material File S2, Tables S1 and S2 (69 studies total); Geographic skew: 75% of evidence from Africa/Asia; limited data from Oceania/Eurasia/America.

## Data Availability

No new data were created or analyzed in this study. Data sharing is not applicable to this article.

## Supplementary Materials

The following supporting information can be downloaded at: https://www.mdpi.com/article/10.3390/vetsci12090881/s1, File S1: Scoping review of factors affecting antimicrobial use and the spread of antimicrobial resistance in the broiler production chain; File S2: Scoping review of factors affecting antimicrobial use and the spread of antimicrobial resistance in the broiler production chain.

## Abbreviations

The following abbreviations are used in this manuscript:

AMR	Antimicrobial resistance
AMU	Antimicrobial use
KAP	Knowledge, attitudes, and practices
WHO	World Health Organization
LMICs	Low-to-middle income countries
FAO	Food and Agricultural Organization
ESC	Extended-spectrum cephalosporin
ESBL/AmpC	Extended-spectrum beta-lactamases and AmpC beta-lactamases
ARGs	Antimicrobial resistance genes
PICO	Population, Intervention, Comparison, Outcome
BPS	Backyard production systems
FQr	Fluoroquinolone-resistant
MDR	Multidrug-resistant

## Appendix A. Search Strategy Details

**Table A1:** Search strategy.

Date	10 February 2023
Performed by	Zsuzsa Farkas
Databases	Scopus
Institution	University of Veterinary Medicine; Budapest, Hungary
Search string:	(antimicrobial resistance OR AMR AND poultry OR chicken OR gallus OR broiler AND factor OR driver) in TI (Title) in AB (Abstract or Author-Supplied Abstract) in KW (Author-Supplied Keywords)
Hits	544
Limits	Published since 2013

## Appendix B. Title and Abstract Relevance Screening Form

**Table A2:** Title and Abstract Relevance Screening Form.

Question	Options
Is the article written in English?	Yes → *Proceed* No → *Exclude*
Is the study about antimicrobial resistance?	Yes → *Proceed* No → *Exclude*
Is the study about poultry production chain?	Yes → *Proceed* No → *Exclude*

## Appendix C. Full Text Relevance Confirmation Form

**Table A3:** Full Text Relevance Confirmation Form.

Question	Options
Is publication type other than peer reviewed scientific article or primary research (e.g., review article, book chapter)?	Yes → *Exclude*
Is the publication about drivers and/or factors contributing to AMR in the poultry production chain?	Yes → *Proceed* No, it investigated the drivers and/or factors resulted in antibiotic usage → *Proceed* No → *Exclude*
Is the text in English?	Yes → *Proceed* No → *Exclude*

## Appendix D. Data/Information Extraction Form

**Table A4:** The Data/Information extraction form used during the study had the following fields.

Field	Attributes
Authors	
Title	
Published	
Driver (affecting more than 1 factor)	*free text*
Factor	*free text*
Bacterium	*free text*
Antibiotic or antibiotic group	*free text*
Disease type	*free text*
AMR extent	*free text*
Sample	*free text*
Region of the study conducted	values: Europe, North America, South America and Caribbean, Africa, Asia, Australia
Comment	*free text*
