# Supplementary material for: Scoping Review of Factors Affecting Antimicrobial Use and the Spread of Antimicrobial Resistance in the Poultry Production Chain
Source: Vet Sci. 2025 Sep 12;12(9):881. doi: 10.3390/vetsci12090881 (PMC12474243; doi:10.3390/vetsci12090881)
Supplement: Supplementary file 1 [file vetsci-12-00881-s001.zip › Supplementary Material_File_S1.pdf]

Supplementary Material File S1 A

Scoping review of factors affecting antimicrobial use and the spread of antimicrobial resistance in the broiler production chain

Zsuzsa Farkas[1,2], Orsolya Strang[1,2*], Andrea Zentai[1,2], Szilveszter Csorba[1,2], Máté Farkas[1,2], András Bittsánszky[1,2], András Tóth[1,2], Miklós Süth[1,3], Ákos Józwiak[1,2]

[1]Institute of Food Chain Science, Department of Digital Food Science, University of Veterinary Medicine Budapest, Budapest, Hungary

[2]National Laboratory of Infectious Animal Diseases, Antimicrobial Resistance, Veterinary Public Health and Food Chain Safety, University of Veterinary Medicine Budapest, Budapest, Hungary

[3]Institute of Food Chain Science, University of Veterinary Medicine Budapest, Budapest, Hungary

*** Correspondence:**
Orsolya Strang
strang.orsolya@univet.hu

Emergence of fluoroquinolone-resistant *Campylobacter jejuni* and *Campylobacter coli* among Australian chickens in the absence of fluoroquinolone use [1]

Abraham et al. investigated the antimicrobial resistance (AMR) and genomic characteristics of *Campylobacter jejuni* (n=108) and *C. coli* (n=96) from cecal samples of chickens at slaughter (n=200) in Australia. The majority of the *C. jejuni* (63%) and *C. coli* (86.5%) samples were susceptible to all antimicrobials. Fluoroquinolone resistance was detected among both *C. jejuni* (14.8%) and *C. coli* (5.2%), Multidrug resistance among strains of *C. jejuni* (0.9%) and *C. coli* (4.1%) was rare and fluoroquinolone resistance, when present, was never accompanied by resistance to any other agent. The detection of *Campylobacter* isolates exclusively resistant to fluoroquinolone was unexpected because fluoroquinolones are excluded from use in Australian livestock. The authors postulate that the fluoroquinolone-resistant strains detected in their study were introduced into the Australian chicken industry by mechanisms involving humans and/or wildlife (pests, wild birds).

Antimicrobials usage assessments in commercial poultry and local birds in North-central Nigeria: Associated pathways and factors for resistance emergence and spread [2]

Alhaji et al. conducted a questionnaire-based cross-sectional study on commercial poultry farmers and local bird flock keepers in Northcentral Nigeria. 384 (192 small-scale commercial poultry farms and 192 free-range local bird flocks) recruited poultry farmers/keepers participated. Respondents used antimicrobials for therapeutic, prophylactic, and growth promotion in birds. Small-scale commercial poultry farms frequently used antimicrobials without veterinarians' consultations but free-range local bird flocks rarely used antimicrobials. The improper antimicrobial dosage, nonenforcement of AMU (antimicrobial use) regulating laws, the weak financial status and also the low education and expertise of poultry owners as well as the husbandry management system (intensive and extensive) significantly influenced the misuse of antimicrobials in poultry. The study found very high practices of non compliance with AMs withdrawal periods, self administration of antimicrobials, giving only a single dose of AMs once on sick birds and AMs administration through drinking water. These results show an overall low knowledge level about proper AMU among both small-scale commercial poultry farmers and free-range local bird flocks keepers.

Risk factors and spatial distribution of extended spectrum β-lactamase-producing-*Escherichia coli* at retail poultry meat markets in Malaysia: A cross-sectional study [3]

Aliyu et al. collected 240 broiler meat (breast, wing, thigh and keel) samples and 80 samples from contact surfaces of weighing scales and cutting boards to investigate the occurrence of ESBL-EC at Malaysian wet markets. More than half of the meat samples contained ESBL-EC, with 65%, 52%, 50% and 47,5% occurrences in breast, wing, thigh and keel, respectively.

Univariable logistic regression indicated four factors with the likelihood of causing ESBL contamination, including stall sanitation, type of countertop, source of cleaning water and the type of cutting board/instrument. Based on the multivariable logistic regression model, only two factors were significant predictors of ESBL-EC, which were stall sanitation and type of countertop.

Stalls with poor sanitary environment were 6 times more at risk of ESBL-EC than those with good hygiene. Those with fair hygiene had twice the risk than those with good hygiene. The type of countertop was also signficantly associated, vendors using surfaces or countertops made of wooden materials had 8.1 times the risk of contamination, tiles countertops had 4.2 times the risk and plastic-sheet covered countertops had 3.7 times more risk of contamination than stainless-steel materials.

The authors also found that cutting-board/instrument material was associated with ESBL-EC. Compared to stainless steel cutting instrument, the univariate logistic model showed wooden cutting boards having 5,5 times the risk, and plastic cutting boards having twice the risk of contamination.

Source of cleaning water was also an important factor, as a majority of vendors used the same water to wash their hands and their utensils. Univariate logistic model found that meat vendors using the same water for cleaning purpose had 3 times the risk of cross-contamination than those using direct water from the tap.

Environmental surveillance of antimicrobial resistance in a rapidly developing catchment [4]

Ashwini et al. studied the prevalence of AMR in catchment in Western India. The environmental samples (soil, litter and water) were subjected to antibiotic susceptibility test to assess the resistance pattern in the bacterial species. Eighty isolates of over ten bacterial species were identified altogether from the soil, water, and litter samples. Out of 80, 46 isolates (57.5%) were from soil, 24 (30%) from water and 10 (12.5%) from the litter samples. Data shows that, the chicken litter harbours highest resistant bacterial strains and no resistance was observed in the bacteria isolated from the soil samples collected near the poultry farms. This can be attributed to biosafety protocols. In the surface water samples resistant bacterial isolates were also absent. The second highest resistance pattern was observed in the soil collected from the agricultural lands. The authors concluded that disinfection at the poultry farm level does not help in curbing the spread of AMR in the region. The use of litter as manure in agricultural soil leads to the spread of AMR in water and soil in places outlying from the poultry farm areas.

Prevalence and risk factors for multi-drug resistant *Escherichia coli* among poultry workers in the Federal Capital Territory, Abuja, Nigeria [5]

Aworh et al. studied the prevalence and risk factors for multi-drug resistant *E. coli* among poultry workers in Nigeria. Data was collected on socio-demographic and exposure factors from 122 healthy workers, and their stool samples were analysed for multidrug resistant *E. coli*.

Based on the final logistic regression model, absence of lavatory, the worker's history of diarrhea in the last three months and occupational exposure for more than 10 years were found to be risk factors for MDR.

Preharvest Environmental and Management Drivers of Multidrug Resistance in Major Bacterial Zoonotic Pathogens in Pastured Poultry Flocks [6]

Ayoola et al. analysed 1635 preharvest (feces and soil) samples collected from 42 pastured poultry flocks and 11 farms in the US. Evaluation of resistance of pastured poultry production systems representative of antibiotic-free environments provides

possibility to study the background antibiotic resistance (basal level) in the absence of selection pressure.

31 distinct farm variables and management practice variables associated with feces and soil were used together with 24 constituents/properties of poultry feces and pastured soil as additional input variables. Five different machine learning approaches were used to identify the critical farm management practices and environmental variables that drive multidrug resistance in *Salmonella* and *Listeria* in pastured broiler production systems that represents background resistance.

Acknowledging that the mechanisms behind the identified factors impacting MDR were not fully understood, based on the current literature, the authors provided potential explanations for a selection of drivers including magnesium, phosphorus, potassium content, carbon-nitrogen ratio, copper and chromium content of the soil, electrical conductivity and pH and manganese and sodium content of the soil.

Decreasing factors for MDR *Salmonella* and *Listeria* are the following:

- <300 ppm Mg in soil recommended to reduce MDR *Listeria* incidence
- ≤5000 ppm P in feces recommended for reduction of MDR *Listeria* incidence
- 7000–12,000 ppm K in feces recommended for reduction of MDR *Listeria* incidence
- >200 ppm K in soil recommended for reduction of MDR *Listeria* incidence
- >15 C:N in soil recommended for reduction of MDR *Listeria* incidence
- >18 ppm Cu in feces recommended for reduction of MDR *Listeria* incidence
- >3 ppm Cr in feces recommended for reduction of MDR *Listeria* incidence
- 6–15 years farming for soil recommended for reduction of MDR *Listeria* incidence
- ≥2000 ppm Mg in feces recommended for reduction of MDR *Salmonella* incidence
- ≤300 ppm Mg in soil recommended for reduction of MDR *Salmonella* incidence
- ≤150 ppm P in soil recommended for reduction of MDR *Salmonella* incidence
- ≤2000 microS/cm electrical conductivity of feces recommended for reduction of MDR *Salmonella* incidence
- ≤6.5 pH in feces recommended for reduction of MDR *Salmonella* incidence
- ≥70 ppm Mn in soil recommended for reduction of MDR *Salmonella* incidence
- <50 ppm Na in soil recommended for reduction of MDR *Salmonella* incidence

Modelling the impact of antimicrobial use and external introductions on commensal *E. coli* colistin resistance in small-scale chicken farms of the Mekong delta of Vietnam [7]

Bastard et al. studied the within-farm and outside-farm drivers of colistin resistance in small-scale chicken farms in Vietnam. Production cycles of nineteen farms were followed, when antimicrobial use data were recorded weekly and commensal *E. coli* samples were taken and tested for resistance at the beginning, middle and end of each production cycle. This enabled to account for the dynamics of the effect of AMU and AMR. Different models were fitted to the data using an expectation-maximization algorithm. Based on the best model, colistin resistance of *E. coli* was mostly affected by importations of resistance and to a lesser extent by the use of antimicrobials. The authors found, based on the model, that resistance was associated with the use of antimicrobials during the 1.73 weeks before the sample collection. They hypothesized that resistant *E. coli* strains could already be present at low levels or were introduced to the farm, and they were selected for by antimicrobial use. Use of antimicrobials in day-olds and carriage of colistin resistance from the hatchery were not influencing factors in this study. The authors reasoned that AMU may have a non-persistent effect on colistin resistance, which was further supported but not definitely confirmed by their modelling suggesting the effect of AMU lasting shorter than 2 weeks. In this study, most of the colistin resistance observed (17 of the 21 resistant samples) was actually not explained by antimicrobial use of the farm, but imported from outside, the nature of which could not be further determined. As the occurrence of importations were sometimes spatially clustered, the authors suggested a role of local environmental sources of resistance.

Characterisation of chicken farms in Vietnam: A typology of antimicrobial use among different production systems [8]

In their study Bâtie et al. conducted semi-structured interviews and on-farm questionnaires to collect socioeconomic, technical, biosecurity, health management, and antibiotic usage data on chicken farms in Vietnam. They identified three production systems (A, B, C) and three patterns of antibiotic usage (1, 2, 3). Group A raised in a closed, equipped building with biosecurity measures in an intensive setting. Farmers graduated from college or further higher education, had less than 5 years of experience and were under the age of 30. Group A was associated with group 1, which purchased antibiotics from companies. They used antibiotics according to the recommendations of the company for prevention and treatment. Group B mainly raised crossbreed chickens, a mix between a local and an exotic breed in a semi-confined system without biosecurity and was market-oriented, farmers have more than 15 years of experience and graduated from secondary school. Group B was

associated with group 3, which used antibiotics for treatment and prevention based on their own experience resulting overuse. In group C, farmers raised local breed chicken in semiconfined or in a free-range system in contact with other poultry and chickens were raised mainly for their own consumption or sold directly. Poultry production was not their main activity. Group C was associated with group 2, which used antibiotics for treatment according to the advice provided by the local drugstore or veterinarian.

Knowledge and use of antibiotics among low-income small-scale farmers of Peru [9]

Benavides et al. conducted questionnaires with small-scale farmers in Peru to estimate their use and knowledge of antibiotics. They found that the degree of knowledge was positively correlated with farmers' educational level, the knowledge score (KS) being significantly higher for farmers that finished primary and secondary education. KS also increased significantly among farmers that had higher monthly income than 500 Nuevos Soles. Furthermore, farmers having larger farms than 3 ha (farm area), knowing SENASA, the animal health authority, and knowing an antiparasitic drug had increased knowledge scores. The authors speculate, that the area of the farm could be associated with a higher economic value of the livestock activity. Knowledge, however, was not correlated with age, gender, main occupation, knowledge of a veterinarian or household size.

Potential misuse was also reported, including about one fifth of the respondents stopping treatment when clinical signs of the illness diminished, or infrequent use of antibiotics to treat parasites or animals not eating.

Genetic diversity and risk factor analysis of drug-resistant *Escherichia coli* recovered from broiler chicken farms [10]

In the work of Bhargavi et al. a total of 38 *Escherichia coli* isolates were recovered from 120 samples collected from various sources (poultry droppings, feed on feeders, feed from the stock, water from waterers, water from the main tank, and soil) of broiler chicken farms in India. Alarming multi-drug resistance (MDR) was observed (34/38) among the recovered isolates, wherein antibiotic-resistant genes (blaTEM, blaSHV, and tetA) were detected. In the present study, the highest antimicrobial resistance among the recovered *E. coli* isolates was observed towards ampicillin (31/38), followed by doxycycline (28/33), trimethoprim (27/38), cefpodoxime (26/38), ciprofloxacin (24/38), chloramphenicol (14/38), nitrofurantoin (8/38), gentamicin (6/38), ceftriaxone (5/38), and fosfomycin (1/38). There was a significant statistical association between the large flock size and ceftriaxone resistance, poor biosecurity practices, poor workers' hygiene, and gentamicin resistance. Moreover, the occurrence of the tetA gene was associated with poor biosecurity practices, poor workers' hygiene, and poor

disinfection practices, and the blaTEM gene was associated with poor workers' hygiene.

Drug use and antimicrobial resistance among *Escherichia coli* and *Enterococcus* spp. isolates from chicken and turkey flocks slaughtered in Quebec, Canada [11]

An observational study was conducted of chicken and turkey flocks slaughtered at federal processing plants in the province of Quebec, Canada. The objectives were to estimate prevalence of drug use at hatchery and on farm and to identify antimicrobial resistance (AMR) in cecal *Escherichia coli* and *Enterococcus* spp. isolates and factors associated with AMR. Eighty-two chicken flocks and 59 turkey flocks were sampled. At the hatchery, the most used antimicrobial was ceftiofur in chickens (76% of flocks) and spectinomycin in turkeys (42% of flocks). Virginiamycin was the antimicrobial most frequently added to the feed in both chicken and turkey flocks. At least 1 *E. coli* isolate resistant to third-generation cephalosporins was present in all chicken flocks and in a third of turkey flocks. Resistance to tetracycline, streptomycin, and sulfisoxazole was detected in 90% of flocks for *E. coli* isolates. Antimicrobial resistance (AMR) was observed to bacitracin, erythromycin, lincomycin, quinupristin-dalfopristin, and tetracycline in both chicken and turkey flocks for *Enterococcus* spp. isolates. No resistance to vancomycin was observed. The use of ceftiofur at hatchery was significantly associated with the proportion of ceftiofur-resistant *E. coli* isolates in chicken flocks. In turkey flocks, ceftiofur resistance was more frequent when turkeys were placed on litter previously used by chickens. This shows the importance of the environment in preserving and transmitting resistant bacteria. Associations between drug use and resistance were observed with tetracycline (turkey) in *E. coli* isolates and with bacitracin (chicken and turkey), gentamicin (turkey), and tylosin (chicken) in *Enterococcus* spp. isolates.

Zinc and Copper Reduce Conjugative Transfer of Resistance Plasmids from Extended-Spectrum Beta-Lactamase-Producing *Escherichia coli* [12]

Buberg et al. investigated the effect of excess levels of $ZnCl_2$ and $CuSO_4$ in the growth medium on the conjugative transfer of plasmids carrying the antibiotic resistance gene bla_{CMY-2} from extended-spectrum beta lactamase-producing *E. coli* derived from retail chicken meat. Copper and zinc have antimicrobial properties, and are routinely used as additives in animal feed. The authors hypothesized that transfer of plasmids would be promoted by these metals. As shown by other studies, bacteria could acquire resistance genes against drugs and metals with antibacterial properties on mobile genetic elements. In case more resistance genes are located on the same genetic element, it could lead to co-selection for metal and antibiotic resistance, and the selection for metal resistance could drive the spread of AMR. The authors found that conjugation of the IncK plasmid was reduced by more than 98% at all concentrations

of Zn and the two highest concentrations of Cu, while 0.01 mg/ml $CuSO_4$ resulted in a 90% reduction of conjugation. The reduction of IncI1 plasmid conjugation was more than 90% at the two highest Zn and Cu concentrations, while the lowest Zn and Cu concentrations resulted in 58% and 41% reductions, respectively. To understand the observed effect of Zn and Cu on conjugation, the authors performed a real-time transcriptional analysis which revealed a reduction in expression of conjugation-associated genes, nikB and traB. This finding suggested that concentration-dependent exposure of ESBL-producing *E. coli* to Zn and Cu reduce horizontal transfer of the bla_{CMY-2} resistance plasmid by reducing the expression of genes involved in the conjugation machinery.

Antimicrobial Resistant *Salmonella enterica* Typhimurium Colonizing Chickens: The Impact of Plasmids, Genotype, Bacterial Communities, and Antibiotic Administration on Resistance [13]

Bythwood et al. performed two studies; in the first study, chickens were administered *Salmonella* and commensals, including an *Escherichia coli* strain with a mobile, ceftiofur-resistance plasmid, in order to determine how antibiotic administration impacted resistance in *E. coli* and *Salmonella*. All antibiotics (ceftiofur, oxytetracycline, streptomycin) administered to chickens increased streptomycin resistance in *E. coli.* However, only ceftiofur administration increased resistance in *Salmonella* and specifically to extended-spectrum β-lactams and cephalosporins (ESBL). There was no significant increase in ESBL-resistant *Salmonella* in chickens administered a ceftiofur-resistance plasmid donor. In the second study, chickens were administered two different isolates of *S. enterica* Typhimurium and a chicken resistome to serve as a gene donor. Birds were subsequently administered chlortetracycline or streptomycin. Antimicrobial administration significantly altered aminoglycoside and tetracycline resistance in the *Enterobacteriaceae* population. However, there was no significant increase in antimicrobial resistant *Salmonella*. Administration of a chicken resistome had no significant impact on prevalence of resistance in *Enterobacteriaceae* populations, including *Salmonella*. Evident, from both studies, was that these treatments had minimal effect on increasing the prevalence of resistance in *Salmonella*, suggesting that other factors may be more important in dissemination of antimicrobial resistant *Salmonella* in chickens.

A cross-sectional study of the prevalence factors associated with fluoroquinolone resistant *Campylobacter jejuni* in broiler flocks in Canada [14]

Poultry are one of the main reservoirs of *Campylobacter*, a common cause of food-borne gastro-enteritis in people. *Campylobacter jejuni* accounts for the vast majority of infections. Ciprofloxacin is a broad-spectrum second-generation fluoroquinolone that is often the drug of choice for the treatment of *Campylobacter* gastro-enteritis in

humans. Caffrey et al. used data from a Canadian surveillance program to investigate prevalence factors associated with the isolation of fluoroquinolone resistant (FQr) *Campylobacter jejuni* from broiler faecal samples. The dataset consisted of 536 *Campylobacter jejuni* isolates recovered from 158 flocks. The type of bird used (Ross versus Cobb or mixed), the use of virginiamycin as a feed additive, the use of traps to control rodent populations in the barn, and the total number of birds in the barn were significant prevalence factors for increased FQr *C. jejuni* in a flock. On farms that used the Cobb line of birds, the odds of being resistant to fluoroquinolones was higher than on farms that used the Ross lineage of birds. The use of virginiamycin as a feed additive was also found to increase the odds of finding FQr *C. jejuni*. The use of traps to control rodent populations increased the odds of the flock being resistant to fluoroquinolones. In the current study the risk of isolating FQr *C. jejuni* increased with increasing flock size. Methods of disinfection of water lines between production cycles is important, with the use of chlorine being protective, and the use of hydrogen peroxide being a risk factor. The use of hot water to wash the barn between production cycles was also a significant protective factor.

Risk factors associated with the A2C resistance pattern among *E. coli* isolates from broiler flocks in Canada [15]

Caffrey et al. used data from the Canadian Integrated Program for Antimicrobial Resistance Surveillance (CIPARS) broiler farm surveillance program to investigate the risk factors for A2C, standing for simultaneous resistance to amoxicillin-clavulanic acid, ceftiofur and cefoxitin. The dataset consisted of 1478 *E. coli* isolates from 371 broiler flocks sampled between 2013-2015. All flocks were clustered within 21 hatcheries. In addition, questionnaires were used to collect data on farm operational risk factors, animal health and antimicrobial exposures. Risk factors identified for increased A2C resistant *Escherichia coli* isolates (A2C-REI) prevalence included the use of ceftiofur *in ovo* at the hatchery, and the use of H2O2 for treating water lines during the growing period. Based on the final model, the flocks that had 'TIO' (ceftiofur) had 1.91 times higher prevalence of A2C-REI, while usage of H2O2 to treat water lines during the production cycle was associated with a 1.52 times higher prevalence of A2C-REI in the flock. Factors associated with the decrease of A2C-REI prevalence included control of wild bird populations, use of avilamycin in feed and storage of manure on the farm. Controlling for wild birds on the farm was associated with a 0.64 times lower prevalence, while storing manure on the farm was associated with a 0.67 times lower prevalence of A2C-REI. Giving avilamycin with feed to the flock resulted in a 0.63 times lower prevalence of A2C-REI. The authors noted that ceftiofur was withdrawn in May 2014, which could have resulted in changes in the microbiota of the chicks, and could have led to the effect of avilamycin, the use of all of which occurred after the withdrawal of ceftiofur.

Antimicrobial usage in chicken production in the mekong delta of vietnam [16]

In the study of Carrique-Mas et al., data on antimicrobial usage on chicken farms were obtained from a survey carried out on 208 chicken farms sampled from three districts containing 40% poultry population in Tien Giang province, Mekong Delta, Vietnam. On meat farms and on farms run by a male farmer higher levels of usage were observed. All-in-all-out farming systems were associated with reduced levels of antimicrobial usage.

Trends and correlates of antimicrobial use in broiler and Turkey farms: A poultry company registry-based study in Italy [17]

Caucci et al. assessed antimicrobial use data applied in 5827 broiler and 1264 turkey grow-out cycles from 470 and 252 farms in Italy, respectively. The farms belonged to one of Italy's largest poultry companies, and data were retrieved for the years 2015-2017. In addition, they conducted a retrospective analysis to investigate the effects of geographical location, season, prescribing veterinarian and management and structural interventions by the company.

Seasonal and geographical variables played a major role in case of broilers, as a higher AMU was observed in Southern Italy, in winter/spring and in hilly/mountainous areas. The highest AMU was observed in spring. Despite the fact that chicks were housed in sheds with climate control, the authors speculated that this might be due to marked temperature shifts between day and night and lack of early intervention to adjust microclimatic parameters to avoid heat/cold stress. They also mentioned that in spring cycles, chicks were housed in winter, which is the least favourable time period. In spite of the more favourable conditions in hilly/mountainous areas, a higher AMU was also observed there, which the authors explained by a delayed level of farm renewal. It was also found that AMU increased in more densely populated areas.

Towards a bottom-up understanding of antimicrobial use and resistance on the farm: A knowledge, attitudes, and practices survey across livestock systems in five African countries [18]

Caudell et al. studied the knowledge, attitudes and practices regarding AMU and AMR across 887 farms in five African countries (layer farms in Ghana and Kenya, pastoralists keeping cattle, sheep and goats in Tanzania, and broiler farmers in Zambia and Zimbabwe), with the aim to identify the livelihood factors associated with these dimensions. They conducted focus group discussions and key informant interviews with stakeholders influencing antimicrobial use (farm owners, managers, workers, animal health professionals, owners and employees of agrovet shops and feed producers and distributors). It was found that farmers were generally aware of the negative associations between biosecurity and disease and AMR, but did not invest in

biosecurity due to economic considerations. Due to lack of resources, the farmers had limited access to proper animal health services, and agrovet employees were often the first sources of health advice sought by farmers. Antibiotics were usually sold without prescriptions, and observation of withdrawal periods was often limited. The authors found that experience in poultry keeping was positively associated with knowledge, with every additional year leading to a 0.4% increase in knowledge. Furthermore, every year increase in age was positively associated with a 0.2% increase in prudent attitudes. Regarding poultry, knowledge was higher in individuals who used more products with antimicrobials, kept records and had farm training by 0.2%, 10.6% and 18.4%, respectively. Those who kept records had 1.7% higher prudent practice scores. Farmers that received training also had more prudent attitudes (4.2%), but were associated with 3.0% decrease in prudent practice scores. The authors concluded that about 70% of respondents had sufficient knowledge and associated desirable attitudes, but this was not reflected in their practices.

Antibiotic usage practices and its drivers in commercial chicken production in Bangladesh [19]

Chowdhury et al. carried out a cross-sectional study to collect antibiotic usage information in commercial chicken farms in Bangladesh. Commercial chicken farms used diverse classes of antibiotics including tetracyclines, fluoroquinolones, macrolides, aminoglycosides, penicillins, and polymyxins. Doxycycline, oxytetracycline and ciprofloxacin were the most commonly reported antibiotics in broiler and layer chickens, whereas amoxicillin usage was more commonly reported in Sonali chickens. Sonali is a locally-produced cross-bred between Rhode Island Red male and Fayoumi female, reared for both meat and egg production. The cross-sectional survey revealed that the use of antibiotics in commercial chicken production was extensive in Bangladesh. Irrational and inappropriate use of antibiotics in commercial chicken production can contribute to the development of antimicrobial resistance. Most antibiotics were frequently administered for therapeutic and prophylactic purposes. Antibiotics were more commonly used in broiler and Sonali than in layer farms. The findings from this study emphasize that the improvement of chicken health through good farming practices can help to reduce antibiotic use and the consequential development of antimicrobial resistance. Regular monitoring of antibiotic usage, educating farmers, drug sellers and feed dealers about effective use of antibiotics, and restricting ease of access to antibiotics, may also be useful to reduce unnecessary use of antibiotics in commercial chicken production systems.

The costs, benefits and human behaviours for antimicrobial use in small commercial broiler chicken systems in Indonesia [20]

Coyne et al. explored the human behaviours and economic drivers related to antimicrobial use in small commercial broiler systems in Indonesia. As antimicrobials could be easily accessed through local channels and the relative cost of antimicrobials was low, the authors found that antimicrobial use was high, while it was difficult for farmers to access veterinary advice. Farmers routinely administered antimicrobials for productivity reasons. There were differences between contract farmers and independent farmers regarding the drivers of antimicrobial use. Contract farmers were more likely to use antimicrobials for prevention and based on advice from the contract company. They were also significantly more likely believing that using antimicrobials provided them with economic advantages and they had fewer concerns over AMR. Increased productivity was one of the most important reasons for using antimicrobials. There was also a positive association between performance and higher number of days antimicrobials were used. When factors were considered from the perspective of flock size and farm management type, the authors found association between increasing antimicrobial use and independent farmers with larger flocks.

Supply Chain and Delivery of Antimicrobial Drugs in Smallholder Livestock Production Systems in Uganda [21]

Dione et al. carried out stakeholder consultation workshops, key informant interviews with large-scale drug distributors and a knowledge, practices and awareness survey with actors of the veterinary drug supply chain in Uganda, focusing on the factors influencing the use of antimicrobials by livestock farmers. The authors identified low level of education of actors such as drug retailers and veterinary practitioners (about ninety percent did not receive specialized training in veterinary medicine), as potential driver of misuse of antibiotics. They also found that veterinary practitioners mainly serving small scale farmers were more knowledgeable regarding the drugs and AMR than their large-scale counterparts. The study further identified poor handling of drugs at purchase and administration practices, low enforcement of policy and regulations, and lack of awareness of stakeholders about policies, which may contribute to misuse of antibiotics. The authors pointed out the constant use of broad-spectrum antibiotics, which is caused by the low level of sophistication of the drug market and probably linked to the limited availability of diagnostic capacities.

Identification of risk factors associated with resistant escherichia coli isolates from poultry farms in the east coast of peninsular malaysia: A cross sectional study [22]

Elmi et al. investigated the prevalence, resistance patterns and associated risk factors of *E. coli* resistance in Malaysian poultry farms. They collected 371 samples (cloacal swabs = 259; faecal = 84; sewage = 14, tap water = 14) and responses to a questionnaire regarding management type, biosecurity, and history of disease from the sampled farms.

The source of water (eg. pump water, surface water) and the presence of a sewage system were identified as important risk factors for the presence of AMR in *E. coli* isolates in the study sites.

Multivariate regression analysis revealed that the risk factors including the source of samples (sewage samples) and farm size were leading drivers of *E. coli* antimicrobial resistance in the participating Malaysian states. They found that small scale poultry farms in the selected states were far more likely to carry AMR-resistant *E. coli* than medium and large scales farms.

The authors noted that the use of antimicrobials was very high (100%) in their study. Considering that 64.5% of participants reported receiving antimicrobials from regulated drug suppliers, it was assumed that many small holders may buy unregulated medicine from black markets and thus contribute to the development of AMR.

Factors Influencing Antibiotic Prescribing Behavior and Understanding of Antimicrobial Resistance Among Veterinarians in Assam, India [23]

The study of Eltholth et al. investigated factors influencing veterinarians' antibiotic prescribing behaviors and their understanding of antimicrobial resistance (AMR). The study used a telephone survey of 50 veterinarians conducted in five districts in Assam state, India. Ceftriaxone, enrofloxacin, and oxytetracycline were the antibiotics (ABX) most frequently prescribed, by 76, 68, and 54% of veterinarians, respectively. Less than two thirds (64%) were aware of the government ban for colistin and only 2% were aware of a national plan for AMR. The authors' results indicated that all respondents agreed that AMR is a major human and animal health problem caused by over use and/or improper AMU. This study sought to provide insights on prescribing behavior and opinions of veterinarians regarding AMU and AMR. The results concluded that they are unaware of the existing national and state level regulations, guidelines, and strategies for AMR.

Knowledge, attitude, and practices on antimicrobial use and antimicrobial resistance among commercial poultry farmers in Bangladesh [24]

Hassan et al. investigated the KAP (knowledge, attitudes, practices) status of broiler and layer farmers in Bangladesh. They observed that the level of knowledge had influence on the levels of attitudes and practices regarding AMU and AMR.

The factor score analysis revealed that the farmers' demographic and socioeconomic variables were significant factors influencing their KAP. Based on adjusted logistic regression analysis, they showed that older farmers with 9–12 years of farming

experience and graduate-level education, engaging in medium-sized layer farming, were more likely to have correct KAP.

Specifically, farmers between 36 and 40 years of age had 0.13 times, those between 41 and 45 years of age had 0.17 times, and those who were 46 or older had 0.23 times the correct knowledge compared to those between 18-35 years. Regarding attitudes, the farmers' aged between 41 and 45 years had 0.11 times, and those older than 46 years of age had 3.21 times favorable attitudes compared to the farmers aged 18–35 years. Regarding practice, farmers between 36 and 40 years of age were good performers.

Considering experience in poultry farming, the farmers who had experience between 5 and 8 years had 7.13 times, between 9 and 12 years had 11.54 times, and those with more than 13 years had 7.27 times the correct knowledge compared to those having less than 4 years of experience. Regarding practice, those with experience between 5 and 8 years had 7.23 times, 9–12 years had 4.63 times, and 13 or more years had 3.76 times more good practices compared to those farmers with 0–4 years of experience.

Considering education, the farmers who completed graduation had 2.96 times the "correct" knowledge on AMU and AMR than farmers who received education up to the 12th grade. Furthermore, they had 2.49 times more favorable attitudes and performed 2.97 times more good practices compared to the latter.

The size of the farms was also an associated factor. The farmers who owned larger farms had 3.95 times the correct knowledge, 6.96 times more favorable attitudes and 0.36 times more good practices compared to farms with small sizes.

The analysis further revealed that the farmers in high-income groups had 14.30 times more favorable attitudes and performed 0.35 times more good practices than the middle-income group.

The authors found that the farmers who raised layer poultry had 2.01 times the "correct" knowledge compared to broiler farmers. They reasoned that layer farming needs a more extended period to reach the production level, therefore farmers invest more money for the sake of more benefits. Broiler farming, on the other hand requires less time and less investments, and farmers get back their investment and profits within a shorter period.

Notably, the authors also found that one-third of the farmers did not contact registered veterinarians regarding antimicrobials, but depended on others (eg. feed sellers and drug sellers) and themselves instead.

Reduction in antimicrobial use and resistance to *Salmonella*, *Campylobacter*, and *Escherichia coli* in broiler chickens, Canada, 2013-2019 [25]

Huber et al. monitored trends in antimicrobial use and AMR in broiler chicken on data obtained from the Canadian Integrated Program for Antimicrobial Resistance

Surveillance (CIPARS) during 2013-2019. They aimed to quantify the effect of antimicrobial use and farm management factors on AMR by using LASSO regression (a regression method to enhance the prediction accuracy and interpretability of the resulting statistical model) and generalized mixed-effect models. Consistent with the implementation of the Chicken Farmers of Canada's AMU Reduction Initiative, the authors observed a reduction for most antimicrobial classes in both AMU and AMR during the studied time period. However, increased resistance to streptomycin and tetracycline in *Salmonella* isolates, an increase in resistance to gentamicin and nalidixic acid in *E. coli* isolates, and an increase in resistance to nalidixic acid in *Campylobacter* were found. The authors speculated that the elimination of hatchery-level use and reduced preventive AMU through feed could potentially lead to an increased frequency of infectious diseases and an increased need for AMU through water for treatment. There was no difference in resistance rates between farms that used ideal methods for disinfection and those that didn't, and between antimicrobial-free and conventional farms. However, prevalence of *Salmonella* serotypes of higher public health importance was lower on antimicrobial-free farms and on those that used ideal methods for disinfection. The authors also studied the effects of the route of administration. They found that injection of the drugs *in ovo* or subcutaneously at hatcheries were significantly associated with resistance.

Identification of *Escherichia coli* from broiler chickens in Jordan, their antimicrobial resistance, gene characterization and the associated risk factors [26]

Ibrahim et al. studied the serotypes and resistance of avian pathogenic *E. coli* (APEC) isolated from the visceral organs of sick broiler chickens in Jordan. A questionnaire regarding potential risk factors was also filled by the farms providing the samples. One of the main risk factors associated with the presence of multidrug resistant (MDR) *E. coli* were the usage of water from artesian wells by the farms. They found that poultry drinking water increased the incidence of having MDR *E. coli* compared to farms supplied by the municipalities' drinking water.

They also found that farms located in close proximity to other poultry farms were at higher risk of contracting MDR *E. coli*. They stated therefore that farmers should pay attention to wind directions in their area as serious pathogens are transmitted by air, which was positively correlated to farm density. Personal movement, vehicles and instruments were also considered as potential vectors for transmission of pathogens.

Knowledge, attitudes and practices regarding antimicrobial usage, spread and resistance emergence in commercial poultry farms of Rajshahi district in Bangladesh [27]

Islam et al. assessed the knowledge, attitude and practices regarding AMU and beliefs in factors affecting AMR of poultry farmers in Bangladesh. Educated farmers had a significantly higher mean score of better attitudes. The majority of the farmers stopped using antimicrobials before completing the full dose, and half of surveyed participants did not keep records of the antimicrobials used.

Antimicrobial resistance of *Escherichia coli* isolated in newly-hatched chickens and effect of amoxicillin treatment during their growth [28]

In their study Jiménez Belenguer et al. investigated the resistance patterns of *E. coli* isolated from newly-hatched chicken and the effect of amoxicillin treatment during their growth in Spain. In their work the researchers have demonstrated the existence of a high percentage of resistant *E. coli* strains in one old day chickens, not exposed previously to any antibiotic, which strongly suggest the possibility of vertical transmission from parent flocks. On the other hand, influence of amoxicillin treatments in increasing resistances to beta-lactams, aminoglycosides and chloramphenicol has been shown.

Epidemiological Dynamics of Extended-Spectrum β-Lactamaseor AmpC β-Lactamase-Producing *Escherichia coli* Screened in Apparently Healthy Chickens in Uganda [29]

Kakooza et al. investigated the prevalence and factors linked to the fecal carriage of extended-spectrum β-lactamase- (ESBL-) or AmpC producing *Escherichia coli* (ESBL-/AmpC-EC) in commercial chickens in Uganda. Cloacal swabs were sampled from 400 chickens reared on 20 farms. A total of 70 out of 400 samples (17.5%) tested positive for ESBL-/ AmpC-EC. Univariable screening hypothesized that carriage was probably influenced by a type of commercial chicken, geographical location, age group, flock size, and housing system. Modeling exposed that broiler birds were at a higher risk of being ESBL-/ AmpC-EC carriers than layer and dualpurpose birds. Birds from Wakiso Town Council and flocks of 700–1200 birds were also at a higher risk of harboring ESBL-/AmpC-EC. The risk of having ESBL-/AmpC-EC was more pronounced amongst birds less than 1 month old. Wakiso Town Council had more human settlements compared to the other subcounties. Samples from the deep litter system had a higher ESBL-/AmpC-EC prevalence compared to those from the battery cage system. This study confirmed that Ugandan poultry can be a potential reservoir of ESBL-/AmpC-EC shed into the environment through fecal matter.

Knowledge, Attitude, and Practices on Antimicrobial Use and Antimicrobial Resistance among Poultry Drug and Feed Sellers in Bangladesh [30]

Kalam et al. investigated the KAP of poultry drug and feed sellers in Bangladesh using a questionnaire. The factor score analysis revealed that years of experience, level of education, and training on the drug were the significant factors impacting the KAP of AMU and AMR. The correlation was fair between knowledge–attitudes, knowledge–practices, and attitudes–practices.

Based on the adjusted logistic regression analysis it was shown that drug sellers who completed education up to 12th grade had 4.88 times the odds of having 'correct' knowledge compared to respondents who completed graduation, and those who received training had 5.22 times the odds of having 'correct' knowledge. It was also observed that the drug sellers of the age group 31–35 and 36–40 years and who completed 12th grade had good attitudes compared to those above 40 years. The analysis further determined that drug sellers belonging to the age category 18–25 and 26–30 years had better practices. It was also shown that respondents who completed education up to 12th grade had 3.68 times the odds of performing better practices compared to their graduate counterparts.

Knowledge, Attitudes, and Common Practices of Livestock and Poultry Veterinary Practitioners Regarding the AMU and AMR in Bangladesh [31]

Kalam et al. 2022 assessed factors associated with veterinarians' drug prescription behaviour and KAP on AMU and AMR in Bangladesh. According to their results, veterinarians rarely used culture and susceptibility tests when prescribing antimicrobials, but were rather influenced by the farmer's economic condition. 36% of respondents used antimicrobials due to the demand of farmers regularly of frequently. Antibiotics were used for prophylaxis regularly (14.5%), frequently (37%), or rarely (35%).

Factor score analysis revealed that age, level of education, years of experience in practice and previous training on AMU and AMR were significant factors affecting knowledge. Also, current workplace was positively associated with knowledge, as veterinarians working in government hospitals were 2.09 times more likely to have a higher knowledge score than those working in medicine/feed companies. Moreover, those respondents who received training on AMU and AMR were 1.92 times more likely to have higher knowledge.

Regarding practices, gender and level of education were influencing factors, and training was positively associated with prescription practices. The respondents who received training were 0.76 times more likely to have good practices.

It was also shown that age and previous training were positively associated with favorable attitudes. Veterinarians of 36–40 years were 0.24 times more likely to have favorable attitudes than those between 18–25 years of age, and those who received training were 2.09 times more likely to have favorable attitudes.

Veterinarians usually prescribed antimicrobials without visiting the farms. This was partly explained by the scarcity of available services in those areas. High transport costs, poor transport facilities and poor health condition of the animal made transport of sick animals hard for farmers in remote areas. Unprofessional practitioners partly covered missing veterinary services in certain hard-to-reach areas, which may have resulted in a worsening of the AMR situation in the country.

Fijian Farmers' Attitude and Knowledge Towards Antimicrobial Use and Antimicrobial Resistance in Livestock Production Systems–A Qualitative Study [32]

In this study semi-structured qualitative interviews were conducted in Fiji. The participants comprised livestock farmers and managers who raised livestock, managed, and directly administered antimicrobials to livestock in their farms. A total of 19 livestock farmers and managers participated in the interviews from the cattle and poultry production systems (dairy, beef, broiler, and layer) was targeted to generate indepth, rich accounts and descriptions on AMU and AMR. Most participants lacked general understanding and awareness on AMU and AMR and its mechanism of action. Most of the participants did not differentiate between antimicrobials and other types of medicine. Most participants inherited their livestock farms from their ancestors, and livestock production was their primary source of income. Hence, the sustainability of livestock production was essential to their livelihoods. Mitigating risks on-farm was crucial, and the use of antimicrobials was perceived to be the first line of defense. Many participants highlighted that there was a significant shortage of medicines in Fiji and medicine shortage resulted in livestock farmers hoarding medicine, resulting in self-prescribing. Most participants did not know that there were livestock associations that they could join to share experiences, access training, and learn about livestock management. Livestock farmers relied on foreign farmers and veterinarians for information and guidance about livestock production, management, and medicine use. They lacked trust in knowledge and advice provided by the local veterinarian and para-veterinarians.

Assessment of drivers of antimicrobial use and resistance in poultry and domestic pig farming in the Msimbazi river basin in Tanzania [33]

In their work Kimera et al. assessed the drivers of antimicrobial use and resistance in poultry and domestic pig farming and the environment in Tanzania. Questionnaires, in-depth interviews, and focus group discussions (FGDs) were used to collect information regarding demographic characteristics, knowledge, practices, attitudes, and perceptions of the drivers of antimicrobial use and resistance in animal farming and the environment. 113 farmers responded to the questionnaire and the majority (92%, n = 104) were not aware of AMR. The results showed very high usage (87.6%) of veterinary antimicrobials in poultry and domestic pig farming mostly for prophylaxis

rather than treatment. Farming experience has a significant relationship with the prudent use of antimicrobials and the proper sourcing of the antimicrobials. The level of education was significantly associated with the frequency of using antimicrobials and performing group treatment. Stocking of antimicrobials was common among farmers. There was a significant relationship between farming experience and the method used for disease diagnosis, access to veterinary services, storage of veterinary antimicrobials and group treatment. The level of education was significantly related to the possibility of reducing antimicrobial use. The misuse of antimicrobials to shorten the period of farming for financial gain was also common. The use of human antimicrobials in animals, inadequate veterinary extension officers, inadequate knowledge on infection prevention and control of animal diseases were significantly associated with the level of education. The main factors associated with the spread of AMR in the environment according to the respondents were the disposal of solid wastes from the household, agricultural activities that involve use of animal manure, uncontrolled disposal of human and veterinary drugs and the use of river water for irrigation. Farmers were making treatment decisions based on the presentation of clinical signs.

Comparison of Antibiotic Resistance and Virulence Factors among *Escherichia coli* Isolated from Conventional and Free-Range Poultry [34]

The aim of the study of Koga et al. was to analyze the profile of antimicrobial resistance and virulence factors of *E. coli* isolates from chicken carcasses obtained from conventional and free-range poultry farming systems in Brazil. A total of 156 *E. coli* strains were isolated and characterized for genes encoding virulence factors. Antimicrobial susceptibility testing was performed for 15 antimicrobials (ciprofloxacin, ampicillin, gentamicin, norfloxacin, enrofloxacin, cefazolin, cefotaxime, cefoxitin, ceftazidime, tetracycline, nalidixic acid, chloramphenicol, nitrofurantoin, trimethoprim-sulfamethoxazole, and amoxicillin-clavulanic acid), and strains were confirmed as extended spectrum of β-lactamases- (ESBLs-) producing *E. coli.* The absence or restricted use of antimicrobials in free-range poultry production may be contributing to the lower frequency of bacterial virulence factors and resistance to antimicrobials, leading to a lower risk of their transmission to humans. Strains from conventionally raised chickens had a higher frequency of antimicrobial resistance for all antibiotics tested and also exhibited genes encoding ESBL and AmpC, unlike free-range poultry isolates, which did not.

Antimicrobial Resistance in *E. coli* Isolated from Chicken Cecum Samples and Factors Contributing to Antimicrobial Resistance in Nepal [35]

In this study from Nepal chicken cecal samples were collected to isolate *Escherichia coli*. Of the 190 chicken cecum samples collected, 170 (89%) were subjected to culture and

drug sensitivity testing, of which *E. coli* was isolated from 159 (94%) samples. Of the 159 isolates, 113 (71%) had resistance to ≥3 antimicrobial class. Resistance to tetracycline (86%) and ciprofloxacin (66%) were most prevalent. Overuse of antimicrobials, easy availability of antimicrobials, and lack of awareness among farmers about AMR were major issues contributing to AMR. Among the 159 cecal samples, six samples had two isolates of *E. coli*. Of the 165 isolates, more than 50% isolates were resistant to tetracycline (86%), ciprofloxacin (66.1%), ampicillin (60.0%), and cotrimoxazole (50.9%). Only 12 (7.3%) out of 165 isolates were resistant to cefotaxime. Three major drivers were identified for AMR, and the drivers were further classified into categories as follows:

1. Overuse of Antimicrobials
    a. No guidelines regarding the sale of antimicrobials
    b. Lack of training among veterinary personnel
    c. Irrational sales of antimicrobials
2. Issues Related to Livestock Farmers
    a. Poor financial status
    b. Carelessness among the livestock farmers
3. Availability of Falsified/Substandard Drugs

Impact of colistin sulfate treatment of broilers on the presence of resistant bacteria and resistance genes in stored or composted manure [36]

Le Devendec et al. aimed to study the impact of colistin administration and of manure management on the presence of colistin resistant *E. coli*, *K. pneumoniae* and *P. aeruginosa*, and the prevalence of antimicrobial resistance genes in broiler feces and in manure.

The detection of a selection of AMR genes indicated that most of them were already present in the chickens' guts on entrance to the premises, which could be explained by vertical transmission of resistant bacteria from the breeders or by contamination at the hatchery or during transport.

The experiment revealed that colistin administration to chickens had no apparent impact on the antimicrobial resistance of the dominant *Enterobacteriaceae* and *P. aeruginosa* populations in the gut. Due to composting, a limited decrease in resistance genes was observed. Furthermore, the results of plasmid capture assays showed that plasmids were still present in manure after three or six weeks, which were transferred by conjugation. It was shown that plasmids carrying AMR genes could still be transferred even after 6 weeks of composting or storage. This finding suggested that composting was insufficient to completely eliminate the risk of dissemination of AMR through chicken manure.

Animal husbandry practices and perceptions of zoonotic infectious disease risks among livestock keepers in a rural parish of Quito, Ecuador [37]

Lowenstein et al. conducted twenty semistructured, in-depth interviews with small-scale livestock producers in a semirural parish of Quito, Ecuador to explore the livestock-raising practices, including animal health-care practices and use of antimicrobials, family members' interactions with livestock and other animals, and perceptions of health risk associated with these practices and activities. Many respondents had lack of familiarity with the term of antibiotics or confused antibiotic drugs with vitamins, vaccines, and other medications. However, the lack of familiarity with the term and the risks associated with antibiotic misuse is revealing.

Associations between antimicrobial use and the faecal resistome on broiler farms from nine European countries [38]

The aim of this study was to determine the relationship between the broiler faecal resistome and farm- and flock-level usage of antimicrobials and farm biosecurity status in nine European countries. In this study Luiken et al. quantified resistance using the resistome of pooled faecal flock samples obtained by metagenomic analysis and related this to AMU data of the broiler flocks and farms from different countries. The results confirm the hypothesis that higher antimicrobial exposure at flock or farm level is associated with more AMR. This study applied metagenomics to establish associations between AMU and the resistome on European broiler farms. Clearly positive associations between corresponding AMU and resistance genes were observed. Significant results were shown for both flock-level and farm-level usage, highlighting that both actual and historic use can contribute to AMR presence. The data did not support associations with ARGs and non-corresponding AMU or biosecurity status of the farm. However, they showed that the faecal microbiome harbours many resistance genes in the absence of current AMU.

Determinants for antimicrobial resistance genes in farm dust on 333 poultry and pig farms in nine European countries [39]

In this study absolute ARG levels, representing the levels people and animals might be exposed to, and relative abundances of ARGs, representing the levels in the bacterial population (normalized over 16S), were quantified in airborne farm dust using qPCR by Luiken et al. Four ARGs were determined in 947 freshly settled farm dust samples, captured with electrostatic dustfall collectors (EDCs), from 174 poultry (broiler) and 159 pig farms across nine European countries (Belgium, Bulgaria, Denmark, France, Germany, Italy, the Netherlands, Poland and Spain). Associations with fecal ARG levels, antimicrobial use (AMU) and farm and animal related parameters were determined. Results show similar relative abundances in farm dust

as in feces and a significant positive association between the two reservoirs. AMU in pigs was positively associated with ARG abundances in dust from the same stable. Higher biosecurity standards were associated with lower relative ARG abundances in poultry and higher relative ARG abundances in pigs. Lower absolute ARG levels in dust were driven by, among others, summer season and certain bedding materials (shredded straw) for poultry, and lower animal density and summer season for pigs.

Antimicrobial use in household, semi-industrialized, and industrialized pig and poultry farms in Viet Nam [40]

A cross-sectional study was conducted by Luu et al. in northern, central and southern Vietnam. The study population was poultry (n=540) and pig farmers (n=540) in three categories based on their farm size: household farms, semi-industrial farms, and industrial farms. The objective of this study was to determine farmers rationale behind AMU on their farms and their usage patterns. On chicken farms, 87.9% reported that they use antimicrobials in the production. The results of the survey showed that the three main purposes of AMU were treatment of sick animals, disease prevention and weight gain. There were many identified drivers of AMU such as lack of access to veterinary services, easy access to cheap over-the-counter antimicrobials, and insufficient farm biosecurity. Farm owners with a high school education were significantly more likely to ask for advice before using antimicrobials and to comply with withdrawal times before slaughtering. The proportion of farm owners with a college or university degree who performed diagnostic tests before using antimicrobials was significantly higher. Farm owners with a high school education and farm owners with a college or university degree were significantly more likely to use a combination of more than one antimicrobials in a treatment therapy. Proportion of industrial poultry farms using antimicrobials was significantly higher than household farms and consultation rates before using antimicrobials in industrial farm was significantly higher than household farms. Chicken farmers which used diagnostic tests before using antimicrobials was significantly more likely to be semi-industrial and industrial farms and compliance with the manufacturer's recommended dosage on semi-industrial farms and industrial farms was significantly higher compared to household farms. Compliance on industrial chicken farms with the withdrawal time was significantly higher compared to households.

Antimicrobial resistance of commensal *Enterococcus faecalis* and *Enterococcus faecium* from food-producing animals in Russia [41]

Although *Enterococcus faecalis* and *Enterococcus faecium* are common members of human and animal gut microbiota, their resistance to different antimicrobials makes them important pathogens. Makarov et al. isolated *E. faecalis* and *E. faecium* from chickens, cattle, pigs, turkeys, sheep and ducks from different regions of Russia.

Isolates were tested for resistance to 12 antimicrobials from 11 classes. Among antimicrobials, resistance to bacitracin and virginiamycin was 88-100% in nearly all cases. High levels of clinical resistance were found for both bacteria species: Rifampicin (44-84%) from all animals, tetracycline (45-100%) from poultry and pigs, and erythromycin (60-100%), ciprofloxacin (23-100%), and trimethoprim-sulfamethoxazole (33-53%) from chickens, turkeys, and pigs. No vancomycin-resistant isolates were found. Antimicrobial application may lead to the development of resistance in the enterococci population. Further research is needed to investigate genes of resistance and factors determining the resistance prevalence.

Epidemiology and antimicrobial resistance of *Escherichia coli* in broiler chickens, farmworkers, and farm sewage in Bangladesh [42]

The present study was carried out to determine the prevalence and AMR profile of *Escherichia coli* isolated from broiler chickens, the environment, and farmworkers in Bangladesh. This study also aimed to identify the risk factors associated with multidrugresistant (MDR) *E. coli* infection in broiler chickens. In addition, the presence of carbapenem resistance gene (NDM-1) was assessed. A total of 114 *E. coli* isolates, recovered from 150 samples (cloacal swabs = 50, farm sewage = 50, and hand washed water of farmworkers = 50) collected from 50 broiler farms. The overall prevalence of *E. coli* was 76% and the highest prevalence (86%) was observed in cloacal swab samples. While exploring the risk factors for MDR *E. coli* infection in broiler chickens, three potential risk factors namely 'winter season', 'absence of specific shoes for staff', and 'use of antibiotics without prescription of veterinarians' were identified. In the antimicrobial susceptibility study, 10 antibiotics of six different classes were used. Four (meropenem, ceftriaxone, colistin, and ciprofloxacin) of the antibiotics tested in this study are classified by the World Health Organization as extremely important antibiotics in human medicine, and the other six (levofloxacin, ceftazidime, cefotaxime, amoxyclav, doxycycline, and imipenem) are classified as highly important antibiotics. The *E. coli* isolates in this study exhibited resistance to all classes of the tested antibiotics. High resistance was observed against fluoroquinolones (84.2%), followed by cephalosporin (80%) and tetracycline (78.1%). Among fluoroquinolones, 81.6% and 70.2% of isolates showed resistance to levofloxacin and ciprofloxacin, respectively. None of the isolates were positive for the NDM-1 gene. In this study, about 76% of *E. coli* isolates exhibited multidrug resistance, of which 78.8% isolates from hand washed water, 76.3% isolates from farm sewage, and 74.4% form cloacal swab.

A Qualitative Study of Antibiotic Use Practices in Intensive Small-Scale Farming in Urban and Peri-Urban Blantyre, Malawi: Implications for Antimicrobial Resistance [43]

In their study Mankhomwa et al. investigated the household antibiotic use practices in food animals in urban- and peri-urban Blantyre, Malawi. The qualitative research methods focused on households that kept scavenging animals and those engaged in small-scale intensive farming of food animals. One of the most important research findings was the heavy dependence smallscale farmers had on antibiotics. Farmers and occasionally shop attendants were unaware of antibiotics in food and vitamin mixes. Because of limited available space, animals often are kept in cramped space within the farmer home. The cost of veterinary advice and antibiotics often resulted underdosed or slaughtered sick animal. The farmers unrestricted access to antibiotics, their livelihood precarity and unequal market condition contribute to antibiotic usage.

Antibiotic prescription patterns and non-clinical factors influencing antibiotic use by Ecuadorian veterinarians working on cattle and poultry farms: A cross-sectional study [44]

A questionnaire was administered cross-sectionally to Ecuadorian veterinarians working on cattle and poultry farms by Martínez et al. to explore the antibiotic prescription patterns and nonclinical factors (e.g., attitudes and perceptions) influencing antibiotic use, and to identify strategies to reduce antibiotic use. Cattle and poultry veterinarians perceived similar barriers to increasing antibiotic stewardship including: poor biosecurity measures, animal confinement, low feed quality, farmers do not call a veterinarian due to economic reasons, stopping antibiotic treatment, storing antibiotics on farms, buying antibiotics in veterinary supply stores, and sales agents' roles as nonprofessional prescribers of antibiotics.

Presence of antimicrobial resistance in coliform bacteria from hatching broiler eggs with emphasis on ESBL/AmpC-producing bacteria [45]

Mezhoud et al. analysed 186 broiler hatching eggs from eleven broiler breeder farms, for the presence of ESBL/AmpC producer *Enterobacteriaceae*, and found that broiler hatching eggs could be carriers of resistant bacteria. They found *E. coli* and *Enterobacter cloacae* in the eggshells collected in 10 out of 11 farms. An important finding was that *Enterobacteriaceae* were mainly isolated from crushed eggshells even after extensive decontamination procedures. This suggests that bacteria may be unreachable by the disinfection agents in the egg shell pores and resistant bacteria that survive decontamination may colonize 1-day-old chicks and spread in the hatchery.

Risk factors for occurrence of cephalosporin-resistant *Escherichia coli* in Norwegian broiler flocks [46]

Mo et al. studied 182 broiler flocks in 27 farms to determine risk factors for the occurrence of cephalosporin-resistant *E. coli* in Norwegian broiler flocks. Samples were collected from broiler and parent flocks, and a questionnaire including multiple choice questions on potential risk factors was conducted. Risk factors were estimated with a multivariable generalized linear model. Disinfection of floor between production cycles reduced the odds of a positive status, while a positive status of the previous flock in the same house, having transport personnel entering the room where the broilers were raised and having three or more parent flocks supplying the broiler flock with day-old chickens increased the odds of a positive status for cephalosporin-resistant *E. coli*.

The most significant risk factor was the positive status of the previous flock. In case of previous positive status, the odds of the next flock being positive was approximately 13 times higher. The odds of a positive status also increased when three or more parent flocks supplied day-old chickens for a specific broiler flock, which may be explained by an increased probability of at least one of the parent flocks being positive. However, the authors found no apparent association between the status of the supplying parent flocks and the status of broiler flocks, and a limited occurrence of cephalosporin resistant *E. coli* was found in parent flocks. The authors also reasoned that several parent flocks may have a low level of cephalosporin-resistant *E. coli* circulating in parent flocks, which could not be detected by the analytical tests used. However, in case several parent flocks with low levels of resistance supplied a broiler flock, the „burden" of cephalosporin-resistant *E. coli* could accumulate and the result for the broiler flock become positive.

<u>Knowledge, attitudes, practices and risk perception of rural poultry farmers in Cameroon to antimicrobial use and resistance</u> [47]

Moffo et al. studied the knowledge, attitudes, practices and perception of poultry farmers regarding AMU and AMR in Cameroon. They found that gender, experience in poultry farming and stocking density were negatively associated with practice of antimicrobial use. A strong positive association was observed between educational level and KAPP score and between biosecurity measures and AMU.

Locality, age (negatively associated), training in poultry and stocking density were the major factors affecting knowledge score on AMU and locality, age and ecuational level were factors significantly influencing knowledge score on AMR. Furthermore, almost 32% of respondents were not adequately consulting with animal care personnel, which is associated with their lower knowledge score.

As inadequate practice, the study mentions using antimicrobials as painkiller and for treatment of viral diseases, furthermore, giving them to one-day old-chicks for preventive purposes and applying antimicrobials with diuretics or tetracycline with minerals. These latter practices lead to sub-lethal concentration of the drug which can

induce stress in the bacteria leading to mutation and might also result in a transient decrease in antimicrobial susceptibility.

The authors found that knowledge was strongly associated with practices, attitude and risk perception of AMR.

Poultry litter contamination by *Escherichia coli* resistant to critically important antimicrobials for human and animal use and risk for public health in Cameroon [48]

Moffo et al. investigated the antimicrobial resistance of *E. coli* isolated from poultry litter in Cameroon. Litter samples were collected and stored at room temperature for two months to represent the delay period observed by most farmers before the application of litter as manure. The assumption of the authors was that storage of litter in plastic bags for at least two months may help to reduce potential pathogens. Questionnaires were also filled by farmers on their socio-demographic and farm characteristics. Resistance prevalence of *E. coli* obtained from litter was 58.4%. Multivariable logistic regression analysis showed that multidrug resistance of *E. coli* was associated with lack of training and less experience in poultry farming (young farmers with at least 5 years of experience in poultry farming), and a high frequency of digestive tract disease. The majority of respondents used antimicrobials for both preventive and curative purposes, and one fifth of them used the drugs as feed additives.

The authors observed a lower isolation frequency compared to previous studies on fresh broiler litter, which, according to their view, could be the result of the storage of the samples. However, the observed level was still high indicating that litter could serve as a potential reservoir of resistance.

The authors also observed some unexpected resistance to certain drugs which were not used in animal production in the country, possibly the contribution of factors other than antimicrobial use, indicative of co-resistance.

The results of the study indicated that litter was an important source of resistant genes and AMR bacteria which could be transported even weeks after their extraction from farms.

The dynamic of antibiotic resistance in commensal *Escherichia coli* throughout the growing period in broiler chickens: fast-growing vs. slow-growing breeds [49]

Montoro-Dasi et al. investigated the AMR dynamic during the growing period in two genetic poultry breeds, a fast-, and a slow-growing breed. 576 broilers from the same hatchery were housed in two identical poultry houses (replicas A and B). Half of the animals were fast- (Ross), and half were slow-growing breeds (Hubbard), providing a number of 144 for both groups in each of the poultry houses. No antibiotics were

administered to the animals, and they were sampled for *E. coli* on the day of arrival to the poultry house (day-old chicks), at mid-period and at slaughter day. The authors found that at the onset of the growing cycle, 100% and 63.6% of the *E. coli* isolates from fast-growing and slow-growing breeds were antibiotic resistant. Furthermore, 75% of the antibiotic resistant strains in fast-growing chicks were multidrug resistant, and 0% in the slow-growing breed were MDR. However, by the end of the growing period, these differences disappeared, as the fast-growing and slow-growing strains reached AMR rates of 95.6% and 96.2%, respectively. As regards prevalence of MDR, 83.7% and 84.3% of antibiotic resistant strains showed an MDR pattern by the end of the cycle in fast-growing and slow-growing breeds, respectively. These results suggested the possibility of vertical transmission from the breeders to day-old chicks, as despite no administration of antibiotics, the same high level of AMR rates were observed in both breeds by the end of the cycle. In addition, the disappearance of the initially observed significant differences between the breeds by the end of the cycle suggested a dissemination spread through the environment between flocks.

Cross-Sectional Survey of Prophylactic and Metaphylactic Antimicrobial Use in Layer Poultry Farming in Cameroon: A Quantitative Pilot Study [50]

Mouiche et al. evaluated the antimicrobial usage in modern layer poultry farms in the West Region of Cameroon. 70 layer poultry farms and 4 veterinary pharmacies were surveyed. The most sold antimicrobials by veterinary pharmacies were tetracyclines, sulfonamides, quinolones, and nitrofurans. In addition, four-fifths of sales corresponded to the WHO categorization of antimicrobials according to their importance in human medicine. Frequent practice of metaphylactic and prophylactic treatments was observed with underdosing or overdosing of some critically important antimicrobials for human medicine. The poultry herd size, the density of hens per square meter, and the livestock ratio per employee were observed as risk factors for antimicrobial dosage in poultry farms in the study area. Multivariable logistic regression showed that poultry herd sizes of fewer than 5,000 hens were associated with underdosage of antimicrobials, while poultry herd sizes between 5,000 and 10,000 hens were observed to be significantly associated with overdosage of antimicrobials. Densities $<7/m^2$ were significantly associated with overdosage of antimicrobials. A livestock ratio of fewer than 2,000 hens per employee was significantly associated with overdosage of antimicrobials.

Prevalence and risk factors for carriage of antimicrobial-resistant *Escherichia coli* on household and small-scale chicken farms in the Mekong Delta of Vietnam [51]

Nguyen et al. studied associations of farming practices and antimicrobial usage with resistant *E. coli* in household and small-scale chicken farms in Vietnam. They collected data and samples from 208 chicken farms. They found that quinolone and tetracycline

usage, small farm size, usage of commercial feed, the non-practising of AIAO (all-in-all-out) system and the practice of changing shoots/boots were associated with resistance to ciprofloxacin, however, these associations were not independent. In all-in-all-out systems, animals move through the stages of production as a group, often the same age, and new animals are not added to the group. The room is completely emptied, cleaned and disinfected before the introduction of the next batch. Significant interactions were also observed between small farm size and the practice of changing shoes/boots, and between the usage of commercial feed and the use of the AIAO system. As regards gentamicin resistance, lincosamide and tetracycline usage, the practice of changing shoes/boots, the purchase of day-old chickens from non-industrial sources (local hatcheries, markets, neighbours, etc.), and raising chickens for other purposes than egg-laying were all factors associated with isolation of resistant *E. coli*. Increasing chicken density (chickens per m2) by one unit increased the odds of isolating gentamicin or MDR resistant *E. coli* by 32 and 28%, respectively. The use of commercial feed was also associated, while the farmer's years of experience in chicken production was inversely associated (4% decrease for a one unit increase) with MDR *E. coli*.

Resistance to third generation cephalosporins was associated with the presence of fish pond(s) on the farm and the usage of any antimicrobial. The presence of fish pond(s) was also associated with ESBL-producing *E. coli* on the farm, together with the purchase of day-old chicks from other than industrial companies and having a change shoes/boots practice on the farm. According to the authors, the fact that the use of commercial feed was associated with the risk of resistance to different antimicrobials reflects the fact that commercial poultry feed is commonly medicated with antimicrobials in Vietnam. The intensification of chicken production had mixed effect on AMR. On one hand, isolates from household farms had lower levels of ciprofloxacin resistance than those of small farms. On the other hand, an increase in chicken density was associated with gentamicin- and multidrug resistance. AIAO systems, being more characteristic of larger farms, however, were inversely associated with ciprofloxacin resistance. Purchasing day-old chicks from industrial companies was associated with lower levels of gentamicin resistance.

Genomic epidemiological analysis of mcr-1-harboring *Escherichia coli* collected from livestock settings in Vietnam [52]

Nguyen et al, using whole genome sequencing, analysed the molecular epidemiological characteristics, history and relatedness of 50 *E. coli* isolates obtained from different farm and market reservoirs in Vietnam. Their data suggested that the epidemiology of the mcr-1 gene was mostly determined by plasmid spreading instead of clonal dissemination. Furthermore, they identified several sequence types in flies with genetic similarity to STs isolated from other reservoirs (ST206, ST2705, ST155, ST10, and ST48) suggesting that flies contributed to the transmission of AMR bacteria,

though, they concluded the evidence was yet insufficient to make the assertion about the role of flies in disseminating AMR genes in livestock environments.

High levels of antimicrobial resistance among Escherichia Coli isolates from livestock farms and synanthropic rats and shrews in the mekong delta of Vietnam [53]

Nhung et al. analysed *E. coli* isolates from 90 pig, chicken and duck farms, and compared the results to *E. coli* isolates from 66 small wild mammals (rats and shrews) trapped on farms and in forests and rice fields. Data were also collected regarding farm management practices, experience of disease in the flocks/herds, and other factors such as the usage of antimicrobial formulations over the previous 90 days. Potential factors associated with multidrug resistance (MDR) such as main farmed species, farm size, farmer demographics, recent use of antimicrobials (90 days), and type of water and feed were investigated by hierarchical logistical regression. The age of farmer was independently associated with MDR (per 10-year period). They observed lower levels of AMR on farms run by older farmers, which was probably a reflection of more experience in farming, resulting in better disease control with fewer antimicrobials. They found, after adjusting for host species, that the prevalence of multidrug resistance was 8 times greater for wild animals trapped on farms than those trapped in forests/rice fields. The results strongly suggest that AMR on farms is a key driver of environmental AMR in the Mekong Delta.

Horizontal gene transfer is the main driver of antimicrobial resistance in broiler chicks infected with *Salmonella enterica* serovar Heidelberg [54]

Oladeinde et al. challenged neonatal Cobb 500 broiler chicks with an antibiotic-susceptible *Salmonella* Heidelberg strain via various routes of inoculation, and found that the chicks grown without antibiotics harbored an antimicrobial resistant S. Heidelberg population 14 days after the challenge. They also observed that chicks challenged orally carried a higher percentage (46%) of AR S. Heidelberg isolates than cloacally inoculated (24%) or seeder (8%) chicks. It was found that infection by S. Heidelberg perturbed the microbiota of the chicks and a commensal *E. coli* population was confirmed to be the main reservoir of an IncI1 plasmid which was acquired by S. Heidelberg. These results suggest the role of horizontal gene transfer by plasmids in carrying resistance, suggesting that the reduction of antibiotic use alone is not sufficient to limit the transfer of antibiotic resistance.

They also found differences in the rate of AR acquisition between two trials they conducted. They hypothesized that high house temperature, litter moisture, and litter pH selected for an S. Heidelberg population that had no plasmid-borne AR.

Antimicrobial Drug Administration and Antimicrobial Resistance of Salmonella Isolates Originating from the Broiler Production Value Chain in Nigeria [55]

This study explored the dynamics and patterns of antimicrobial drug administration in the Nigeria broiler production value chain (NBPVC) and evaluated the antimicrobial resistance of *Salmonella* isolates originating therefrom. Based on the antimicrobial drugs sampled, the most abused drugs in the Nigeria broiler production value chain (NBPVC) using the FARAD specification were enrofloxacin and tetracycline. Twelve out of the 21 drugs sampled had high rates of indiscriminate administration without laboratory test or veterinary prescription; these include amoxicillin-clavulanic, ampicillin, ciprofloxacin, colistin, doxycycline, erythromycin, gentamycin, neomycin, penicillin, cotrimoxazole (trimethoprim-sulphamethoxazole), tylosine, and furaltadone. Antimicrobial drugs that are not commonly used and not arbitrarily administered in the NBPVC are few and include ceftriaxone, florfenicol, flumequine, and tiamulin. The highest resistance was observed against flumequine (100%), penicillin (95.4%), perfloxacin (89.6%), ampicillin (88.5%), and enrofloxacin (81%). AMR patterns for the 18 tested antibiotics indicated that 11/18 showed very high levels of resistance for the *Salmonella* isolates tested, including amoxicillin-clavulanic, ampicillin, ciprofloxacin, enrofloxacin, erythromycin, flumequine, neomycin, penicillin, perfloxacin, sulfonamides, and tetracycline. Three of the antibiotics (colistin sulphate, doxycycline, and norfloxacin) revealed high resistance patterns. However, ceftriaxone and florfenicol displayed low resistance patterns, while gentamicin and streptomycin revealed very low resistance patterns. These latter antibiotics appear to be the most active against the *Salmonella* isolates in this work. This pattern was reflected in all classes of antibiotics studied, suggesting a link between antimicrobial usage patterns and AMR development.

Knowledge, Attitudes, and Risk Perception of Broiler Grow-Out Farmers on Antimicrobial Use and Resistance in Oyo State, Nigeria [56]

Oloso et al. assessed the knowledge, attitudes and risk perception of Nigerian broiler grow-out farmers. According to their results, marital status, farm category, educational specialization, sales target, growth duration/cycle, broiler stocking batch, and feed source were factors significantly associated with knowledge levels of farmers.

Industrial broiler producers were more likely to have adeqate knowledge than their commercial counterparts. In addition, those with tertiary/post-secondary education and those with agriculture/veterinary-oriented post-secondary educational specialization were more likely to have satisfactory knowledge. In regards to broiler stocking/batch, those reporting higher broiler stocking had more satisfactory knowledge than those with less than 5000 broiler stock. On the other hand, those reporting 40-56 days and >56 days growth duration/cycles were significantly less likely to have satisfactory knowledge compared to those of <40 days growth duration/cycle.

The factors mentioned above were also significantly associated with attitudes of respondents, with the addition of age category and experience as broiler farmer. Farmers in the higher age categories and those with 25-35 years of experience as broiler farmers were less likely to have satisfactory attitudes compared to those with 1-10 years of experience.

In regards to feed source, self-compounding milled at a feed mill and usage of finished commercial feeds were found to be associated more to unsatisfactory knowledge levels of AMU and AMR than self-compounding and milling.

The authors found that farmers were more concerned about economic survival, with little consideration for the future, leading to an unwillingness to improve regarding AMR issues.

Antimicrobial Usage Factors and Resistance Profiles of Shiga Toxin-Producing *Escherichia coli* in Backyard Production Systems From Central Chile [57]

The aim of the study of Pavez-Muñoz et al. was to characterize phenotypic and genotypic antimicrobial resistance (AMR) and to study the epidemiology of Shiga toxin-producing *Escherichia coli* (STEC) isolated in backyard production systems (BPS) from Metropolitana region, Chile. A total of 85 BPS were sampled. Seven hundred and twelve (712) samples were collected from animals raised in BPS (63). Of these, 531 (74.6%) corresponds to hens samples, followed by 55 (7.7%) duck samples, 25 (3.5%) swine samples, 20 (2.8%) goose samples, and 81 (11.4%) samples belonging to small ruminants, horses, and other domestic animals that represent <2% of the total samples each one. A total of 20 samples (2.81%) belonging to 10 BPS (11.76%) were detected positive to STEC by PCR. Positivity to STEC was detected in 9 sheep (45%), 3 dairy cattle (15%), 3 ducks (15%), 2 goats (10%), 2 hens (10%), and 1 swine (5%). No environmental samples were detected as STEC positive. All strains were cephalexin-resistant (100%, n = 10), and five strains were resistant to chloramphenicol (50%). Principal component analysis showed that BPS size, number of cattle, pet and horse, and elevation act as driver of antimicrobial usage. Smaller BPS and the ones with lower number of cattle tend to have more chances of using AM. BPS located closer to 0 meters above sea level have more chances of using AM. The presence of pets decrease the probability of AM usage and lower number of horses increase the risk of AM usage. The recognition of diseases in animals, keeping poultry and/or swine in neighboring BPS, the visit of Veterinary Officials and the close contact between animal species in the BPS all increase the risk of antimicrobial use.

Knowledge, attitudes and practices of livestock and aquaculture producers regarding antimicrobial use and resistance in Vietnam [58]

Pham-Duc et al. surveyed the KAP of small- and medium-scale producers specialized in pig, poultry and aquaculture production in Vietnam. The results showed that those with higher levels of formal education had better knowledge and attitude than those with lower education, though the reported practices of both groups were similar.

Factors positively associated with more favorable attitudes were higher levels of education, smaller scale farming, keeping livestock penned, keeping records of antibiotic use, and sufficient levels of knowledge.

The primary reason for antibiotic use reported by producers was the treatment of infections (69%), but prophylactic use was also evident, for instance, 55%, 25% and 27% indicated situations as "animals display abnormal symptoms or behaviour", "weather is about to change" and the "animals on neighboring farms fall ill" as motivations for antibiotic use.

Furthermore, at the first indication of disease farmers preferred to use antibiotics at first place (17%), compared to hygienic (10%) or quarantine (5%) measures. Antibiotics were also given by producers to guarantee the health of the animals to ensure their profit.

Different kettles of fish: Varying patterns of antibiotic use on pig, chicken and fish farms in Lao PDR and implications for antimicrobial resistance strategies [59]

This study aimed to explore the views and practices of commercial pig, poultry and fish farmers regarding antibiotics in Lao People's Democratic Republic. A total of 364 farmers, corresponding to 454 farm units, were surveyed using a questionnaire and farm visits. Results showed that antibiotics were found less frequently in fish farm units compared to pig and poultry farm units, and more frequently in specialized farms (one species only) than in livestock-fish farms. Multiple factor analysis and hierarchical cluster analysis revealed three profiles of farmers, each with distinct patterns on knowledge, attitudes and practices regarding ABU (antibacterial use) and AMR. Cluster 1 held a positive attitude regarding preventive measures and information about antibiotics. In cluster 2, there was a view that antibiotics should be used for prophylactic treatment such as disease prevention. Cluster 3 was characterized by farmers with weak knowledge who were unfamiliar with antibiotics and uncertain about details concerning antibiotic use. This cluster was associated with a significantly lower use of antibiotics than the two other clusters in the regression model. None of the farmers' specific knowledge was under or overrepresented in clusters 1 and 2, meaning that farmers from these two clusters had a heterogeneous level of knowledge. In cluster 1, farmers show a better attitude than farmers in cluster 2 in terms of preventive measures (such as application of hygienic and biosecurity measures), but this is not reflected in their ABU, as the logistic regression model does not show a difference of ABU between clusters 1 and 2. ABU was less frequently observed in units where farmers were more than 51-year old in comparison with less

than 20-year old, and in farms where farmers did not know if any disease occurred in the past 12 months.

Residual concentrations of antimicrobial growth promoters in poultry litter favour plasmid conjugation among *Escherichia coli* [60]

Saraiva et al. studied the effects of low concentrations of antimicrobial growth promoters (AGPs) in poultry litter on the frequencies of IncFII-FIB plasmid conjugation among *E. coli*. The *in vitro* trial was conducted on two types of poultry litter materials (sugarcane bagasse and wood shavings) and with five treatments of litter, including the herbal alkaloid sanguinarine, and the antimicrobial growth promoters monensin, lincomycin and virginiamycin beside the control. The authors found that the frequency of plasmid conjugation among *E. coli* was increased by the presence of monensin, lincomycin and virginiamycin in both litter types. A significant interaction was observed between the type of litter and the antimicrobial residues on plasmid conjugation. Higher conjugation frequencies were found in wood shavings compared to sugarcane bagasse in the presence of AGPs (regardless of the type of antimicrobial), which was not observed with sanguinarine and the control. Sanguinarine, however, significantly reduced plasmid conjugation in sugarcane bagasse litter, compared to the AGPs.

Considering that an increased plasmid conjugation was observed with subinhibitory antimicrobial concentrations, the findings raised questions whether a similar phenomenon could be observed with drugs for therapeutic use in poultry production.

Knowledge, Attitudes, and Practices Related to Antibiotic Use and Antibiotic Resistance among Poultry Farmers in Urban and Peri-Urban Areas of Ouagadougou, Burkina Faso [61]

Sawadogo et al. conducted a cross-sectional survey with poultry farmers regarding their knowledge, attitudes and practices on the use of antibiotics in Burkina Faso. Half of the respondents reported using antibiotics for prevention, and in case of disease outbreaks, 32% of farmers used veterinary drugs without prescription.

Multivariate analysis demonstrated that a good attitude adopted during the last disease on the farm significantly and positively influenced the degree of knowledge. Treatment success, however, was negatively associated with the degree of knowledge.

The size of the farm positively influenced certain practices. Those with larger farms more likely consulted a qualified veterinarian in case of outbreaks, and were more likely to pay for veterinary drugs in formal drug stores.

Education level was a factor influencing different aspects. Those with higher education were more likely to use a qualified veterinarian. On the other hand, they were more

likely to use antibiotics for prevention or growth promotion, even in case they contacted the right health professional. This was explained by the fact that higher education is associated with more means farmers have to buy antibiotics.

Notably, 79.19% of farmers used eggs and chicken meat from birds under teatment or right after treatment with an antibiotic.

Distribution and dissemination of antimicrobial-resistant *Salmonella* in broiler farms with or without enrofloxacin use [62]

The present study aimed to investigate the characteristics and dissemination of antimicrobial-resistant *Salmonella* within and between broiler farms that used enrofloxacin and those that did not. 660 cloacal and 924 environmental (396 litter, 264 feed, and 264 water) samples from two selected flocks in each of 12 farms owned by the same company were collected in South Korea. The rate of *Salmonella* isolation was 7.8% (123/1584). Azithromycin resistant (17.9%) and colistin-resistant (3.3%) isolates were detected, and multidrug-resistant isolates (43.1%) were also observed. No isolate was resistant to enrofloxacin or ciprofloxacin; however, intermediate resistance to enrofloxacin was significantly higher in farms that used enrofloxacin than in those that did not. The rate of multi-drug resistance among litter isolates (25/44, 56.8%) was significantly higher than that among cloacal swab (24/67, 35.8%) and feed (4/12, 33.3%) isolates. The study highlights the occurrence of horizontal transmission and cyclic contamination with antimicrobial-resistant *Salmonella* in broiler farms owned by the same company. Litter may be a good indicator of indoor environmental contamination with antimicrobial-resistant *Salmonella* on farms. Additionally, enrofloxacin use may be one of the factors promoting resistance towards it in *Salmonella*.

A metagenomic glimpse into the gut of wild and domestic animals: Quantification of antimicrobial resistance and more [63]

In their study Skarżyńska et al. tested faecal samples of intensively produced chickens, turkeys, and pigs, and also of wild animals such as wild boars, red foxes, and rodents in Poland. They applied shotgun metagenomics of total DNA to explore the abundance of different resistance genes, and to examine bacterial and plasmid composition. The study revealed higher AMR levels as well as higher resistome diversity and richness in domestic species, pointing to antimicrobial usage in the animal production sector as the main AMR driver. The results also indicate that wildlife constitutes a reservoir of AMR determinants including those encoding resistance to antimicrobials highly important in human medicine. The potential of wildlife as AMR transmission vectors has been proven by plasmid profiles revealed in wild boars and red foxes. The study also demonstrated that discrepancies between

AMR found in the intestinal microbiomes of various animals probably resulted from different antimicrobial exposure, habitat, and diet.

Determinants associated with veterinary antimicrobial prescribing in farm animals in the Netherlands: A qualitative study [64]

Speksnijder et al. conducted semi-structured interviews with eleven farm animal veterinarians in the Netherlands. Important issues raised by veterinarians concerning antimicrobial prescribing were perceptions of professional responsibilities, risk avoidance, financial dependency on clients and client pressure, farmers' management practices and compliance to veterinary advices, economic aspects hindering disease preventive actions, advisory competencies of veterinarians and personal beliefs on antimicrobial resistance. This study indicates that antimicrobial prescribing by veterinarians is influenced by a very complex set of internal attitudes and beliefs and external, often conflicting, interests. To reduce the overall use and misuse of antimicrobials in farm animals, three different challenges can be distinguished:

(i) the successful and consistent implementation of preventive measures at farm level, (ii) the reduction in thresholds for the use of diagnostics and (iii) the prudent and accurate administration of antimicrobial treatments.

In vitro transduction of antimicrobial resistance genes into *Escherichia coli* isolates from backyard poultry in mexico [65]

Talavera-González et al. collected *E. coli* isolates from chicken, turkeys and ducks in backyard production units and from migratory wild birds in Mexico, and obtained bacteriophages from the samples with the intention to demonstrate transduction of antimicrobial resistance genes. Four bacteriophages were isolated from MDR bacterial isolates collected from migratory birds, and these were able to infect a total of 8/13 (61.5%) nonresistant *E. coli* isolates from backyard poultry (4 from ducks and 4 from chicken), and a total of 11/14 (78.5%) non-resistant *E. coli* isolates from 6/9 (66.6%) wild birds' species. According to the authors, this was the first report that described the transduction of antimicrobial resistance genes (qnrA, tetB, blaTEM and sulII genes) from phages of migratory wild birds to poultry which suggested the possible transmission in backyard production units.

Epidemiology and antimicrobial resistance profiles of *Salmonella* in chickens, sewage, and workers of broiler farms in selected areas of Bangladesh [66]

A cross-sectional study was conducted to determine the prevalence and AMR pattern of *Salmonella* isolated from broiler chickens, farm sewage, and farm workers in Bangladesh. This study also aimed at identifying the risk factors for *Salmonella*

infection in chickens. Cloacal swabs (n = 50) from broiler chickens, farm sewage (n = 50), and hand washed water of farm workers (n = 50) were collected along with data on farm management and antimicrobial usage from 50 broiler farms. All samples were analysed for the presence of *Salmonella*. Antimicrobial susceptibility test was done for ten antimicrobials. The overall *Salmonella* prevalence was 66% (99/150). *Salmonella* prevalence were 82% and 72% in cloacal swabs and farm sewage samples, respectively. From hand washed water, 44% of the samples were positive for *Salmonella*. *Salmonella* infection in broiler chickens was significantly associated with farming experience (≤ 5 years) and age of birds (≥ 11 days). The highest resistance was observed against colistin (88.9%) and doxycycline (84.8%) followed by ciprofloxacin (78.8%) and ceftazidime (64.6%), while the lowest resistance was observed against levofloxacin (15.2%). Resistance to carbapenems namely imipenem and meropenem were 36.4% and 18.2% respectively, though they are not used in poultry practices in Bangladesh. About 90% of *Salmonella* isolates exhibited MDR.

Farm level risk factors for fluoroquinolone resistance in *E. coli* and thermophilic *Campylobacter spp*. on poultry farms [67]

Data on husbandry practices, performance, disease and drug use were collected during a cross-sectional survey of 89 poultry meat farms in England and Wales to provide information on possible risk factors for the occurrence of fluoroquinolone (FQ)-resistant bacteria. Faeces samples were used to classify farms as “affected” or “not affected” by FQ-resistant (FQr) *Escherichia coli* or *Campylobacter* spp. Risk factor analysis identified the use of FQ on the farms as having by far the strongest association, among the factors considered, with the occurrence of FQr bacteria. Resistant *E. coli* and/or *Campylobacter* spp. were found on 86% of the farms with a history of FQ use. However, a substantial proportion of farms with no history of FQ use also yielded FQr organisms, suggesting that resistant bacteria may transfer between farms. Significant factors increasing the risk of occurrence of FQr *E. coli* are the use of FQ in past, single-handed operation of the site, and the existence of a public footpath on the periphery of the site. The sole significant factor decreasing risk is enclosure of the site by a perimeter fence. Significant factors increasing the risk of occurrence of FQr *Campylobacter* spp. are also the use of FQ in the past and wild birds having access to poultry houses. Significant factors decreasing the risk are more than the median (for all broiler or turkey farms in the sample, as appropriate) number of birds on site, the site operated by an independent grower, masks provided for staff, detailed areas dusted before wet cleaning, and feed hoppers cleaned and disinfected.

Prevalence of antimicrobial resistance and potential pathogenicity, and possible spread of third generation cephalosporin resistance, in *Escherichia coli* isolated from healthy chicken farms in the region of Dakar, Senegal [68]

Vounba et al. isolated *E. coli* from healthy chicken in Senegal, to determine their antimicrobial profiles and virulence mechanisms, investigate the spread of third-generation cephalosporins (3GC) resistance in *E. coli* and determine whether resistance to 3GC could be linked to risk factors.

They found a discrepancy between the high AMR prevalence observed and the low reported use of antimicrobials. They mentioned several potential reasons, eg. breeders not reporting antimicrobial use, use in previous flocks, potential use in hatching eggs imported from other continents, usage of Virkon, a disinfectant in chicken farms leading to resistance without quinolone use, but they were not confirmed. A multivariate logistic regression included trained staff, farms with at least two buildings housing chickens, lack of observance of an empty period, allowing visitors on the farm, and disposing of dead chickens into the environment as potential risk factors for 3GC resistant *E. coli* on farms, but none was found to be significantly associated.

The authors mentioned the low statistical power related to the small sample size (32 farms) as the potential reason for the lack of significance. They further noted that some breeders produce irregularly and only in times of high demand, and the study was conducted in a period when poultry farms were generally rare.

3GC resistant isolates had a greater tendency of occurrence on farms with reported veterinary skills, which suggested the breeders with veterinary knowledge were using more antimicrobial agents. The results suggested both clonal spreading and horizontal gene transfer to play a role in the spread of 3GC resistance.

<u>Risk factors for the abundance of antimicrobial resistance genes aph(3′)-III, erm(B), sul2 and tet(W) in pig and broiler faeces in nine European countries</u> [69]

Yang et al. investigated AMR levels of 179 pig farms and 180 broiler farms in nine European countries, and aimed to determine potential risk factors for antimicrobial resistance, using a questionnaire collecting information on antimicrobial use and other farm characteristics. Faeces of animals were analysed by real-time quantitative PCR for the relative abundance of four antimicrobial resistance genes (ARGs). A mixed model using country and farm as random effects was used to evaluate the relationship between AMR and AMU and other farm characteristics. In both univariable analysis and the multivariable model without AMU of broilers, significant associations were found between relative ARG abundances and the number of farmworkers (erm(B) and tet(W)), weight of broilers at set-up (erm(B)), average number of rounds per year (sul2), disease management (tet(W)) and removal of manure and carcasses (aph(3′)-III, erm(B), tet(W)). Compared to the univariable model for broilers, fewer variables were left in the multivariable model without AMU („cleaning and disinfection" and „visitors and farmworkers" for aph(3′)-III). Furthermore, a significant negative association was found between tet(W) abundance and „cleaning and disinfection" in

the multivariable model without AMU. Considering antimicrobial use, a significant positive association was found between corresponding ARG and AMU levels. Adjustment for AMU led to only minor changes in the outcome of the multivariable model for broilers. The authors found a positive association between the biosecurity scores of 'transfer of faeces and carcasses' including 'removal of farm manure'. As previous studies had reported manure storage on farm to be negatively associated with resistance, the authors noted that the relationship between on-farm biosecurity measures and AMR levels in animals is complex and more analyses would be necessary to understand the impact of possible interventions. The authors also found that the studied farm characteristics only explained a limited part of the observed total AMR variation, indicating that there were likely unidentified determinants of AMR which still need to be evaluated in future works.

References

1. Abraham, S.; Sahibzada, S.; Hewson, K.; Laird, T.; Abraham, R.; Pavic, A.; Truswell, A.; Lee, T.; O'Dea, M.; Jordan, D. Emergence of Fluoroquinolone-Resistant Campylobacter Jejuni and Campylobacter Coli among Australian Chickens in the Absence of Fluoroquinolone Use. *Appl. Environ. Microbiol.* **2020**, *86*, doi:10.1128/AEM.02765-19.
2. Alhaji, N.B.; Haruna, A.E.; Muhammad, B.; Lawan, M.K.; Isola, T.O. Antimicrobials Usage Assessments in Commercial Poultry and Local Birds in North-Central Nigeria: Associated Pathways and Factors for Resistance Emergence and Spread. *Prev. Vet. Med.* **2018**, *154*, 139–147, doi:10.1016/j.prevetmed.2018.04.001.
3. Aliyu, A.B.; Saleha, A.A.; Jalila, A.; Zunita, Z. Risk Factors and Spatial Distribution of Extended Spectrum β-Lactamase-Producing- Escherichia Coli at Retail Poultry Meat Markets in Malaysia: A Cross-Sectional Study. *BMC Public Health* **2016**, *16*, doi:10.1186/s12889-016-3377-2.
4. Ashwini, A.; Jamwal, P.; Vanak, A.T. Environmental Surveillance of Antimicrobial Resistance in a Rapidly Developing Catchment. *Environ. Monit. Assess.* **2023**, *195*, doi:10.1007/s10661-022-10630-7.
5. Aworh, M.K.; Kwaga, J.; Okolocha, E.; Mba, N.; Thakur, S. Prevalence and Risk Factors for Multi-Drug Resistant Escherichia Coli among Poultry Workers in the Federal Capital Territory, Abuja, Nigeria. *PLoS ONE* **2019**, *14*, doi:10.1371/journal.pone.0225379.
6. Ayoola, M.B.; Pillai, N.; Nanduri, B.; Rothrock, M.J.; Ramkumar, M. Preharvest Environmental and Management Drivers of Multidrug Resistance in Major Bacterial Zoonotic Pathogens in Pastured Poultry Flocks. *Microorganisms* **2022**, *10*, doi:10.3390/microorganisms10091703.
7. Bastard, J.; Nhung, N.T.; Hien, V.B.; Kiet, B.T.; Temime, L.; Opatowski, L.; Carrique-Mas, J.; Choisy, M. Modelling the Impact of Antimicrobial Use and External Introductions on Commensal E. Coli Colistin Resistance in Small-Scale Chicken Farms of the Mekong Delta of Vietnam. *Transbound. Emerg. Dis.* **2022**, *69*, e2185–e2194, doi:10.1111/tbed.14558.
8. Bâtie, C.; Ha, L.T.T.; Loire, E.; Truong, D.B.; Tuan, H.M.; Cuc, N.T.K.; Paul, M.; Goutard, F. Characterisation of Chicken Farms in Vietnam: A Typology of Antimicrobial Use

among Different Production Systems. *Prev. Vet. Med.* **2022**, *208*, doi:10.1016/j.prevetmed.2022.105731.
9. Benavides, J.A.; Streicker, D.G.; Gonzales, M.S.; Rojas-Paniagua, E.; Shiva, C. Knowledge and Use of Antibiotics among Low-Income Small-Scale Farmers of Peru. *Prev. Vet. Med.* **2021**, *189*, doi:10.1016/j.prevetmed.2021.105287.
10. Bhargavi, D.; Sahu, R.; Nishanth, M.A.D.; Doijad, S.P.; Niveditha, P.; Kumar, O.R.V.; Sunanda, C.; Girish, P.S.; Naveena, B.M.; Vergis, J.; et al. Genetic Diversity and Risk Factor Analysis of Drug-Resistant Escherichia Coli Recovered from Broiler Chicken Farms. *Comp. Immunol. Microbiol. Infect. Dis.* **2023**, *93*, doi:10.1016/j.cimid.2022.101929.
11. Boulianne, M.; Arsenault, J.; Daignault, D.; Archambault, M.; Letellier, A.; Dutil, L. Drug Use and Antimicrobial Resistance among Escherichia Coli and Enterococcus Spp. Isolates from Chicken and Turkey Flocks Slaughtered in Quebec, Canada. *Can. J. Vet. Res.* **2016**, *80*, 49–59.
12. Buberg, M.L.; Witsø, I.L.; L'Abée-Lund, T.M.; Wasteson, Y. Zinc and Copper Reduce Conjugative Transfer of Resistance Plasmids from Extended-Spectrum Beta-Lactamase-Producing Escherichia Coli. *Microb. Drug Resist.* **2020**, *26*, 842–849, doi:10.1089/mdr.2019.0388.
13. Bythwood, T.N.; Soni, V.; Lyons, K.; Hurley-Bacon, A.; Lee, M.D.; Hofacre, C.; Sanchez, S.; Maurer, J.J. Antimicrobial Resistant Salmonella Enterica Typhimurium Colonizing Chickens: The Impact of Plasmids, Genotype, Bacterial Communities, and Antibiotic Administration on Resistance. *Front. Sustain. Food Syst.* **2019**, *3*, doi:10.3389/fsufs.2019.00020.
14. Caffrey, N.; Agunos, A.; Gow, S.; Liljebjelke, K.; Waldner, C.L.; Mainali, C.; Checkley, S.L. A Cross-Sectional Study of the Prevalence Factors Associated with Fluoroquinolone Resistant Campylobacter Jejuni in Broiler Flocks in Canada. *Prev. Vet. Med.* **2021**, *186*, doi:10.1016/j.prevetmed.2020.105164.
15. Caffrey, N.; Nekouei, O.; Gow, S.; Agunos, A.; Checkley, S. Risk Factors Associated with the A2C Resistance Pattern among E. Coli Isolates from Broiler Flocks in Canada. *Prev. Vet. Med.* **2017**, *148*, 115–120, doi:10.1016/j.prevetmed.2017.11.001.
16. Carrique-Mas, J.J.; Trung, N.V.; Hoa, N.T.; Mai, H.H.; Thanh, T.H.; Campbell, J.I.; Wagenaar, J.A.; Hardon, A.; Hieu, T.Q.; Schultsz, C. Antimicrobial Usage in Chicken Production in the Mekong Delta of Vietnam. *Zoonoses Public Health* **2015**, *62*, 70–78, doi:10.1111/zph.12165.
17. Caucci, C.; Di Martino, G.; Dalla Costa, A.; Santagiuliana, M.; Lorenzetto, M.; Capello, K.; Mughini-Gras, L.; Gavazzi, L.; Bonfanti, L. Trends and Correlates of Antimicrobial Use in Broiler and Turkey Farms: A Poultry Company Registry-Based Study in Italy. *J. Antimicrob. Chemother.* **2019**, *74*, 2784–2787, doi:10.1093/jac/dkz212.
18. Caudell, M.A.; Dorado-Garcia, A.; Eckford, S.; Creese, C.; Byarugaba, D.K.; Afakye, K.; Chansa-Kabali, T.; Fasina, F.O.; Kabali, E.; Kiambi, S.; et al. Towards a Bottom-up Understanding of Antimicrobial Use and Resistance on the Farm: A Knowledge, Attitudes, and Practices Survey across Livestock Systems in Five African Countries. *PLoS ONE* **2020**, *15*, doi:10.1371/journal.pone.0220274.
19. Chowdhury, S.; Fournié, G.; Blake, D.; Henning, J.; Conway, P.; Hoque, MdA.; Ghosh, S.; Parveen, S.; Biswas, P.K.; Akhtar, Z.; et al. Antibiotic Usage Practices and Its Drivers in Commercial Chicken Production in Bangladesh. *PLoS ONE* **2022**, *17*, doi:10.1371/journal.pone.0276158.

20. Coyne, L.; Patrick, I.; Arief, R.; Benigno, C.; Kalpravidh, W.; McGrane, J.; Schoonman, L.; Sukarno, A.H.; Rushton, J. The Costs, Benefits and Human Behaviours for Antimicrobial Use in Small Commercial Broiler Chicken Systems in Indonesia. *Antibiotics* **2020**, *9*, doi:10.3390/antibiotics9040154.
21. Dione, M.M.; Amia, W.C.; Ejobi, F.; Ouma, E.A.; Wieland, B. Supply Chain and Delivery of Antimicrobial Drugs in Smallholder Livestock Production Systems in Uganda. *Front. Vet. Sci.* **2021**, *8*, doi:10.3389/fvets.2021.611076.
22. Elmi, S.A.; Simons, D.; Elton, L.; Haider, N.; Hamid, M.M.A.; Shuaib, Y.A.; Khan, M.A.; Othman, I.; Kock, R.; Osman, A.Y. Identification of Risk Factors Associated with Resistant Escherichia Coli Isolates from Poultry Farms in the East Coast of Peninsular Malaysia: A Cross Sectional Study. *Antibiotics* **2021**, *10*, 1–17, doi:10.3390/antibiotics10020117.
23. Eltholth, M.; Govindaraj, G.; Das, B.; Shanabhoga, M.B.; Swamy, H.M.; Thomas, A.; Cole, J.; Shome, B.R.; Holmes, M.A.; Moran, D. Factors Influencing Antibiotic Prescribing Behavior and Understanding of Antimicrobial Resistance Among Veterinarians in Assam, India. *Front. Vet. Sci.* **2022**, *9*, doi:10.3389/fvets.2022.864813.
24. Hassan, M.M.; Kalam, M.A.; Alim, M.A.; Shano, S.; Nayem, M.R.K.; Badsha, M.R.; Mamun, M.A.A.; Hoque, A.; Tanzin, A.Z.; Nath, C.; et al. Knowledge, Attitude, and Practices on Antimicrobial Use and Antimicrobial Resistance among Commercial Poultry Farmers in Bangladesh. *Antibiotics* **2021**, *10*, doi:10.3390/antibiotics10070784.
25. Huber, L.; Agunos, A.; Gow, S.P.; Carson, C.A.; Van Boeckel, T.P. Reduction in Antimicrobial Use and Resistance to Salmonella, Campylobacter, and Escherichia Coli in Broiler Chickens, Canada, 2013-2019. *Emerg. Infect. Dis.* **2021**, *27*, 2434–2444, doi:10.3201/eid2709.204395.
26. Ibrahim, R.A.; Cryer, T.L.; Lafi, S.Q.; Basha, E.-A.; Good, L.; Tarazi, Y.H. Identification of Escherichia Coli from Broiler Chickens in Jordan, Their Antimicrobial Resistance, Gene Characterization and the Associated Risk Factors. *BMC Vet. Res.* **2019**, *15*, doi:10.1186/s12917-019-1901-1.
27. Islam, Md.Z.; Islam, Md.S.; Kundu, L.R.; Ahmed, A.; Hsan, K.; Pardhan, S.; Driscoll, R.; Hossain, Md.S.; Hossain, Md.M. Knowledge, Attitudes and Practices Regarding Antimicrobial Usage, Spread and Resistance Emergence in Commercial Poultry Farms of Rajshahi District in Bangladesh. *PLoS ONE* **2022**, *17*, doi:10.1371/journal.pone.0275856.
28. Jiménez-Belenguer, A.; Doménech, E.; Villagrá, A.; Fenollar, A.; Ferrús, M.A. Antimicrobial Resistance of Escherichia Coli Isolated in Newly-Hatched Chickens and Effect of Amoxicillin Treatment during Their Growth. *Avian Pathol.* **2016**, *45*, 501–507, doi:10.1080/03079457.2016.1168515.
29. Kakooza, S.; Munyiirwa, D.; Ssajjakambwe, P.; Kayaga, E.; Tayebwa, D.S.; Ndoboli, D.; Basemera, L.; Nabatta, E.; Tumwebaze, M.A.; Kaneene, J.B. Epidemiological Dynamics of Extended-Spectrum β -Lactamase- or AmpC β -Lactamase-Producing Escherichia Coli Screened in Apparently Healthy Chickens in Uganda. *Scientifica* **2021**, *2021*, doi:10.1155/2021/3258059.
30. Kalam, Md.A.; Alim, Md.A.; Shano, S.; Nayem, Md.R.K.; Badsha, Md.R.; Mamun, Md.A.A.; Hoque, A.; Tanzin, A.Z.; Khan, S.A.; Islam, A.; et al. Knowledge, Attitude, and Practices on Antimicrobial Use and Antimicrobial Resistance among Poultry Drug and Feed Sellers in Bangladesh. *Vet. Sci.* **2021**, *8*, 111–111, doi:10.3390/vetsci8060111.

31. Kalam, M.A.; Rahman, M.S.; Alim, M.A.; Shano, S.; Afrose, S.; Jalal, F.A.; Akter, S.; Khan, S.A.; Islam, M.M.; Uddin, M.B.; et al. Knowledge, Attitudes, and Common Practices of Livestock and Poultry Veterinary Practitioners Regarding the AMU and AMR in Bangladesh. *Antibiotics* **2022**, *11*, doi:10.3390/antibiotics11010080.
32. Khan, X.; Lim, R.H.M.; Rymer, C.; Ray, P. Fijian Farmers' Attitude and Knowledge Towards Antimicrobial Use and Antimicrobial Resistance in Livestock Production Systems–A Qualitative Study. *Front. Vet. Sci.* **2022**, *9*, doi:10.3389/fvets.2022.838457.
33. Kimera, Z.I.; Frumence, G.; Mboera, L.E.G.; Rweyemamu, M.; Mshana, S.E.; Matee, M.I.N. Assessment of Drivers of Antimicrobial Use and Resistance in Poultry and Domestic Pig Farming in the Msimbazi River Basin in Tanzania. *Antibiotics* **2020**, *9*, 1–20, doi:10.3390/antibiotics9120838.
34. Koga, V.L.; Scandorieiro, S.; Vespero, E.C.; Oba, A.; De Brito, B.G.; De Brito, K.C.T.; Nakazato, G.; Kobayashi, R.K.T. Comparison of Antibiotic Resistance and Virulence Factors among Escherichia Coli Isolated from Conventional and Free-Range Poultry. *BioMed Res. Int.* **2015**, *2015*, doi:10.1155/2015/618752.
35. Koju, P.; Shrestha, R.; Shrestha, A.; Tamrakar, S.; Rai, A.; Shrestha, P.; Madhup, S.K.; Katuwal, N.; Shrestha, A.; Shrestha, A.; et al. Antimicrobial Resistance in E. Coli Isolated from Chicken Cecum Samples and Factors Contributing to Antimicrobial Resistance in Nepal. *Trop. Med. Infect. Dis.* **2022**, *7*, doi:10.3390/tropicalmed7090249.
36. Le Devendec, L.; Mourand, G.; Bougeard, S.; Léaustic, J.; Jouy, E.; Keita, A.; Couet, W.; Rousset, N.; Kempf, I. Impact of Colistin Sulfate Treatment of Broilers on the Presence of Resistant Bacteria and Resistance Genes in Stored or Composted Manure. *Vet. Microbiol.* **2016**, *194*, 98–106, doi:10.1016/j.vetmic.2015.11.012.
37. Lowenstein, C.; Waters, W.F.; Roess, A.; Leibler, J.H.; Graham, J.P. Animal Husbandry Practices and Perceptions of Zoonotic Infectious Disease Risks among Livestock Keepers in a Rural Parish of Quito, Ecuador. *Am. J. Trop. Med. Hyg.* **2016**, *95*, 1450–1458, doi:10.4269/ajtmh.16-0485.
38. Luiken, R.E.C.; Van Gompel, L.; Munk, P.; Sarrazin, S.; Joosten, P.; Dorado-García, A.; Borup Hansen, R.; Knudsen, B.E.; Bossers, A.; Wagenaar, J.A.; et al. Associations between Antimicrobial Use and the Faecal Resistome on Broiler Farms from Nine European Countries. *J. Antimicrob. Chemother.* **2019**, *74*, 2596–2604, doi:10.1093/jac/dkz235.
39. Luiken, R.E.; Heederik, D.J.; Scherpenisse, P.; Van Gompel, L.; van Heijnsbergen, E.; Greve, G.D.; Jongerius-Gortemaker, B.G.; Tersteeg-Zijderveld, M.H.; Fischer, J.; Juraschek, K.; et al. Determinants for Antimicrobial Resistance Genes in Farm Dust on 333 Poultry and Pig Farms in Nine European Countries. *Environ. Res.* **2022**, *208*, doi:10.1016/j.envres.2022.112715.
40. Luu, Q.H.; Nguyen, T.L.A.; Pham, T.N.; Vo, N.G.; Padungtod, P. Antimicrobial Use in Household, Semi-Industrialized, and Industrialized Pig and Poultry Farms in Viet Nam. *Prev. Vet. Med.* **2021**, *189*, doi:10.1016/j.prevetmed.2021.105292.
41. Makarov, D.A.; Ivanova, O.E.; Pomazkova, A.V.; Egoreva, M.A.; Prasolova, O.V.; Lenev, S.V.; Gergel, M.A.; Bukova, N.K.; Karabanov, S.Y. Antimicrobial Resistance of Commensal Enterococcus Faecalis and Enterococcus Faecium from Food-Producing Animals in Russia. *Vet. World* **2022**, *15*, 611–621, doi:10.14202/vetworld.2022.611-621.
42. Mandal, A.K.; Talukder, S.; Hasan, M.M.; Tasmim, S.T.; Parvin, M.S.; Ali, M.Y.; Islam, M.T. Epidemiology and Antimicrobial Resistance of Escherichia Coli in Broiler

Chickens, Farmworkers, and Farm Sewage in Bangladesh. *Vet. Med. Sci.* **2022**, *8*, 187–199, doi:10.1002/vms3.664.
43. Mankhomwa, J.; Tolhurst, R.; M'biya, E.; Chikowe, I.; Banda, P.; Mussa, J.; Mwasikakata, H.; Simpson, V.; Feasey, N.; MacPherson, E.E. A Qualitative Study of Antibiotic Use Practices in Intensive Small-Scale Farming in Urban and Peri-Urban Blantyre, Malawi: Implications for Antimicrobial Resistance. *Front. Vet. Sci.* **2022**, *9*, doi:10.3389/fvets.2022.876513.
44. Martínez, E.P.; Golding, S.E.; van Rosmalen, J.; Vinueza-Burgos, C.; Verbon, A.; van Schaik, G. Antibiotic Prescription Patterns and Non-Clinical Factors Influencing Antibiotic Use by Ecuadorian Veterinarians Working on Cattle and Poultry Farms: A Cross-Sectional Study. *Prev. Vet. Med.* **2023**, *213*, doi:10.1016/j.prevetmed.2023.105858.
45. Mezhoud, H.; Chantziaras, I.; Iguer-Ouada, M.; Moula, N.; Garmyn, A.; Martel, A.; Touati, A.; Smet, A.; Haesebrouck, F.; Boyen, F. Presence of Antimicrobial Resistance in Coliform Bacteria from Hatching Broiler Eggs with Emphasis on ESBL/AmpC-Producing Bacteria. *Avian Pathol.* **2016**, *45*, 493–500, doi:10.1080/03079457.2016.1167837.
46. Mo, S.S.; Kristoffersen, A.B.; Sunde, M.; Nødtvedt, A.; Norström, M. Risk Factors for Occurrence of Cephalosporin-Resistant Escherichia Coli in Norwegian Broiler Flocks. *Prev. Vet. Med.* **2016**, *130*, 112–118, doi:10.1016/j.prevetmed.2016.06.011.
47. Moffo, F.; Mouliom Mouiche, M.M.; Kochivi, F.L.; Dongmo, J.B.; Djomgang, H.K.; Tombe, P.; Mbah, C.K.; Mapiefou, N.P.; Mingoas, J.-P.K.; Awah-Ndukum, J. Knowledge, Attitudes, Practices and Risk Perception of Rural Poultry Farmers in Cameroon to Antimicrobial Use and Resistance. *Prev. Vet. Med.* **2020**, *182*, doi:10.1016/j.prevetmed.2020.105087.
48. Moffo, F.; Mouiche, M.M.M.; Djomgang, H.K.; Tombe, P.; Wade, A.; Kochivi, F.L.; Dongmo, J.B.; Mbah, C.K.; Mapiefou, N.P.; Ngogang, M.P.; et al. Poultry Litter Contamination by Escherichia Coli Resistant to Critically Important Antimicrobials for Human and Animal Use and Risk for Public Health in Cameroon. *Antibiotics* **2021**, *10*, doi:10.3390/antibiotics10040402.
49. Montoro-Dasi, L.; Villagra, A.; Sevilla-Navarro, S.; Pérez-Gracia, M.T.; Vega, S.; Marin, C. The Dynamic of Antibiotic Resistance in Commensal Escherichia Coli throughout the Growing Period in Broiler Chickens: Fast-Growing vs. Slow-Growing Breeds. *Poult. Sci.* **2020**, *99*, 1591–1597, doi:10.1016/j.psj.2019.10.080.
50. Mouiche, M.M.M.; Wouembe, F.D.K.; Mpouam, S.E.; Moffo, F.; Djuntu, M.; Toukam, C.M.W.; Kameni, J.M.F.; Okah-Nnane, N.H.; Awah-Ndukum, J. Cross-Sectional Survey of Prophylactic and Metaphylactic Antimicrobial Use in Layer Poultry Farming in Cameroon: A Quantitative Pilot Study. *Front. Vet. Sci.* **2022**, *9*, doi:10.3389/fvets.2022.646484.
51. Nguyen, V.T.; Carrique-Mas, J.J.; Ngo, T.H.; Ho, H.M.; Ha, T.T.; Campbell, J.I.; Nguyen, T.N.; Hoang, N.N.; Pham, V.M.; Wagenaar, J.A.; et al. Prevalence and Risk Factors for Carriage of Antimicrobial-Resistant Escherichia Coli on Household and Small-Scale Chicken Farms in the Mekong Delta of Vietnam. *J. Antimicrob. Chemother.* **2015**, *70*, 2144–2152, doi:10.1093/jac/dkv053.
52. Nguyen, P.T.L.; Ngo, T.H.H.; Tran, T.M.H.; Vu, T.N.B.; Le, V.T.; Tran, H.A.; Pham, D.T.; Nguyen, H.T.; Tran, D.L.; Nguyen, T.P.L.; et al. Genomic Epidemiological Analysis of Mcr-1-Harboring Escherichia Coli Collected from Livestock Settings in Vietnam. *Front. Vet. Sci.* **2022**, *9*, doi:10.3389/fvets.2022.1034610.

53. Nhung, N.T.; Cuong, N.V.; Campbell, J.; Hoa, N.T.; Bryant, J.E.; Truc, V.N.T.; Kiet, B.T.; Jombart, T.; Trung, N.V.; Hien, V.B.; et al. High Levels of Antimicrobial Resistance among Escherichia Coli Isolates from Livestock Farms and Synanthropic Rats and Shrews in the Mekong Delta of Vietnam. *Appl. Environ. Microbiol.* **2015**, *81*, 812–820, doi:10.1128/AEM.03366-14.
54. Oladeinde, A.; Abdo, Z.; Press, M.O.; Cook, K.; Cox, N.A.; Zwirzitz, B.; Woyda, R.; Lakin, S.M.; Thomas Iv, J.C.; Looft, T.; et al. Horizontal Gene Transfer Is the Main Driver of Antimicrobial Resistance in Broiler Chicks Infected with Salmonella Enterica Serovar Heidelberg. *mSystems* **2021**, *6*, doi:10.1128/mSystems.00729-21.
55. Oloso, N.O.; Adeyemo, I.A.; Heerden, H.V.; Fasanmi, O.G.; Fasina, F.O. Antimicrobial Drug Administration and Antimicrobial Resistance of Salmonella Isolates Originating from the Broiler Production Value Chain in Nigeria. *Antibiotics* **2019**, *8*, doi:10.3390/antibiotics8020075.
56. Oloso, N.O.; Odetokun, I.A.; Ghali-Mohammed, I.; Fasina, F.O.; Olatoye, I.O.; Adetunji, V.O. Knowledge, Attitudes, and Risk Perception of Broiler Grow-Out Farmers on Antimicrobial Use and Resistance in Oyo State, Nigeria. *Antibiotics* **2022**, *11*, doi:10.3390/antibiotics11050567.
57. Pavez-Muñoz, E.; González, C.; Fernández-Sanhueza, B.; Sánchez, F.; Escobar, B.; Ramos, R.; Fuenzalida, V.; Galarce, N.; Arriagada, G.; Neira, V.; et al. Antimicrobial Usage Factors and Resistance Profiles of Shiga Toxin-Producing Escherichia Coli in Backyard Production Systems From Central Chile. *Front. Vet. Sci.* **2021**, *7*, doi:10.3389/fvets.2020.595149.
58. Pham-Duc, P.; Cook, M.A.; Cong-Hong, H.; Nguyen-Thuy, H.; Padungtod, P.; Nguyen-Thi, H.; Dang-Xuan, S. Knowledge, Attitudes and Practices of Livestock and Aquaculture Producers Regarding Antimicrobial Use and Resistance in Vietnam. *PLoS ONE* **2019**, *14*, doi:10.1371/journal.pone.0223115.
59. Poupaud, M.; Goutard, F.L.; Phouthana, V.; Muñoz Viera, F.; Caro, D.; Patriarchi, A.; Paul, M.C. Different Kettles of Fish: Varying Patterns of Antibiotic Use on Pig, Chicken and Fish Farms in Lao PDR and Implications for Antimicrobial Resistance Strategies. *Transbound. Emerg. Dis.* **2022**, *69*, 3940–3951, doi:10.1111/tbed.14766.
60. Saraiva, M.M.S.; Silva, N.M.V.; Ferreira, V.A.; Moreira Filho, A.L.B.; Givisiez, P.E.N.; Freitas Neto, O.C.; Berchieri Júnior, A.; Gebreyes, W.A.; de Oliveira, C.J.B. Residual Concentrations of Antimicrobial Growth Promoters in Poultry Litter Favour Plasmid Conjugation among Escherichia Coli. *Lett. Appl. Microbiol.* **2022**, *74*, 831–838, doi:10.1111/lam.13671.
61. Sawadogo, A.; Kagambèga, A.; Moodley, A.; Ouedraogo, A.A.; Barro, N.; Dione, M. Knowledge, Attitudes, and Practices Related to Antibiotic Use and Antibiotic Resistance among Poultry Farmers in Urban and Peri-Urban Areas of Ouagadougou, Burkina Faso. *Antibiotics* **2023**, *12*, doi:10.3390/antibiotics12010133.
62. Shang, K.; Wei, B.; Kang, M. Distribution and Dissemination of Antimicrobial-Resistant Salmonella in Broiler Farms with or without Enrofloxacin Use. *BMC Vet. Res.* **2018**, *14*, doi:10.1186/s12917-018-1590-1.
63. Skarzynska, M.; Leekitcharoenphon, P.; Hendriksen, R.S.; Aarestrup, F.M.; Wasyl, D. A Metagenomic Glimpse into the Gut of Wild and Domestic Animals: Quantification of Antimicrobial Resistance and More. *PLoS ONE* **2020**, *15*, doi:10.1371/journal.pone.0242987.

64. Speksnijder, D.C.; Jaarsma, A.D.C.; van der Gugten, A.C.; Verheij, T.J.M.; Wagenaar, J.A. Determinants Associated with Veterinary Antimicrobial Prescribing in Farm Animals in the Netherlands: A Qualitative Study. *Zoonoses Public Health* **2015**, *62*, 39–51, doi:10.1111/zph.12168.
65. Talavera-González, J.M.; Talavera-Rojas, M.; Soriano-Vargas, E.; Vázquez-Navarrete, J.; Salgado-Miranda, C. In Vitro Transduction of Antimicrobial Resistance Genes into Escherichia Coli Isolates from Backyard Poultry in Mexico. *Can. J. Microbiol.* **2021**, *67*, 415–425, doi:10.1139/cjm-2020-0280.
66. Talukder, S.; Hasan, M.M.; Mandal, A.K.; Tasmim, S.T.; Parvin, M.S.; Ali, M.Y.; Nahar, A.; Islam, M.Z.; Islam, M.T. Epidemiology and Antimicrobial Resistance Profiles of Salmonella in Chickens, Sewage, and Workers of Broiler Farms in Selected Areas of Bangladesh. *J. Infect. Dev. Ctries.* **2021**, *15*, 1155–1166, doi:10.3855/jidc.14100.
67. Taylor, N.M.; Wales, A.D.; Ridley, A.M.; Davies, R.H. Farm Level Risk Factors for Fluoroquinolone Resistance in E. Coli and Thermophilic Campylobacter Spp. on Poultry Farms. *Avian Pathol.* **2016**, *45*, 559–568, doi:10.1080/03079457.2016.1185510.
68. Vounba, P.; Arsenault, J.; Bada-Alambédji, R.; Fairbrother, J.M. Prevalence of Antimicrobial Resistance and Potential Pathogenicity, and Possible Spread of Third Generation Cephalosporin Resistance, in Escherichia Coli Isolated from Healthy Chicken Farms in the Region of Dakar, Senegal. *PLoS ONE* **2019**, *14*, doi:10.1371/journal.pone.0214304.
69. Yang, D.; Heederik, D.J.J.; Mevius, D.J.; Scherpenisse, P.; Luiken, R.E.C.; Van Gompel, L.; Skarżyńska, M.; Wadepohl, K.; Chauvin, C.; Van Heijnsbergen, E.; et al. Risk Factors for the Abundance of Antimicrobial Resistance Genes Aph(3′)-III, Erm(B), Sul2 and Tet(W) in Pig and Broiler Faeces in Nine European Countries. *J. Antimicrob. Chemother.* **2022**, *77*, 969–978, doi:10.1093/jac/dkac002.
