# Supplementary material for: Scoping Review of Factors Affecting Antimicrobial Use and the Spread of Antimicrobial Resistance in the Poultry Production Chain
Source: Vet Sci. 2025 Sep 12;12(9):881. doi: 10.3390/vetsci12090881 (PMC12474243; doi:10.3390/vetsci12090881)
Supplement: Supplementary file 1 [file vetsci-12-00881-s001.zip › Supplementary Material_File_S2.pdf]

Supplementary Material File S2~~B~~

Scoping review of factors affecting antimicrobial use and the spread of antimicrobial resistance in the broiler production chain

Zsuzsa Farkas[1,2], Orsolya Strang[1,2*], Andrea Zentai[1,2], Szilveszter Csorba[1,2], Máté Farkas[1,2], András Bittsánszky[1,2], András Tóth[1,2], Miklós Süth[1,3], Ákos Józwiak[1,2]

[1]Institute of Food Chain Science, Department of Digital Food Science, University of Veterinary Medicine Budapest, Budapest, Hungary

[2]National Laboratory of Infectious Animal Diseases, Antimicrobial Resistance, Veterinary Public Health and Food Chain Safety, University of Veterinary Medicine Budapest, Budapest, Hungary

[3]Institute of Food Chain Science, University of Veterinary Medicine Budapest, Budapest, Hungary

*** Correspondence:**

Orsolya Strang

strang.orsolya@univet.hu

Note: The knowledge synthesis focused on the thematic discussion of factors influencing the increase or decrease of AMU and/or AMR. Consequently, it does not systematically distinguish in all cases whether these factors directly affect AMU or AMR. However, the separation of factors that directly impact AMR (and not indirectly via AMU) is deemed important, this is the reason that two separate tables present the detailed results (Table S1 and S2).

Table S1. Factors directly affecting AMR with target population and location indicated. Red, green and grey colors represent increase, decrease and no effect on AMR, respectively.

Category	Effect	Factor (Reference)	Target population	Country

**KAP (ON FARM): farm workers' health status**	**Increasing**	diarrhea of poultry worker in the last 3 months [1]	poultry, not specified	Nigeria
**KAP (ON FARM): farm workers' age**	**Increasing**	exposure to work for over 10 years [1]	poultry, not specified	Nigeria
**KAP (ON FARM) - farming practices: sewage management**	**Increasing**	sewage samples have high percentage of antimicrobial-resistant *E. coli* [2]	poultry, not specified further	Malaysia
**KAP (ON FARM) - farming practices: litter/manure management**	**Increasing**	turkeys placed on litter previously used by chickens [3]	chicken turkey (broiler turkey, heavy turkey)	Canada
		chicken litter harbors highest resistant bacterial strains [4]	chicken	India
		wood shaving as litter [5]	poultry, not specified	Brazil
		presence of antibiotic growth promoters in litter materials [5]	poultry, not specified	Brazil
		agricultural activities that involve use of animal manure [6]	poultry and domestic pig farming	Tanzania
	**Decreasing**	certain bedding materials (shredded straw) [7]	broiler	Belgium Bulgaria Denmark France Germany Italy Poland Spain

				the Netherlands
		storing manure on the farm [8]	broiler	Canada
		transfer (removal) of feces and carcasses associated with higher biosecurity scores [9]	broiler	Belgium Bulgaria Denmark France Germany Italy Poland Spain the Netherlands
		composting of manure [10]	broiler	France
**KAP (ON FARM) - farming practices: carcass management**	**Increasing**	disposing of dead chickens into the environment [11]	broiler	Senegal
	**Decreasing**	transfer (removal) of feces and carcasses associated with higher biosecurity scores [9]	broiler	Belgium Bulgaria Denmark France Germany Italy Poland Spain the Netherlands
**KAP (ON FARM) - farming practices: water management**	**Increasing**	use of river water for irrigation [6]	poultry and domestic pig farming	Tanzania
		farms using water from artesian wells [12]	broiler	Jordan
		using pump water/surface water [2]	poultry, not specified further	Malaysia
		meat vendors using the same	broiler	Malaysia

		water to wash hands and utensils [13]		
**KAP (ON FARM) - farming practices: flock/herd – arrangement**	**Increasing**	conventionally raised chickens – as opposed to free-range poultry [14]	chicken	Brazil
		deep litter system has a higher risk than the battery cage system [15]	broilers layers dual purpose	Uganda
		farms with at least two buildings housing chickens-not significantly associated [11]	broiler	Senegal
		non-practicing of an AIAO (all-in-all-out) system [16]	chicken, not specified	Vietnam
	**Decreasing**	free-range poultry production [14]	chicken	Brazil
		keeping livestock penned associated with better attitude [17]	poultry (and pig and aquaculture), not specified further	Vietnam
		all-in-all-out farming systems [18]	chicken	Vietnam
		higher weight of broilers at set-up associated with ARG abundance [9]	broiler	Belgium Bulgaria Denmark France Germany Italy Poland Spain

				the Netherlands
		average number of rounds per year associated with ARG abundance [9]	broiler	Belgium Bulgaria Denmark France Germany Italy Poland Spain the Netherlands
**KAP (ON FARM) - farming practices: flock/herd - size**	**Increasing**	flock size [19]	broiler chicken	Canada
		large flock size [20]	broiler chicken	India
**KAP (ON FARM) - farming practices: flock/herd – size**	**Increasing**	flocks of 700–1200 birds were at a higher risk [15]	broilers layers dual purpose	Uganda
		high density of chickens [16]	chicken, not specified	Vietnam
		stocking density negatively associated with practice [21]	poultry, not specified	Cameroon
**KAP (ON FARM) - farming practices: number of farm workers**	**Decreasing**	number of farm workers [9]	broiler	Belgium Bulgaria Denmark France Germany Italy Poland Spain the Netherlands
**KAP (ON FARM) - farming practices: location of farm**	**Increasing**	closely located farms [12]	broiler	Jordan
	**Increasing**	smaller farms (~1000 birds) [2]	poultry, not	Malaysia

**KAP (ON FARM) - farming practices: farm size**			specified further	
		small farm size (200-2000 chickens) [16]	chicken, not specified	Vietnam
**KAP (ON FARM) - farming practices: feeding practices**	**Increasing**	use of commercial feed (medicated with AM) [16]	chicken, not specified	Vietnam
	**Decreasing**	$ZnCl_2$ and $CuSO_4$ in growth medium reduces conjugative transfer of resistance plasmids of ESBL *E.coli* [22]	broiler	Norway
**KAP (ON FARM) - farming practices: breed selection**	**Increasing**	the lineage of the bird used in the flock (Cobb and the mixture of Cobb and Ross increased the odds for FQr *C. jejuni* versus Ross) [19]	broiler chicken	Canada
		type of commercial chicken (broiler birds have higher risk than layer and dual-purpose birds) [15]	broilers layers dual purpose	Uganda
		raising chickens for meat or mixed (meat and egg) purposes, but not solely for egg-laying purposes [16]	chicken	Vietnam
		fast-growing breed [23]	broiler	Spain

**KAP (ON FARM) - animal health management**	**Increasing**	high frequency of digestive tract disease [24]	broiler	Cameroon
**KAP (ON FARM) - hygiene and biosecurity**	**Increasing**	poor biosecurity practices [20]	broiler chicken	India
		poor disinfection practices [20]	broiler chicken	India
		poor workers' hygiene [20]	broiler chicken	India
		disposal of solid wastes from the household to the environment [6]	poultry and domestic pig farming	Tanzania
		uncontrolled disposal of human and veterinary drugs to the environment [6]	poultry and domestic pig farming	Tanzania
		wild birds having access to poultry houses [25]	broiler turkey	United Kingdom
		absence of specific shoes for staff [26]	broiler	Bangladesh
		poor biosecurity practices [27]	broiler, layer (and cattle and sheep and goat), not specified further	Ghana Kenya Zambia Zimbabwe
		absence of lavatory of poultry worker [1]	poultry, not specified	Nigeria
		lack of observance of an empty period of the flock house	broiler	Senegal

		between chicken batches [11]		
		positive status of the previous flock in the broiler house [28]	broiler	Norway
		use of hydrogen peroxide to disinfect water lines during the growing period [8]	broiler	Canada
		transport personnel entering the room where the broilers are raised [28]	broiler	Norway
		personal movement, vehicles and instruments (considered as vectors) [12]	broiler	Jordan
	**Decreasing**	higher biosecurity standards [7]	broiler	Belgium<br>Bulgaria<br>Denmark<br>France<br>Germany<br>Italy<br>Poland<br>Spain<br>the Netherlands
		masks provided for staff [25]	broiler<br>turkey	United Kingdom
		detailed areas dusted before wet cleaning [25]	broiler<br>turkey	United Kingdom
		feed hoppers cleaned and disinfected [25]	broiler<br>turkey	United Kingdom

		on-farm biosecurity measures, cleaning and disinfection [9]	broiler	Belgium Bulgaria Denmark France Germany Italy Poland Spain the Netherlands
		having a change shoes/boots practice on the farm [16]	chicken, not specified	Vietnam
		sanitation of the stall environment (significantly related to bacterial contamination) [13]	broiler	Malaysia
		controlling for wild birds [8]	broiler	Canada
		routines for disinfection of floor between production cycles [28]	broiler	Norway
	**No effect**	small to no effect using the ideal method for cleaning and disinfection [29]	broiler	Canada
		no effect of decontamination of egg shells [30]	broiler	Belgium France the Netherlands
**KAP (ON FARM) - financial motivation**	**Increasing**	purchase of day-old chickens from sources other than industrial hatchery companies (local	chicken, not specified	Vietnam

		hatcheries, markets, neighbors etc.) [16]		
**KAP (ON FARM) - allowing visitors/transport personnel**	**Increasing**	allowing visitors on farm [11]	broiler	Senegal
		visitors and farmworkers [9]	broiler	Belgium<br>Bulgaria<br>Denmark<br>France<br>Germany<br>Italy<br>Poland<br>Spain<br>the Netherlands
**KAP (ON FARM) - AMU practices (excluding intentional misuse)**	**Increasing**	AM usage in the animal production sector [31]	domestic (broiler chicken, turkey, and pig) and wild (wild boar, red fox, and rodents: forest mouse, field mouse and field vole) animals	Poland
		suggesting a link between antimicrobial usage patterns and AMR development [32]	broiler	Nigeria
		antimicrobial application may lead to the development of resistance in the	chicken<br>turkey<br>duck	Russia

		enterococci population [33]		
		antimicrobial administration [34]	chicken<br>broiler<br>chicken	USA
		ceftiofur, oxytetracycline, streptomycin administration [34]	chicken<br>broiler<br>chicken	USA
		higher antimicrobial exposure at flock or farm level is associated with more AMR [35]	broiler	Belgium<br>Bulgaria<br>Denmark<br>France<br>Germany<br>Italy<br>Poland<br>Spain<br>the Netherlands
		FQ use in the past [25]	broiler<br>turkey	United Kingdom
		enrofloxacin use [36]	broiler	South Korea
		use of ceftiofur at hatchery [3]	chicken<br>turkey<br>(broiler turkey, heavy turkey)	Canada
		tetracycline usage in turkey [3]	chicken<br>turkey<br>(broiler turkey, heavy turkey)	Canada
		bacitracin usage in chicken and turkey [3]	chicken<br>turkey<br>(broiler turkey, heavy turkey)	Canada

		gentamicin usage in turkey [3]	chicken turkey (broiler turkey, heavy turkey)	Canada
		tylosin usage in chicken [3]	chicken turkey (broiler turkey, heavy turkey)	Canada
**KAP (ON FARM) - farm workers' knowledge/attitude**	**Increasing**	carelessness among the livestock farmers [37]	chicken	Nepal
**KAP (ON FARM): farm workers' income**	**Increasing**	poor financial status [37]	chicken	Nepal
**KAP (ON FARM) - operational issues**	**Increasing**	single-handed operation of the site [25]	broiler turkey	United Kingdom
		the existence of a public footpath on the periphery of the site [25]	broiler turkey	United Kingdom
	**Decreasing**	the site operated by an independent grower than large company [25]	broiler turkey	United Kingdom
**INTENTIONAL MISUSE/BAD PRACTICES OF ABU - other/general**	**Increasing**	use of AB without prescription of veterinarians [26]	broiler	Bangladesh
		no guidelines regarding the sale of AM [37]	chicken	Nepal
		irrational sales of AM [37]	chicken	Nepal
		availability of falsified/substandard drugs [37]	chicken	Nepal

**ENVIRONMENTAL FACTORS - soil (minerals)**	**Decreasing**	<300 ppm Mg in soil recommended to reduce MDR *Listeria* incidence [38]	broiler	USA
		≤5000 ppm P in feces recommended for reduction of MDR *Listeria* incidence [38]	broiler	USA
		7000–12,000 ppm K in feces recommended for reduction of MDR *Listeria* incidence [38]	broiler	USA
		>200 ppm K in soil recommended for reduction of MDR *Listeria* incidence [38]	broiler	USA
		>15 C:N in soil recommended for reduction of MDR *Listeria* incidence [38]	broiler	USA
		>18 ppm Cu in feces recommended for reduction of MDR *Listeria* incidence [38]	broiler	USA
		>3 ppm Cr in feces recommended for reduction of MDR *Listeria* incidence [38]	broiler	USA
		6–15 years farming for soil	broiler	USA

		recommended for reduction of MDR *Listeria* incidence [38]		
		≥2000 ppm Mg in feces recommended for reduction of MDR *Salmonella* incidence [38]	broiler	USA
		≤300 ppm Mg in soil recommended for reduction of MDR *Salmonella* incidence [38]	broiler	USA
		≤150 ppm P in soil recommended for reduction of MDR *Salmonella* incidence [38]	broiler	USA
		≤2000 microS/cm electrical conductivity of feces recommended for reduction of MDR *Salmonella* incidence [38]	broiler	USA
		≤6.5 pH in feces recommended for reduction of MDR *Salmonella* incidence [38]	broiler	USA
		≥70 ppm Mn in soil recommended for reduction of MDR *Salmonella* incidence [38]	broiler	USA
		<50 ppm Na in soil recommended for	broiler	USA

		reduction of MDR *Salmonella* incidence [38]		
**ENVIRONMENTAL FACTORS – seasonality**	**Increasing**	winter season (birds gather together for heat allowing to increase stocking density thus the spread of bacterial infection) [26]	broiler	Bangladesh
	**Decreasing**	summer season [7]	broiler	Belgium<br>Bulgaria<br>Denmark<br>France<br>Germany<br>Italy<br>Poland<br>Spain<br>the Netherlands
**ENVIRONMENTAL FACTORS - geographical location**	**Increasing**	birds from Wakiso Town Council have higher risk [15]	broilers<br>layers<br>dual purpose	Uganda
**VECTORS - Living vectors: invertebrate**	**Increasing**	fly-mediated transmission of mcr-1 from animals and the environment to humans [39]	poultry (and swine)	Vietnam
**VECTORS - Living vectors: vertebrate**	**Increasing**	the potential of wildlife as AMR transmission vectors [31]	domestic (broiler chicken, turkey, and pig) and wild (wild boar, red fox, and rodents: forest mouse,	Poland

			field mouse and field vole) animals	
		strongly suggest the possibility of vertical transmission from parent flocks [40]	Ross male chickens – broiler	Spain
		humans and/or wildlife (pests, wild birds) involvement because fluoroquinolones are excluded from use in Australian livestock [41]	meat chickens	Australia
**VECTORS - Living vectors: bacterial/bacteriophage (horizontal gene transfer)**	**Increasing**	plasmids transferred by conjugation [10]	broiler	France
		horizontal gene transfer from commensal to susceptible pathogen [42]	broiler	USA
		in vitro transduction by phages from migratory wild birds to poultry *E. coli* isolates [43]	chicken, turkey, duck, not specified further	Mexico
**VECTORS - Inanimate vectors**	**Increasing**	broiler hatching eggs as carriers and a potential source of ESBL/AmpC-producing *Enterobacteriaceae* for broiler chicks [30]	broiler	Belgium France the Netherlands

**AGE OF THE ANIMALS**	**Increasing**	birds aged 4 months and below have higher risk [15]	broilers layers dual purpose	Uganda
		age of birds (≥ 11 days) [44]	broiler	Bangladesh
**VETERINARIANS/ HEALTH CARE WORKERS - knowledge/education/ experience**	**Increasing**	lack of training among veterinary personnel [37]	chicken	Nepal

Table S2. Factors affecting AMU with target population and location indicated. Red, green and grey represent increase, decrease and no effect on AMR, respectively

**Category**	**Effect**	**Factor (Reference)**	**Target population**	**Country**
**KAP (ON FARM) – farm workers' knowledge/attitude**	**Increasing**	lack of general understanding and awareness [45]	livestock farmers and managers (dairy, beef, broiler, and layer)	Fiji
		lack of familiarity with the term of AB [46]	livestock producers (chicken, guinea pig, pig, rabbit, and other animals included duck, quail, cow, horse, and sheep)	Ecuador
		personal beliefs on AMR [47]	farm animal (i.e., poultry, swine, veal calves and dairy) veterinarians	the Netherland s

		inadequate knowledge on infection prevention and control of animal diseases [6]	poultry and domestic pig farming	Tanzania
	**Decreasing**	inadequate knowledge on infection prevention and control of animal diseases [6]	poultry and domestic pig farming	Tanzania
		knowledge of the animal health authority [48]	chicken (and cow and pig), not specified further	Peru
		knowledge of an antiparasitic drug [48]	chicken (and cow and pig), not specified further	Peru
**KAP (ON FARM) – farm workers' education/training**	**Increasing**	level of education associated with frequency of using AM [6]	poultry and domestic pig farming	Tanzania
		level of education associated with performing group treatment [6]	poultry and domestic pig farming	Tanzania
		high level of education negatively influenced knowledge [49]	broiler, layer, not specified further	Burkina Faso

		higher education associated with AB use for prevention and growth promotion [49]	broiler, layer, not specified further	Burkina Faso
		low education and expertise [50]	commercial poultry - exotic birds local birds	Nigeria
		farms with reported veterinary skills had a greater occurrence of resistant isolates [11]	broiler	Senegal
		trained staff potentially associated with the presence of resistant *E.coli* on the farm [11]	broiler	Senegal
		farm training on antimicrobial use and AMR associated with less prudent practice [27]	broiler	Ghana<br>Kenya<br>Zambia<br>Zimbabwe
		lack of training in farming associated with MDR *E.coli* [24]	broiler	Cameroon
	**Decreasing**	high school education associated with asking for	laying hen<br>broiler	Vietnam

		advice before using AM [51]		
		high school education associated with complying with withdrawal times before slaughtering [51]	laying hen broiler	Vietnam
		educational level of farmer associated with degree of knowledge of AB [48]	chicken (and cow and pig), not specified further	Peru
		educational level of farmer positively associated with KAPP score [21]	poultry, not specified	Cameroon
		education of farmer (bachelor or higher) associated with better attitude [52]	layer, broiler	Bangladesh
		college or university degree associated with diagnostic tests before using AM [51]	laying hen broiler	Vietnam
		higher education (graduate level) associated	broiler, layer	Bangladesh

		with correct KAP [53]		
		higher education associated with better knowledge and attitude but with similar practices as with less education [17]	poultry (and pig and aquaculture), not specified further	Vietnam
		level of education significantly related to the possibility of reducing AM use [6]	poultry and domestic pig farming	Tanzania
		high school education and college or university degree associated with using more than one AM in a treatment therapy [51]	laying hen broiler	Vietnam
		higher education associated with using a qualified veterinarian [49]	broiler, layer, not specified further	Burkina Faso
		education (tertiary/post-secondary) and educational specialization	broiler	Nigeria

		(agriculture/veterinary-oriented post-secondary educational specialization) associated with adequate knowledge, attitude and risk perception [32]		
		training in poultry husbandry associated with higher knowledge on AMU [21]	poultry, not specified	Cameroon
		farm training associated with knowledge and more prudent attitudes [27]	broiler	Ghana<br>Kenya<br>Zambia<br>Zimbabwe
**KAP (ON FARM) – farm workers' experience**	**Increasing**	experience in poultry farming negatively associated with practice [21]	poultry, not specified	Cameroon
	**Decreasing**	farming experience has a significant relationship with the prudent use of AM and the proper sourcing of the AM [6]	poultry and domestic pig farming	Tanzania

		experience in poultry farming [21]	broiler	Cameroon
		years keeping poultry positively associated with KAP [27]	broiler	Ghana Kenya Zambia Zimbabwe
		more (9-12 years) farming experience associated with correct KAP on AMU, AMR [53]	broiler, layer	Bangladesh
		less experience associated with satisfactory attitude [32]	broiler	Nigeria
**KAP (ON FARM): farm workers' age**	**Increasing**	farmers less than 20-year-old [54]	chicken (broilers and layers) farms, pig farms, fish farms (fishponds and/or fish-cages) and livestock-fish farms corresponding to pig–fish and poultry (layer, broiler or duck)-fish farms	Lao PDR
		age of farmer negatively associated with knowledge, practice, risk perception [21]	poultry, not specified	Cameroon

	**Decreasing**	farmers more than 51-year-old [54]	chicken (broilers and layers) farms, pig farms, fish farms (fishponds and/or fish-cages) and livestock-fish farms correspondin g to pig–fish and poultry (layer, broiler or duck)-fish farms	Lao PDR
		greater age of farmer [55]	chicken, duck (and pig), not specified further	Vietnam
		older poultry farmers associated with correct KAP [53]	broiler, layer	Bangladesh
		age of farmer positively associated with KAP [27]	broiler	Ghana<br>Kenya<br>Zambia<br>Zimbabwe
		lower age of farmer associated with satisfactory attitude [32]	broiler	Nigeria
**KAP (ON FARM): farm workers' gender**	**Increasing**	farms run by a male farmer associated with higher level of AM usage [18]	chicken	Vietnam
		gender of farmer (female)	poultry, not specified	Cameroon

		negatively associated with practice [21]		
**KAP (ON FARM): farm workers' income**	**Increasing**	weak financial status of poultry owners [50]	commercial poultry - exotic birds local birds	Nigeria
		livelihood precarity [56]	farmers (broiler, layer, goat, pig), veterinary prescribers and dispensers, stakeholders	Malawi
	**Decreasing**	monthly income of farmer positively correlated with degree of knowledge of AB [48]	chicken (and cow and pig), not specified further	Peru
		higher income of farmer positively associated with correct KAP [53]	broiler, layer	Bangladesh
**KAP (ON FARM): farm workers' marital status**	**Decreasing**	single and separated marital status of farmer associated with knowledge, attitude and risk perception regarding AMU and AMR [32]	broiler	Nigeria

**KAP (ON FARM): farm workers' location**	**Decreasing**	locality of farmer associated with knowledge on AMU and AMR [21]	poultry, not specified	Cameroon
**KAP (ON FARM) - farming practices: flock/herd – arrangement**	**Increasing**	animals are kept in cramped space within the farmer home [56]	farmers (broiler, layer, goat, pig), veterinary prescribers and dispensers, stakeholders	Malawi
		animal confinement [57]	cattle and poultry veterinarians	Ecuador
	**Decreasing**	free-range local bird flocks rarely used AM [50]	commercial poultry - exotic birds local birds	Nigeria
		growth duration/cycle and higher broiler stocking batch associated with adequate knowledge, attitude and risk perception regarding AMU and AMR [32]	broiler	Nigeria
**KAP (ON FARM) - farming practices: flock/herd - size**	**Increasing**	poultry herd sizes of fewer than 5,000 hens (underdosage) [58]	laying hen	Cameroon

		poultry herd sizes between 5,000 and 10,000 hens (overdosage) [58]	laying hen	Cameroon
		independent farms with larger flocks increasingly use AM [59]	broiler	Indonesia
**KAP (ON FARM) - farming practices: flock/herd – density**	**Increasing**	densities <7/m2 (overdosage) [58]	laying hen	Cameroon
		area densely populated with poultry positively associated with AM consumption [60]	broiler	Italy
**KAP (ON FARM) - farming practices: number of farm workers**	**Increasing**	livestock ratio of fewer than 2,000 hens per employee (overdosage) [58]	laying hen	Cameroon
**KAP (ON FARM) - farming practices: location of farm**	**Increasing**	neighboring poultry and/or swine backyard production system (BPS) [61]	backyard production systems maintaining hen, duck, swine, goose, sheep, goat, dairy cattle, horse, rabbit, cat and dog	Chile
**KAP (ON FARM) - farming practices: farm size**	**Increasing**	small-scale commercial poultry farms to frequently used AM	commercial poultry - exotic birds local birds	Nigeria

		without veterinarians' consultations [50]		
		high dependence on AB for small-scale intensive farming [56]	farmers (broiler, layer, goat, pig), veterinary prescribers and dispensers, stakeholders	Malawi
		backyard production systems (BPS) size (smaller BPS has more chances of using AM) [61]	backyard production systems maintaining hen, duck, swine, goose, sheep, goat, dairy cattle, horse, rabbit, cat and dog	Chile
	**Decreasing**	proportion of industrial poultry farms used AM was significantly higher than household farms [51]	laying hen broiler	Vietnam
		consultation rates before using AM in industrial farm was significantly higher than household farms [51]	laying hen broiler	Vietnam
		chicken farmers which used diagnostic tests before	laying hen broiler	Vietnam

		using AM was significantly more likely to be semi-industrial and industrial farms [51]		
		compliance with the manufacturer's recommended dosage on semi-industrial farms and industrial farms was significantly higher compared to household farms [51]	laying hen broiler	Vietnam
		compliance on industrial chicken farms was significantly higher compared to households [51]	laying hen broiler	Vietnam
		farm area positively correlated with degree of knowledge of AB [48]	chicken (and cow and pig), not specified further	Peru
		size of the farm associated with good practice: consulting a qualified veterinarian in case of disease	broiler, layer, not specified further	Burkina Faso

		outbreaks and buying veterinary drugs in formal drug stores [49]		
		medium farm size was more associated with correct KAP than small [53]	broiler, layer	Bangladesh
		smaller scale farming was more associated with better attitude [17]	poultry (and pig and aquaculture), not specified further	Vietnam
**KAP (ON FARM) - farming practices: feeding practices**	**Increasing**	antibiotics in food and vitamin mixes [56]	farmers (broiler, layer, goat, pig), veterinary prescribers and dispensers, stakeholders	Malawi
		feed source (self-compounding milled at a feed mill and usage of finished commercial feeds) negatively associated with knowledge, attitude and risk perception regarding AMU and AMR [32]	broiler	Nigeria

		low feed quality [57]	cattle and poultry veterinarians	Ecuador
**KAP (ON FARM) - farming practices: breed selection**	**Increasing**	meat chicken farms [18]	chicken	Vietnam
**KAP (ON FARM) - farming practices: administration/record keeping**	**Increasing**	poor record keeping associated with inadequate knowledge of AMU [52]	layer, broiler	Bangladesh
	**Decreasing**	keeping records is associated with AMR knowledge [27]	broiler	Ghana<br>Kenya<br>Zambia<br>Zimbabwe
		keeping records of AB use is associated with better attitude [17]	poultry (and pig and aquaculture), not specified further	Vietnam
**KAP (ON FARM) - farming practices: species per farm**	**Increasing**	specialized farms (one species only) [54]	chicken (broilers and layers) farms, pig farms, fish farms (fishponds and/or fish-cages) and livestock-fish farms correspondin g to pig–fish and poultry (layer, broiler or duck)-fish farms	Lao PDR
		close contact between	backyard production systems maintaining	Chile

		animal species in the BPS [61]	hen, duck, swine, goose, sheep, goat, dairy cattle, horse, rabbit, cat and dog	
	**Decreasing**	presence of pets in backyard production systems [61]	backyard production systems maintaining hen, duck, swine, goose, sheep, goat, dairy cattle, horse, rabbit, cat and dog	Chile
**KAP (ON FARM) - animal health management**	**Increasing**	recognition of diseases in animals [61]	backyard production systems maintaining hen, duck, swine, goose, sheep, goat, dairy cattle, horse, rabbit, cat and dog	Chile
		inadequate consultation of animal care personnel associated with lower knowledge on AMU [21]	poultry, not specified	Cameroon
		professionals often perceived as the "last resorts" for animal health and agrovet employees often the first sources of health advice	broiler, layer (and cattle and sheep and goat), not specified further	Ghana<br>Kenya<br>Zambia<br>Zimbabwe

		sought by farmers [27]		
		adding minerals, diuretics with AB [21]	poultry, not specified	Cameroon
		method used for disease diagnosis (misdiagnosis, over/under-dosing because of diagnosis based on clinical symptoms) [6]	poultry and domestic pig farming	Tanzania
		easy access to AB [6]	poultry and domestic pig farming	Tanzania
		home storage of veterinary AM [6]	poultry and domestic pig farming	Tanzania
		group treatment [6]	poultry and domestic pig farming	Tanzania
	**Decreasing**	disease management (negatively associated) [9]	broiler	Belgium Bulgaria Denmark France Germany Italy Poland Spain the Netherlands
**KAP (ON FARM) - hygiene and biosecurity**	**Increasing**	poor biosecurity measures [57]	cattle and poultry veterinarians	Ecuador
		insufficient farm biosecurity [51]	laying hen broiler	Vietnam

	**Decreasing**	biosecurity measures positively associated with AMU and risk perception of AMR [21]	poultry, not specified	Cameroon
**KAP (ON FARM) - financial motivation**	**Increasing**	unequal market conditions [56]	farmers (broiler, layer, goat, pig), veterinary prescribers and dispensers, stakeholders	Malawi
		misuse of antimicrobials to shorten the period of farming [6]	poultry and domestic pig farming	Tanzania
		economic considerations prevent the investment in biosecurity [27]	broiler, layer (and cattle and sheep and goat), not specified further	Ghana Kenya Zambia Zimbabwe
		economic advantage, low relative cost of antimicrobials [59]	broiler	Indonesia
		economic aspects hindering disease preventive actions [47]	farm animal (i.e., poultry, swine, veal calves and dairy) veterinarians	the Netherlands
		livestock production as primary source of income [45]	livestock farmers and managers (dairy, beef,	Fiji

			broiler, and layer)	
		cost of veterinary advice and AB often resulted underdosed or slaughtered sick animal [56]	farmers (broiler, layer, goat, pig), veterinary prescribers and dispensers, stakeholders	Malawi
		giving antibiotics to guarantee health of livestock and ensuring profit [17]	poultry (and pig and aquaculture), not specified further	Vietnam
		farmer's economic condition influences prescription [62]	poultry (and livestock), not specified further	Bangladesh
		farmers do not call a veterinarian due to economic reasons [57]	cattle and poultry veterinarians	Ecuador
		inherited livestock farms [45]	livestock farmers and managers (dairy, beef, broiler, and layer)	Fiji
**KAP (ON FARM) - AMU practices (excluding intentional misuse): conventional vs. antibiotic free farms**	**No effect**	no difference between antimicrobial-free and conventional farms regarding resistance [29]	broiler	Canada

**KAP (ON FARM) - AMU practices (excluding intentional misuse): type of administration**	**Increasing**	AMs administration through drinking water [50]	commercial poultry - exotic birds local birds	Nigeria
		in ovo use of ceftiofur [8]	broiler	Canada
**KAP (ON FARM) - AMU practices (excluding intentional misuse): proper use of different ABs**	**Increasing**	antimicrobial use [63]	broiler	Vietnam
		antimicrobial use [9]	broiler	Belgium Bulgaria Denmark France Germany Italy Poland Spain the Netherlands
		suggesting a link between antimicrobial usage patterns and AMR development [32]	broiler	Nigeria
		quinolone and tetracycline usage factors for ciprofloxacin resistance in *E.coli*, lincosamide and tetracycline usage factors for gentamicin resistance in *E.coli*, usage of any antimicrobial drug is factor	chicken, not specified	Vietnam

		for 3rd generation cephalosporins resistance in *E. coli*, tetracycline usage is factor for quinolone resistance in *E. coli* [16]		
		withdrawal of individual compounds, such as cephalosporins and fluoroquinolones increased use and resistance level of other drug classes, such as aminoglycosides [29]	broiler	Canada
	**No effect**	colistin administration [10]	broiler	France
**KAP (ON FARM) - AMU practices (excluding intentional misuse)**	**Increasing**	suggesting a link between antimicrobial usage patterns and AMR development [64]	broiler	Nigeria
**KAP (ON FARM) - operational issues**	**Increasing**	intensive husbandry management system [50]	commercial poultry - exotic birds local birds	Nigeria
		farmers' management practices [47]	farm animal (i.e., poultry, swine, veal calves and	the Netherlands

			dairy) veterinarians	
		sales target of broiler grow-out farmers (company-owned production better than contractual) [32]	broiler	Nigeria
**INTENTIONAL MISUSE/BAD PRACTICES OF ABU – prophylactic use**	**Increasing**	disease prevention [51]	laying hen broiler	Vietnam
		prophylactic purposes [6]	poultry and domestic pig farming	Tanzania
		frequent prophylactic and growth promoting usage of antibiotics [65]	broiler layer Sonali (a locally produced cross-bred between Rhode Island Red male and Fayoumi female, reared for both meat and egg production)	Bangladesh
		prophylactic use [49]	broiler, layer	Burkina Faso
		prophylactic use [17]	chicken	Vietnam
		prophylactic use [21]	poultry, not specified	Cameroon
		prophylactic use [27]	broiler, layer (and cattle and sheep and goat), not specified further	Ghana Kenya Zambia Zimbabwe

		antimicrobials used for therapeutic, prophylactic, and growth promotion [50]	commercial poultry - exotic birds local birds	Nigeria
		prescribing AB for prophylaxis [62]	poultry (and livestock), not specified further	Bangladesh
		antimicrobials used as feed additives [24]	broiler	Cameroon
		contract farmers using AB for prevention and based on advice from a contract company [59]	broiler	Indonesia
		metaphylactic and prophylactic treatments with underdosing or overdosing [58]	laying hen	Cameroon
		antibiotics should be used for prophylactic treatment [54]	chicken (broilers and layers) farms, pig farms, fish farms (fishponds and/or fish-cages) and livestock-fish farms corresponding to pig–fish and poultry (layer, broiler	Lao PDR

			or duck)-fish farms	
**INTENTIONAL MISUSE/BAD PRACTICES OF ABU – incomplete doses**	**Increasing**	giving only a single dose of AMs once on sick birds [50]	commercial poultry - exotic birds local birds	Nigeria
		improper antimicrobial dosage [50]	commercial poultry - exotic birds local birds	Nigeria
		stopping antibiotic treatment [57]	cattle and poultry veterinarians	Ecuador
		incomplete doses associated with inadequate knowledge on AMU [52]	layer, broiler	Bangladesh
		stopping the treatment when clinical signs disappear [48]	chicken (and cow and pig)	Peru
**INTENTIONAL MISUSE/BAD PRACTICES OF ABU - other/general**	**Increasing**	AB misuse [46]	livestock producers (chicken, guinea pig, pig, rabbit, and other animals included duck, quail, cow, horse, and sheep)	Ecuador
		self-administration of AM [50]	commercial poultry - exotic birds local birds	Nigeria
		use for treatment and prevention	broiler, laying hens, roosters	

		based on their own experience [66]	exotic breed chickens crossbreed chickens (mix between a local and an exotic breed) local breed chickens	
		buying AB in veterinary supply stores [57]	cattle and poultry veterinarians	Ecuador
		nonenforcement of AMU regulating laws in poultry [50]	commercial poultry - exotic birds local birds	Nigeria
		medicine shortage resulted in hoarding medicine and self-prescribing [45]	livestock farmers and managers (dairy, beef, broiler, and layer)	Fiji
		unrestricted access to AB [56]	farmers (broiler, layer, goat, pig), veterinary prescribers and dispensers, stakeholders	Malawi
		using veterinary drugs without prescriptions [49]	broiler, layer, not specified further	Burkina Faso
		basing drug decisions off symptom descriptions or	broiler, layer (and cattle and sheep and goat),	Ghana Kenya Zambia Zimbabwe

		specific requests of the farmer [27]	not specified further	
		no influence from culture and susceptibility tests and patients' AMU history when prescribing antibiotics [62]	poultry (and livestock), not specified further	Bangladesh
		prescribing AM through telephone conversations [62]	poultry (and livestock), not specified further	Bangladesh
		unprofessional practitioners [62]	poultry (and livestock), not specified further	Bangladesh
		agrovet dealers selling AB without prescription [27]	broiler, layer (and cattle and sheep and goat), not specified further	Ghana Kenya Zambia Zimbabwe
		buying unregulated medicine from black markets [2]	poultry, not specified further	Malaysia
		infrequent use of AB to treat parasites or animals not eating [48]	chicken (and cow and pig), not specified further	Peru
		improper use of AM as painkiller and treatment of viral diseases [21]	poultry, not specified	Cameroon

		easy access to cheap over-the-counter AM [51]	laying hen<br>broiler	Vietnam
		use of human AM in animals [6]	poultry and domestic pig farming	Tanzania
		no differentiation between AM and other types of medicine [45]	livestock farmers and managers (dairy, beef, broiler, and layer)	Fiji
		irrational and inappropriate use of AB in commercial chicken production can contribute to the development of AMR [65]	broiler<br>layer<br>Sonali (a locally produced cross-bred between Rhode Island Red male and Fayoumi female, reared for both meat and egg production)	Bangladesh
		over use and/or improper AMU [67]	veterinarians (cattle, buffalo, sheep, goat, poultry, pig)	India
		weight gain [51]	laying hen<br>broiler	Vietnam
		noncompliance with AMs withdrawal periods [50]	commercial poultry - exotic birds local birds	Nigeria
		storing AB on farms [57]	cattle and poultry veterinarians	Ecuador

		stocking of AM [6]	poultry and domestic pig farming	Tanzania
		administering AB at the first indication of disease [17]	poultry (and pig and aquaculture), not specified further	Vietnam
		using AM for faster and bigger growth [27]	broiler, layer (and cattle and sheep and goat), not specified further	Ghana Kenya Zambia Zimbabwe
		constant use of broad-spectrum AB more frequently [68]	livestock (cattle, pig, poultry, sheep, goat)	Uganda
		no observation of withdrawal periods [27]	broiler, layer (and cattle and sheep and goat), not specified further	Ghana Kenya Zambia Zimbabwe
		not respecting withdrawal periods [49]	broiler, layer, not specified further	Burkina Faso
		administration of AM to one-day old-chicks for preventive purposes [21]	poultry, not specified	Cameroon
		use of AM in the last 1.73 weeks (estimated parameter) before slaughter [63]	broiler	Vietnam
		poor handling of drugs at purchase and	livestock (cattle, pig,	Uganda

		administration practices [68]	poultry, sheep, goat)	
		low enforcement of policies and regulations [68]	livestock (cattle, pig, poultry, sheep, goat)	Uganda
**ENVIRONMENTAL FACTORS - seasonality**	**Increasing**	season (winter/spring) is significantly associated with AMU [60]	broiler	Italy
**ENVIRONMENTAL FACTORS - geographical location**	**Increasing**	backyard production systems (BPS) located closer to 0 meters above sea level have more chances of using AM [61]	backyard production systems maintaining hen, duck, swine, goose, sheep, goat, dairy cattle, horse, rabbit, cat and dog	Chile
		geographical location (Southern Italy) and elevation significantly associated with AMU [60]	broiler	Italy
**HEALTH STATUS OF THE ANIMALS**	**Decreasing**	no(t known) disease occurred in the past 12 months [54]	chicken (broilers and layers) farms, pig farms, fish farms (fishponds and/or fish-cages) and livestock-fish farms correspondin g to pig–fish and poultry (layer, broiler	Lao PDR

			or duck)-fish farms	
**VETERINARIANS/HEALTH CARE WORKERS - practices**	**Increasing**	perceptions of professional responsibilities [47]	farm animal (i.e., poultry, swine, veal calves and dairy) veterinarians	the Netherlands
		risk avoidance [47]	farm animal (i.e., poultry, swine, veal calves and dairy) veterinarians	the Netherlands
**VETERINARIANS/HEALTH CARE WORKERS - knowledge/education/ experience**	**Increasing**	inadequate veterinary extension officers [6]	poultry and domestic pig farming	Tanzania
		lack of compliance to veterinary advices [47]	farm animal (i.e., poultry, swine, veal calves and dairy) veterinarians	the Netherlands
		shortcomings in advisory competencies of veterinarians [47]	farm animal (i.e., poultry, swine, veal calves and dairy) veterinarians	the Netherlands
	**Decreasing**	level of education of veterinarian, previous training on AMU and AMR, and years of experience associated with KAP on AMU [62]	poultry (and livestock), not specified further	Bangladesh

**VETERINARIANS/HEALTH CARE WORKERS - age**	**Decreasing**	age associated with increased KAP on AMU [62]	poultry (and livestock), not specified further	Bangladesh
**VETERINARIANS/HEALTH CARE WORKERS - other**	**Increasing**	financial dependency on clients [47]	farm animal (i.e., poultry, swine, veal calves and dairy) veterinarians	the Netherlands
		client pressure [47]	farm animal (i.e., poultry, swine, veal calves and dairy) veterinarians	the Netherlands
		limited diagnostic facilities [68]	livestock (cattle, pig, poultry, sheep, goat)	Uganda
		difficult for farmers to access veterinary advice on responsible antimicrobial use [59]	broiler	Indonesia
		input from animal health professionals is rare (difficulties in accessing health professionals) [27]	broiler, layer (and cattle and sheep and goat), not specified further	Ghana Kenya Zambia Zimbabwe
		professionals often perceived as the "last resorts" for animal health [27]	broiler, layer (and cattle and sheep and goat), not specified further	Ghana Kenya Zambia Zimbabwe

		animal health professionals lacked the resources to provide proper services [27]	broiler, layer (and cattle and sheep and goat), not specified further	Ghana Kenya Zambia Zimbabwe
		lack of access to veterinary services [51]	laying hen broiler	Vietnam
**DRUG/FEED SELLERS knowledge**	**Increasing**	sales agents' roles as non-professional prescribers of antibiotics [57]	cattle and poultry veterinarians	Ecuador
		knowledge gap of feed sellers and drug sellers, and farmers are dependent on sellers [53]	broiler, layer	Bangladesh
		low level of education of supply chain actors, particularly drug retailers [68]	livestock (cattle, pig, poultry, sheep, goat)	Uganda
		lack of awareness of stakeholders about policies that regulate drug use [68]	livestock (cattle, pig, poultry, sheep, goat)	Uganda
	**Decreasing**	years of experience [69]	poultry, not specified	Bangladesh
		level of education (up to 12th grade) [69]	poultry, not specified	Bangladesh
		training on the drug [69]	poultry, not specified	Bangladesh

		age of seller (younger) (positively) associated with KAP of drug and feed sellers [69]	poultry, not specified	Bangladesh

References:

1. Aworh, M.K.; Kwaga, J.; Okolocha, E.; Mba, N.; Thakur, S. Prevalence and Risk Factors for Multi-Drug Resistant Escherichia Coli among Poultry Workers in the Federal Capital Territory, Abuja, Nigeria. *PLoS ONE* **2019**, *14*, doi:10.1371/journal.pone.0225379.
2. Elmi, S.A.; Simons, D.; Elton, L.; Haider, N.; Hamid, M.M.A.; Shuaib, Y.A.; Khan, M.A.; Othman, I.; Kock, R.; Osman, A.Y. Identification of Risk Factors Associated with Resistant Escherichia Coli Isolates from Poultry Farms in the East Coast of Peninsular Malaysia: A Cross Sectional Study. *Antibiotics* **2021**, *10*, 1–17, doi:10.3390/antibiotics10020117.
3. Boulianne, M.; Arsenault, J.; Daignault, D.; Archambault, M.; Letellier, A.; Dutil, L. Drug Use and Antimicrobial Resistance among Escherichia Coli and Enterococcus Spp. Isolates from Chicken and Turkey Flocks Slaughtered in Quebec, Canada. *Canadian Journal of Veterinary Research* **2016**, *80*, 49–59.
4. Ashwini, A.; Jamwal, P.; Vanak, A.T. Environmental Surveillance of Antimicrobial Resistance in a Rapidly Developing Catchment. *Environmental Monitoring and Assessment* **2023**, *195*, doi:10.1007/s10661-022-10630-7.
5. Saraiva, M.M.S.; Silva, N.M.V.; Ferreira, V.A.; Moreira Filho, A.L.B.; Givisiez, P.E.N.; Freitas Neto, O.C.; Berchieri Júnior, A.; Gebreyes, W.A.; de Oliveira, C.J.B. Residual Concentrations of Antimicrobial Growth Promoters in Poultry Litter Favour Plasmid Conjugation among Escherichia Coli. *Letters in Applied Microbiology* **2022**, *74*, 831–838, doi:10.1111/lam.13671.
6. Kimera, Z.I.; Frumence, G.; Mboera, L.E.G.; Rweyemamu, M.; Mshana, S.E.; Matee, M.I.N. Assessment of Drivers of Antimicrobial Use and Resistance in Poultry and Domestic Pig Farming in the Msimbazi River Basin in Tanzania. *Antibiotics* **2020**, *9*, 1–20, doi:10.3390/antibiotics9120838.
7. Luiken, R.E.; Heederik, D.J.; Scherpenisse, P.; Van Gompel, L.; van Heijnsbergen, E.; Greve, G.D.; Jongerius-Gortemaker, B.G.; Tersteeg-Zijderveld, M.H.; Fischer, J.; Juraschek, K.; et al. Determinants for Antimicrobial Resistance Genes in Farm Dust on 333 Poultry and Pig Farms in Nine European Countries. *Environmental Research* **2022**, *208*, doi:10.1016/j.envres.2022.112715.
8. Caffrey, N.; Nekouei, O.; Gow, S.; Agunos, A.; Checkley, S. Risk Factors Associated with the A2C Resistance Pattern among E. Coli Isolates from Broiler Flocks in Canada. *Preventive Veterinary Medicine* **2017**, *148*, 115–120, doi:10.1016/j.prevetmed.2017.11.001.
9. Yang, D.; Heederik, D.J.J.; Mevius, D.J.; Scherpenisse, P.; Luiken, R.E.C.; Van Gompel, L.; Skarżyńska, M.; Wadepohl, K.; Chauvin, C.; Van Heijnsbergen, E.; et al. Risk Factors for the Abundance of Antimicrobial Resistance Genes Aph(3′)-III, Erm(B), Sul2 and

Tet(W) in Pig and Broiler Faeces in Nine European Countries. *Journal of Antimicrobial Chemotherapy* **2022**, *77*, 969–978, doi:10.1093/jac/dkac002.
10. Le Devendec, L.; Mourand, G.; Bougeard, S.; Léaustic, J.; Jouy, E.; Keita, A.; Couet, W.; Rousset, N.; Kempf, I. Impact of Colistin Sulfate Treatment of Broilers on the Presence of Resistant Bacteria and Resistance Genes in Stored or Composted Manure. *Veterinary Microbiology* **2016**, *194*, 98–106, doi:10.1016/j.vetmic.2015.11.012.
11. Vounba, P.; Arsenault, J.; Bada-Alambédji, R.; Fairbrother, J.M. Prevalence of Antimicrobial Resistance and Potential Pathogenicity, and Possible Spread of Third Generation Cephalosporin Resistance, in Escherichia Coli Isolated from Healthy Chicken Farms in the Region of Dakar, Senegal. *PLoS ONE* **2019**, *14*, doi:10.1371/journal.pone.0214304.
12. Ibrahim, R.A.; Cryer, T.L.; Lafi, S.Q.; Basha, E.-A.; Good, L.; Tarazi, Y.H. Identification of Escherichia Coli from Broiler Chickens in Jordan, Their Antimicrobial Resistance, Gene Characterization and the Associated Risk Factors. *BMC Veterinary Research* **2019**, *15*, doi:10.1186/s12917-019-1901-1.
13. Aliyu, A.B.; Saleha, A.A.; Jalila, A.; Zunita, Z. Risk Factors and Spatial Distribution of Extended Spectrum β-Lactamase-Producing- Escherichia Coli at Retail Poultry Meat Markets in Malaysia: A Cross-Sectional Study. *BMC Public Health* **2016**, *16*, doi:10.1186/s12889-016-3377-2.
14. Koga, V.L.; Scandorieiro, S.; Vespero, E.C.; Oba, A.; De Brito, B.G.; De Brito, K.C.T.; Nakazato, G.; Kobayashi, R.K.T. Comparison of Antibiotic Resistance and Virulence Factors among Escherichia Coli Isolated from Conventional and Free-Range Poultry. *BioMed Research International* **2015**, *2015*, doi:10.1155/2015/618752.
15. Kakooza, S.; Munyiirwa, D.; Ssajjakambwe, P.; Kayaga, E.; Tayebwa, D.S.; Ndoboli, D.; Basemera, L.; Nabatta, E.; Tumwebaze, M.A.; Kaneene, J.B. Epidemiological Dynamics of Extended-Spectrum β -Lactamase- or AmpC β -Lactamase-Producing Escherichia Coli Screened in Apparently Healthy Chickens in Uganda. *Scientifica* **2021**, *2021*, doi:10.1155/2021/3258059.
16. Nguyen, V.T.; Carrique-Mas, J.J.; Ngo, T.H.; Ho, H.M.; Ha, T.T.; Campbell, J.I.; Nguyen, T.N.; Hoang, N.N.; Pham, V.M.; Wagenaar, J.A.; et al. Prevalence and Risk Factors for Carriage of Antimicrobial-Resistant Escherichia Coli on Household and Small-Scale Chicken Farms in the Mekong Delta of Vietnam. *Journal of Antimicrobial Chemotherapy* **2015**, *70*, 2144–2152, doi:10.1093/jac/dkv053.
17. Pham-Duc, P.; Cook, M.A.; Cong-Hong, H.; Nguyen-Thuy, H.; Padungtod, P.; Nguyen-Thi, H.; Dang-Xuan, S. Knowledge, Attitudes and Practices of Livestock and Aquaculture Producers Regarding Antimicrobial Use and Resistance in Vietnam. *PLoS ONE* **2019**, *14*, doi:10.1371/journal.pone.0223115.
18. Carrique-Mas, J.J.; Trung, N.V.; Hoa, N.T.; Mai, H.H.; Thanh, T.H.; Campbell, J.I.; Wagenaar, J.A.; Hardon, A.; Hieu, T.Q.; Schultsz, C. Antimicrobial Usage in Chicken Production in the Mekong Delta of Vietnam. *Zoonoses and Public Health* **2015**, *62*, 70–78, doi:10.1111/zph.12165.
19. Caffrey, N.; Agunos, A.; Gow, S.; Liljebjelke, K.; Waldner, C.L.; Mainali, C.; Checkley, S.L. A Cross-Sectional Study of the Prevalence Factors Associated with Fluoroquinolone Resistant Campylobacter Jejuni in Broiler Flocks in Canada. *Preventive Veterinary Medicine* **2021**, *186*, doi:10.1016/j.prevetmed.2020.105164.
20. Bhargavi, D.; Sahu, R.; Nishanth, M.A.D.; Doijad, S.P.; Niveditha, P.; Kumar, O.R.V.; Sunanda, C.; Girish, P.S.; Naveena, B.M.; Vergis, J.; et al. Genetic Diversity and Risk

Factor Analysis of Drug-Resistant Escherichia Coli Recovered from Broiler Chicken Farms. *Comparative Immunology, Microbiology and Infectious Diseases* **2023**, *93*, doi:10.1016/j.cimid.2022.101929.
21. Moffo, F.; Mouliom Mouiche, M.M.; Kochivi, F.L.; Dongmo, J.B.; Djomgang, H.K.; Tombe, P.; Mbah, C.K.; Mapiefou, N.P.; Mingoas, J.-P.K.; Awah-Ndukum, J. Knowledge, Attitudes, Practices and Risk Perception of Rural Poultry Farmers in Cameroon to Antimicrobial Use and Resistance. *Preventive Veterinary Medicine* **2020**, *182*, doi:10.1016/j.prevetmed.2020.105087.
22. Buberg, M.L.; Witsø, I.L.; L'Abée-Lund, T.M.; Wasteson, Y. Zinc and Copper Reduce Conjugative Transfer of Resistance Plasmids from Extended-Spectrum Beta-Lactamase-Producing Escherichia Coli. *Microbial Drug Resistance* **2020**, *26*, 842–849, doi:10.1089/mdr.2019.0388.
23. Montoro-Dasi, L.; Villagra, A.; Sevilla-Navarro, S.; Pérez-Gracia, M.T.; Vega, S.; Marin, C. The Dynamic of Antibiotic Resistance in Commensal Escherichia Coli throughout the Growing Period in Broiler Chickens: Fast-Growing vs. Slow-Growing Breeds. *Poultry Science* **2020**, *99*, 1591–1597, doi:10.1016/j.psj.2019.10.080.
24. Moffo, F.; Mouiche, M.M.M.; Djomgang, H.K.; Tombe, P.; Wade, A.; Kochivi, F.L.; Dongmo, J.B.; Mbah, C.K.; Mapiefou, N.P.; Ngogang, M.P.; et al. Poultry Litter Contamination by Escherichia Coli Resistant to Critically Important Antimicrobials for Human and Animal Use and Risk for Public Health in Cameroon. *Antibiotics* **2021**, *10*, doi:10.3390/antibiotics10040402.
25. Taylor, N.M.; Wales, A.D.; Ridley, A.M.; Davies, R.H. Farm Level Risk Factors for Fluoroquinolone Resistance in E. Coli and Thermophilic Campylobacter Spp. on Poultry Farms. *Avian Pathology* **2016**, *45*, 559–568, doi:10.1080/03079457.2016.1185510.
26. Mandal, A.K.; Talukder, S.; Hasan, M.M.; Tasmim, S.T.; Parvin, M.S.; Ali, M.Y.; Islam, M.T. Epidemiology and Antimicrobial Resistance of Escherichia Coli in Broiler Chickens, Farmworkers, and Farm Sewage in Bangladesh. *Veterinary Medicine and Science* **2022**, *8*, 187–199, doi:10.1002/vms3.664.
27. Caudell, M.A.; Dorado-Garcia, A.; Eckford, S.; Creese, C.; Byarugaba, D.K.; Afakye, K.; Chansa-Kabali, T.; Fasina, F.O.; Kabali, E.; Kiambi, S.; et al. Towards a Bottom-up Understanding of Antimicrobial Use and Resistance on the Farm: A Knowledge, Attitudes, and Practices Survey across Livestock Systems in Five African Countries. *PLoS ONE* **2020**, *15*, doi:10.1371/journal.pone.0220274.
28. Mo, S.S.; Kristoffersen, A.B.; Sunde, M.; Nødtvedt, A.; Norström, M. Risk Factors for Occurrence of Cephalosporin-Resistant Escherichia Coli in Norwegian Broiler Flocks. *Preventive Veterinary Medicine* **2016**, *130*, 112–118, doi:10.1016/j.prevetmed.2016.06.011.
29. Huber, L.; Agunos, A.; Gow, S.P.; Carson, C.A.; Van Boeckel, T.P. Reduction in Antimicrobial Use and Resistance to Salmonella, Campylobacter, and Escherichia Coli in Broiler Chickens, Canada, 2013-2019. *Emerging Infectious Diseases* **2021**, *27*, 2434–2444, doi:10.3201/eid2709.204395.
30. Mezhoud, H.; Chantziaras, I.; Iguer-Ouada, M.; Moula, N.; Garmyn, A.; Martel, A.; Touati, A.; Smet, A.; Haesebrouck, F.; Boyen, F. Presence of Antimicrobial Resistance in Coliform Bacteria from Hatching Broiler Eggs with Emphasis on ESBL/AmpC-Producing Bacteria. *Avian Pathology* **2016**, *45*, 493–500, doi:10.1080/03079457.2016.1167837.
31. Skarzynska, M.; Leekitcharoenphon, P.; Hendriksen, R.S.; Aarestrup, F.M.; Wasyl, D. A Metagenomic Glimpse into the Gut of Wild and Domestic Animals: Quantification of

Antimicrobial Resistance and More. *PLoS ONE* **2020**, *15*, doi:10.1371/journal.pone.0242987.
32. Oloso, N.O.; Odetokun, I.A.; Ghali-Mohammed, I.; Fasina, F.O.; Olatoye, I.O.; Adetunji, V.O. Knowledge, Attitudes, and Risk Perception of Broiler Grow-Out Farmers on Antimicrobial Use and Resistance in Oyo State, Nigeria. *Antibiotics* **2022**, *11*, doi:10.3390/antibiotics11050567.
33. Makarov, D.A.; Ivanova, O.E.; Pomazkova, A.V.; Egoreva, M.A.; Prasolova, O.V.; Lenev, S.V.; Gergel, M.A.; Bukova, N.K.; Karabanov, S.Y. Antimicrobial Resistance of Commensal Enterococcus Faecalis and Enterococcus Faecium from Food-Producing Animals in Russia. *Veterinary World* **2022**, *15*, 611–621, doi:10.14202/vetworld.2022.611-621.
34. Bythwood, T.N.; Soni, V.; Lyons, K.; Hurley-Bacon, A.; Lee, M.D.; Hofacre, C.; Sanchez, S.; Maurer, J.J. Antimicrobial Resistant Salmonella Enterica Typhimurium Colonizing Chickens: The Impact of Plasmids, Genotype, Bacterial Communities, and Antibiotic Administration on Resistance. *Frontiers in Sustainable Food Systems* **2019**, *3*, doi:10.3389/fsufs.2019.00020.
35. Luiken, R.E.C.; Van Gompel, L.; Munk, P.; Sarrazin, S.; Joosten, P.; Dorado-García, A.; Borup Hansen, R.; Knudsen, B.E.; Bossers, A.; Wagenaar, J.A.; et al. Associations between Antimicrobial Use and the Faecal Resistome on Broiler Farms from Nine European Countries. *Journal of Antimicrobial Chemotherapy* **2019**, *74*, 2596–2604, doi:10.1093/jac/dkz235.
36. Shang, K.; Wei, B.; Kang, M. Distribution and Dissemination of Antimicrobial-Resistant Salmonella in Broiler Farms with or without Enrofloxacin Use. *BMC Veterinary Research* **2018**, *14*, doi:10.1186/s12917-018-1590-1.
37. Koju, P.; Shrestha, R.; Shrestha, A.; Tamrakar, S.; Rai, A.; Shrestha, P.; Madhup, S.K.; Katuwal, N.; Shrestha, A.; Shrestha, A.; et al. Antimicrobial Resistance in E. Coli Isolated from Chicken Cecum Samples and Factors Contributing to Antimicrobial Resistance in Nepal. *Tropical Medicine and Infectious Disease* **2022**, *7*, doi:10.3390/tropicalmed7090249.
38. Ayoola, M.B.; Pillai, N.; Nanduri, B.; Rothrock, M.J.; Ramkumar, M. Preharvest Environmental and Management Drivers of Multidrug Resistance in Major Bacterial Zoonotic Pathogens in Pastured Poultry Flocks. *Microorganisms* **2022**, *10*, doi:10.3390/microorganisms10091703.
39. Nguyen, P.T.L.; Ngo, T.H.H.; Tran, T.M.H.; Vu, T.N.B.; Le, V.T.; Tran, H.A.; Pham, D.T.; Nguyen, H.T.; Tran, D.L.; Nguyen, T.P.L.; et al. Genomic Epidemiological Analysis of Mcr-1-Harboring Escherichia Coli Collected from Livestock Settings in Vietnam. *Frontiers in Veterinary Science* **2022**, *9*, doi:10.3389/fvets.2022.1034610.
40. Jiménez-Belenguer, A.; Doménech, E.; Villagrá, A.; Fenollar, A.; Ferrús, M.A. Antimicrobial Resistance of Escherichia Coli Isolated in Newly-Hatched Chickens and Effect of Amoxicillin Treatment during Their Growth. *Avian Pathology* **2016**, *45*, 501–507, doi:10.1080/03079457.2016.1168515.
41. Abraham, S.; Sahibzada, S.; Hewson, K.; Laird, T.; Abraham, R.; Pavic, A.; Truswell, A.; Lee, T.; O'Dea, M.; Jordan, D. Emergence of Fluoroquinolone-Resistant Campylobacter Jejuni and Campylobacter Coli among Australian Chickens in the Absence of Fluoroquinolone Use. *Applied and Environmental Microbiology* **2020**, *86*, doi:10.1128/AEM.02765-19.
42. Oladeinde, A.; Abdo, Z.; Press, M.O.; Cook, K.; Cox, N.A.; Zwirzitz, B.; Woyda, R.; Lakin, S.M.; Thomas Iv, J.C.; Looft, T.; et al. Horizontal Gene Transfer Is the Main

Driver of Antimicrobial Resistance in Broiler Chicks Infected with Salmonella Enterica Serovar Heidelberg. *mSystems* **2021**, *6*, doi:10.1128/mSystems.00729-21.
43. Talavera-González, J.M.; Talavera-Rojas, M.; Soriano-Vargas, E.; Vázquez-Navarrete, J.; Salgado-Miranda, C. In Vitro Transduction of Antimicrobial Resistance Genes into Escherichia Coli Isolates from Backyard Poultry in Mexico. *Canadian Journal of Microbiology* **2021**, *67*, 415–425, doi:10.1139/cjm-2020-0280.
44. Talukder, S.; Hasan, M.M.; Mandal, A.K.; Tasmim, S.T.; Parvin, M.S.; Ali, M.Y.; Nahar, A.; Islam, M.Z.; Islam, M.T. Epidemiology and Antimicrobial Resistance Profiles of Salmonella in Chickens, Sewage, and Workers of Broiler Farms in Selected Areas of Bangladesh. *Journal of Infection in Developing Countries* **2021**, *15*, 1155–1166, doi:10.3855/jidc.14100.
45. Khan, X.; Lim, R.H.M.; Rymer, C.; Ray, P. Fijian Farmers' Attitude and Knowledge Towards Antimicrobial Use and Antimicrobial Resistance in Livestock Production Systems–A Qualitative Study. *Frontiers in Veterinary Science* **2022**, *9*, doi:10.3389/fvets.2022.838457.
46. Lowenstein, C.; Waters, W.F.; Roess, A.; Leibler, J.H.; Graham, J.P. Animal Husbandry Practices and Perceptions of Zoonotic Infectious Disease Risks among Livestock Keepers in a Rural Parish of Quito, Ecuador. *American Journal of Tropical Medicine and Hygiene* **2016**, *95*, 1450–1458, doi:10.4269/ajtmh.16-0485.
47. Speksnijder, D.C.; Jaarsma, A.D.C.; van der Gugten, A.C.; Verheij, T.J.M.; Wagenaar, J.A. Determinants Associated with Veterinary Antimicrobial Prescribing in Farm Animals in the Netherlands: A Qualitative Study. *Zoonoses and Public Health* **2015**, *62*, 39–51, doi:10.1111/zph.12168.
48. Benavides, J.A.; Streicker, D.G.; Gonzales, M.S.; Rojas-Paniagua, E.; Shiva, C. Knowledge and Use of Antibiotics among Low-Income Small-Scale Farmers of Peru. *Preventive Veterinary Medicine* **2021**, *189*, doi:10.1016/j.prevetmed.2021.105287.
49. Sawadogo, A.; Kagambèga, A.; Moodley, A.; Ouedraogo, A.A.; Barro, N.; Dione, M. Knowledge, Attitudes, and Practices Related to Antibiotic Use and Antibiotic Resistance among Poultry Farmers in Urban and Peri-Urban Areas of Ouagadougou, Burkina Faso. *Antibiotics* **2023**, *12*, doi:10.3390/antibiotics12010133.
50. Alhaji, N.B.; Haruna, A.E.; Muhammad, B.; Lawan, M.K.; Isola, T.O. Antimicrobials Usage Assessments in Commercial Poultry and Local Birds in North-Central Nigeria: Associated Pathways and Factors for Resistance Emergence and Spread. *Preventive Veterinary Medicine* **2018**, *154*, 139–147, doi:10.1016/j.prevetmed.2018.04.001.
51. Luu, Q.H.; Nguyen, T.L.A.; Pham, T.N.; Vo, N.G.; Padungtod, P. Antimicrobial Use in Household, Semi-Industrialized, and Industrialized Pig and Poultry Farms in Viet Nam. *Preventive Veterinary Medicine* **2021**, *189*, doi:10.1016/j.prevetmed.2021.105292.
52. Islam, Md.Z.; Islam, Md.S.; Kundu, L.R.; Ahmed, A.; Hsan, K.; Pardhan, S.; Driscoll, R.; Hossain, Md.S.; Hossain, Md.M. Knowledge, Attitudes and Practices Regarding Antimicrobial Usage, Spread and Resistance Emergence in Commercial Poultry Farms of Rajshahi District in Bangladesh. *PLoS ONE* **2022**, *17*, doi:10.1371/journal.pone.0275856.
53. Hassan, M.M.; Kalam, M.A.; Alim, M.A.; Shano, S.; Nayem, M.R.K.; Badsha, M.R.; Mamun, M.A.A.; Hoque, A.; Tanzin, A.Z.; Nath, C.; et al. Knowledge, Attitude, and Practices on Antimicrobial Use and Antimicrobial Resistance among Commercial Poultry Farmers in Bangladesh. *Antibiotics* **2021**, *10*, doi:10.3390/antibiotics10070784.

54. Poupaud, M.; Goutard, F.L.; Phouthana, V.; Muñoz Viera, F.; Caro, D.; Patriarchi, A.; Paul, M.C. Different Kettles of Fish: Varying Patterns of Antibiotic Use on Pig, Chicken and Fish Farms in Lao PDR and Implications for Antimicrobial Resistance Strategies. *Transboundary and Emerging Diseases* **2022**, *69*, 3940–3951, doi:10.1111/tbed.14766.
55. Nhung, N.T.; Cuong, N.V.; Campbell, J.; Hoa, N.T.; Bryant, J.E.; Truc, V.N.T.; Kiet, B.T.; Jombart, T.; Trung, N.V.; Hien, V.B.; et al. High Levels of Antimicrobial Resistance among Escherichia Coli Isolates from Livestock Farms and Synanthropic Rats and Shrews in the Mekong Delta of Vietnam. *Applied and Environmental Microbiology* **2015**, *81*, 812–820, doi:10.1128/AEM.03366-14.
56. Mankhomwa, J.; Tolhurst, R.; M'biya, E.; Chikowe, I.; Banda, P.; Mussa, J.; Mwasikakata, H.; Simpson, V.; Feasey, N.; MacPherson, E.E. A Qualitative Study of Antibiotic Use Practices in Intensive Small-Scale Farming in Urban and Peri-Urban Blantyre, Malawi: Implications for Antimicrobial Resistance. *Frontiers in Veterinary Science* **2022**, *9*, doi:10.3389/fvets.2022.876513.
57. Martínez, E.P.; Golding, S.E.; van Rosmalen, J.; Vinueza-Burgos, C.; Verbon, A.; van Schaik, G. Antibiotic Prescription Patterns and Non-Clinical Factors Influencing Antibiotic Use by Ecuadorian Veterinarians Working on Cattle and Poultry Farms: A Cross-Sectional Study. *Preventive Veterinary Medicine* **2023**, *213*, doi:10.1016/j.prevetmed.2023.105858.
58. Mouiche, M.M.M.; Wouembe, F.D.K.; Mpouam, S.E.; Moffo, F.; Djuntu, M.; Toukam, C.M.W.; Kameni, J.M.F.; Okah-Nnane, N.H.; Awah-Ndukum, J. Cross-Sectional Survey of Prophylactic and Metaphylactic Antimicrobial Use in Layer Poultry Farming in Cameroon: A Quantitative Pilot Study. *Frontiers in Veterinary Science* **2022**, *9*, doi:10.3389/fvets.2022.646484.
59. Coyne, L.; Patrick, I.; Arief, R.; Benigno, C.; Kalpravidh, W.; McGrane, J.; Schoonman, L.; Sukarno, A.H.; Rushton, J. The Costs, Benefits and Human Behaviours for Antimicrobial Use in Small Commercial Broiler Chicken Systems in Indonesia. *Antibiotics* **2020**, *9*, doi:10.3390/antibiotics9040154.
60. Caucci, C.; Di Martino, G.; Dalla Costa, A.; Santagiuliana, M.; Lorenzetto, M.; Capello, K.; Mughini-Gras, L.; Gavazzi, L.; Bonfanti, L. Trends and Correlates of Antimicrobial Use in Broiler and Turkey Farms: A Poultry Company Registry-Based Study in Italy. *Journal of Antimicrobial Chemotherapy* **2019**, *74*, 2784–2787, doi:10.1093/jac/dkz212.
61. Pavez-Muñoz, E.; González, C.; Fernández-Sanhueza, B.; Sánchez, F.; Escobar, B.; Ramos, R.; Fuenzalida, V.; Galarce, N.; Arriagada, G.; Neira, V.; et al. Antimicrobial Usage Factors and Resistance Profiles of Shiga Toxin-Producing Escherichia Coli in Backyard Production Systems From Central Chile. *Frontiers in Veterinary Science* **2021**, *7*, doi:10.3389/fvets.2020.595149.
62. Kalam, M.A.; Rahman, M.S.; Alim, M.A.; Shano, S.; Afrose, S.; Jalal, F.A.; Akter, S.; Khan, S.A.; Islam, M.M.; Uddin, M.B.; et al. Knowledge, Attitudes, and Common Practices of Livestock and Poultry Veterinary Practitioners Regarding the AMU and AMR in Bangladesh. *Antibiotics* **2022**, *11*, doi:10.3390/antibiotics11010080.
63. Bastard, J.; Nhung, N.T.; Hien, V.B.; Kiet, B.T.; Temime, L.; Opatowski, L.; Carrique-Mas, J.; Choisy, M. Modelling the Impact of Antimicrobial Use and External Introductions on Commensal E. Coli Colistin Resistance in Small-Scale Chicken Farms of the Mekong Delta of Vietnam. *Transboundary and Emerging Diseases* **2022**, *69*, e2185–e2194, doi:10.1111/tbed.14558.

64. Oloso, N.O.; Adeyemo, I.A.; Heerden, H.V.; Fasanmi, O.G.; Fasina, F.O. Antimicrobial Drug Administration and Antimicrobial Resistance of Salmonella Isolates Originating from the Broiler Production Value Chain in Nigeria. *Antibiotics* **2019**, *8*, doi:10.3390/antibiotics8020075.
65. Chowdhury, S.; Fournié, G.; Blake, D.; Henning, J.; Conway, P.; Hoque, MdA.; Ghosh, S.; Parveen, S.; Biswas, P.K.; Akhtar, Z.; et al. Antibiotic Usage Practices and Its Drivers in Commercial Chicken Production in Bangladesh. *PLoS ONE* **2022**, *17*, doi:10.1371/journal.pone.0276158.
66. Bâtie, C.; Ha, L.T.T.; Loire, E.; Truong, D.B.; Tuan, H.M.; Cuc, N.T.K.; Paul, M.; Goutard, F. Characterisation of Chicken Farms in Vietnam: A Typology of Antimicrobial Use among Different Production Systems. *Preventive Veterinary Medicine* **2022**, *208*, doi:10.1016/j.prevetmed.2022.105731.
67. Eltholth, M.; Govindaraj, G.; Das, B.; Shanabhoga, M.B.; Swamy, H.M.; Thomas, A.; Cole, J.; Shome, B.R.; Holmes, M.A.; Moran, D. Factors Influencing Antibiotic Prescribing Behavior and Understanding of Antimicrobial Resistance Among Veterinarians in Assam, India. *Frontiers in Veterinary Science* **2022**, *9*, doi:10.3389/fvets.2022.864813.
68. Dione, M.M.; Amia, W.C.; Ejobi, F.; Ouma, E.A.; Wieland, B. Supply Chain and Delivery of Antimicrobial Drugs in Smallholder Livestock Production Systems in Uganda. *Frontiers in Veterinary Science* **2021**, *8*, doi:10.3389/fvets.2021.611076.
69. Kalam, Md.A.; Alim, Md.A.; Shano, S.; Nayem, Md.R.K.; Badsha, Md.R.; Mamun, Md.A.A.; Hoque, A.; Tanzin, A.Z.; Khan, S.A.; Islam, A.; et al. Knowledge, Attitude, and Practices on Antimicrobial Use and Antimicrobial Resistance among Poultry Drug and Feed Sellers in Bangladesh. *Veterinary Sciences* **2021**, *8*, 111–111, doi:10.3390/vetsci8060111.
